# A multi-omics exploration of PPARG activation in colon cancer: kinases featuring a PPRE sequence within regulatory regions

**DOI:** 10.1186/s13062-025-00654-7

**Published:** 2025-06-11

**Authors:** Pritha Saha, Palaniyandi Ravanan, Priti Talwar

**Affiliations:** 1 Apoptosis and Cell Survival Research Laboratory, 412G Pearl Research Park, School of Biosciences and Technology, Vellore Institute of Technology, Vellore, Tamil Nadu 632014 India; 2 Functional Genomics Laboratory, Department of Microbiology, School of Life Sciences, Central University of Tamil Nadu, Thiruvarur, Tamil Nadu 610005 India

**Keywords:** Peroxisome Proliferator-Activated Receptors-γ, Kinase, Gene enrichment, Network analysis, Mi-RNA, R studio, RNA-seq, ChIP-seq

## Abstract

**Background:**

As members of the nuclear receptor (NR) family of transcription factors, peroxisome proliferator-activated receptors (PPARs) regulate essential cellular processes, including lipid metabolism, glucose uptake, cell proliferation, and programmed cell death through ligand-mediated activation. Within the PPAR subfamilies, PPAR-γ (PPARG) is crucial to the development of fat cells, sensitivity to insulin, apoptosis, and metastasis. Furthermore, it demonstrates properties that counteract fibrosis and inflammation, thus establishing itself as a notable target for therapeutic interventions against conditions such as type 2 diabetes and cancer. PPARG is reported to be a promising target for patients diagnosed with colorectal cancer (CRC). Globally, colorectal cancer ranks as the third most prevalent malignancy and is responsible for approximately 10% of all cancer mortalities, and PPARG is significantly expressed in 70% of the sporadic CRC. In individuals with CRC, the precise function of PPARG remains not entirely comprehended and elucidation of the PPARG transcriptional regulation in CRC seems promising.

**Results:**

This study integrates RNA-seq and ChIP-seq reads to analyze the effects of Rosiglitazone on HT-29 colon cancer cells. Peak calling analysis from ChIP-seq data identified 14,000 to 34,000 binding sites for PPARG across different experimental conditions. RNA-seq analysis highlighted significant differential gene expression in Rosiglitazone-treated cells, with 4362 and 6780 genes significantly regulated at 24 and 48 h, respectively. The correlation of these datasets with PPRE-associated kinases resulted in the identification of 18 differentially expressed genes (DEGs), followed by subsequent analysis of gene ontology, pathway enrichment, and protein–protein interactions, culminating in the elucidation of seven hub genes (PTK2, HGS, CDK8, PRPF6, PRKDC, PRKCZ, MET). Further these hub genes correlated with CRC progression and patient survival. Validation using independent GEO datasets (GSE113513 and GSE210693) and gene effect scores derived from CRISPR knockout screens further supported the functional impact of these hub genes. Disease ontology and mutational analyses implicated the hub genes in various cancers, including CRC. Moreover, miRNA analysis identified 37 experimentally validated miRNAs potentially modulating hub gene expression.

**Conclusions:**

These findings advance our understanding of PPARG's regulatory network and underscore its potential as a therapeutic target, establishing a robust framework for future research in PPARG-related pathways.

**Supplementary Information:**

The online version contains supplementary material available at 10.1186/s13062-025-00654-7.

## Background

“Peroxisome Proliferator-Activated Receptors” (PPARs) play a pivotal role in the modulation of genes that govern intracellular lipid metabolism, glucose absorption, apoptosis, and cellular proliferation. These transcription factors, which belong to the Nuclear Receptor (NR) family, are subdivided into three distinct isoforms: α, β/δ, and γ [1]. Following the binding of specific activators, PPARs form heterodimers with Retinoid X Receptors (RXR), and this resultant complex engages with the “PPAR response element” (PPRE) (consensus sequence 5’AGGTCANAGGTCA-3’), thereby initiating the transcriptional regulation of target genes [2]. The diverse responses exhibited by these receptors in reaction to various ligands enable them to modulate gene expression and oversee a multitude of cellular processes [3–5]. PPAR-modulated cellular activities have been associated with multiple diseases like type 2 diabetes mellitus, neurodegenerative disorders, cancer, and pulmonary fibrosis. PPAR-γ (PPARG) is a key protein involved in adipogenesis, insulin sensitivity regulation, and pulmonary homeostasis. It is expressed in various cell types and has anti-fibrotic properties. Studies show that attenuating PPARG expression in isolated lung fibroblasts can increase pro-fibrotic characteristics [6]. It also has anti-inflammatory properties. Due to its influence on these processes, PPARG is being investigated as a target for treating diseases like type 2 diabetes and even cancer [7, 8].

PPARG is reported to be a promising target for patients diagnosed with colorectal cancer (CRC) [9]. Elevated levels of PPARG are observed in human colon carcinoma cell lines. Globally, CRC ranks as the third most prevalent malignancy and is responsible for approximately 10% of all mortalities attributable to cancer [10], and 70% of the sporadic CRC exhibits substantial PPARG regulation [11]. PPARG agonists (e.g. rosiglitazone, troglitazone) are substantially involved in cell cycle inhibition, differentiation, proliferation, and migration of tumor cells [12, 13]. PPARG has been demonstrated to operate as a tumor suppressor by modulating essential biological pathways: metastatic dissemination, suppression of cellular proliferation, and apoptosis across diverse cancer cell lineages. Furthermore, it facilitates intercellular adhesion and alleviates the inflammatory condition prevalent within the tumor microenvironment, affecting both transcriptional and protein expression levels [14, 15]. Studies have reported that administration of PPARG ligands (pioglitazone, troglitazone) in colorectal carcinoma cells enhances the expression of the Bax protein and thus enhances the apoptosis while simultaneously diminishing the expression of Bcl-2, a gene inhibiting programmed cell death [10]. PPARG has been suggested to have both tumor-suppressive [16] and tumor-facilitating [17, 18]roles in colorectal cancer. Pioglitazone (agonist of PPARG) stimulates the growth of Adenomatous Polyposis Coli (APC)-mutated HT-29 human colon cancer cells [19], while troglitazone enhances colon cancer in normal C57BL/6 J mice [20]. It has been demonstrated that pioglitazone stimulates the growth of tumors in Apc^Min/1^ mice [21]. Conversely, troglitazone caused cell cycle arrest, apoptosis, and inhibition of growth in colon cancer cell HCT-116 [22]. The administration of rosiglitazone, a recognized agonist of PPARG, to colon cancer cells results in the upregulation of the tumor suppressor gene PTEN [23]. Another research demonstrates the prognostic impact of the receptor is influenced by the site-specific localization of metastatic CRC. The administration of pioglitazone and rosiglitazone leads to an increase in CRC cell proliferation, whereas the suppression mediated by GW9662 and siRNA-induced knockdown reduces cellular proliferation. On the contrary, the activation of PPARG enhances the susceptibility to the cytotoxic agent 5-fluorouracil (5-FU), while reduced sensitivity is observed with its inhibition [24].

In this study, we have analyzed the RNA-seq and ChIP-seq datasets (GSE77039) on rosiglitazone-treated HT-29 cells. At present, the precise function of PPARG in individuals with CRC remains not fully comprehended, and elucidation of the PPARG transcriptional regulation in CRC seems promising. While analysis of the ChIP-seq dataset reveals the binding profile of PPARG in human chromosomes, analysis of the RNA-seq dataset elucidates the gene regulation through PPARG. Further, the results from RNA-seq and ChIP-seq analysis were correlated with the PPRE-associated kinases, and 18 differentially expressed genes (DEGs) were obtained, which were subsequently subjected to analyses concerning gene ontology and biological pathway involvement, and their association with human protein–protein interactome (PPI) was explored, followed by hub gene identification based on parameters, i.e. closeness, degree, and betweenness. These hub genes are also correlated with CRC progression and patient survival. Validation using additional GEO datasets and gene effect from CRISPR screens further supported the regulatory impact of PPARG. Disease ontology and mutational analyses implicated the hub genes in various cancers, including CRC. Moreover, miRNA analysis identified experimentally validated miRNAs potentially modulating hub gene expression.

## Methodology

### Data acquisition for ChIP-seq and RNA-seq analysis

The European Nucleotide Archive (ENA), which is overseen by the European Bioinformatics Institute (EBI), serves as an extensive repository for nucleotide sequence data [25]. Within the scope of this investigation, the FASTQ data associated with the study GSE77039 were obtained [26], from the ENA database. The analysis conducted in this study involved the investigation of RNA-seq reads derived from HT-29 cells treated with Rosiglitazone for 24 and 48 h (including 4 replicates each) (Table 1), as well as ChIP-seq reads against PPARG antibody obtained from the same cell type after treatment with Rosiglitazone for 2 and 48 h (including 2 replicates each) (Table 1).

**Table 1:** Accession numbers of the ChIP-seq and RNA-seq datasets

Experiment	Accession number	Treatment
ChIP-seq (PPARG)	GSM2042854	2 h Rosiglitazone- Rep1
	GSM2042856	2 h Rosiglitazone- Rep2
	GSM2042858	48 h Rosiglitazone- Rep1
	GSM2042860	48 h Rosiglitazone- Rep2
	GSM2042870	2 h Rosiglitazone- Input ctrl
	GSM2042872	48 h Rosiglitazone- Input ctrl
RNA-seq	GSM2042916	24H DMSO-Rep1
	GSM2042917	24H DMSO-Rep2
	GSM2042918	24H DMSO-Rep3
	GSM2042919	24H DMSO-Rep4
	GSM2042929	24H Rosiglitazone-Rep1
	GSM2042930	24H Rosiglitazone-Rep2
	GSM2042931	24H Rosiglitazone-Rep3
	GSM2042932	24H Rosiglitazone-Rep4
	GSM2042933	48H DMSO-Rep1
	GSM2042934	48H DMSO-Rep2
	GSM2042935	48H DMSO-Rep3
	GSM2042936	48H DMSO-Rep4
	GSM2042945	48H Rosiglitazone-Rep1
	GSM2042946	48H Rosiglitazone-Rep2
	GSM2042947	48H Rosiglitazone-Rep3
	GSM2042948	48H Rosiglitazone-Rep4

### ChIP-seq data analysis: PPARG binding profile in human chromosome

The study used 'Rsubread' to create a reference index for ChIP-seq reads, and the 'BSgenome.Hsapiens.UCSC.hg38' package for the reference genome sequence [27, 28]. MACS2 (Model-based Analysis of ChIP-seq), a standard algorithm for discerning enriched genomic regions in ChIP-seq data, was utilized for the peak calling [29]. Peak annotation was carried out using the ‘ChIPseeker’ package [30]. Peaks were annotated to known genes using the ‘annotatePeak’ function, using packages like ‘TxDb.Hsapiens.UCSC.hg38.knownGene’ and ‘org.Hs.eg.db’ [31, 32].

### RNA-seq data analysis: indexing, alignment, differential expression analysis

The 'BSgenome.Hsapiens.UCSC.hg38' package was used to create an index for the FASTQ reads of the RNA-seq experiments, using the ‘Rsubread’ package, and the reads were further aligned with the indexed reference genome [27, 28, 31]. The aligned BAM files were then processed to count reads overlapping the gene exons, using the ‘GenomicAlignments’ package [33].

The analysis of differential gene expression was conducted utilizing the 'DESeq2' software package [34]. Utilizing the ‘EnhancedVolcano’ package a volcano plot of the gene expression results was generated. Heatmaps illustrating the 50 most significantly differentially expressed genes (DEGs), as determined by adjusted p-values (padj), were generated utilizing the ‘ggplot2’ package [35, 36].

### Comparative analysis of ChIP-seq and RNA-seq data with identified PPRE-associated genes

The genes identified from the peaks observed in each ChIP-seq sample were juxtaposed with the gene list obtained from our previous study of genome-wide identification of PPRE-associated genes and kinases [2]. A comparative analysis was also conducted between the differentially expressed genes from RNA-seq datasets following 24 h and 48 h Rosiglitazone treatment, and the PPRE-associated genes [2]. Together, these prevalent genes signify the notably altered genes, featuring a PPRE sequence within their regulatory regions along the PPARG pathways associated with colon cancer.

### Functional pathway enrichment analysis

The PPRE-associated DEGs (kinases) were further utilized as queries for the Functional enrichment study. The Gene Ontology (GO) of the hits regulated at both 24 and 48 h and only 48 h was elucidated in following three terms, i.e., Biological Process (BP), Molecular Function (MF), and Cellular Component (CC), using SRplot [37] and the pathway analysis was performed using the FunRich database [38].

### PPI network construction and Hub genes prediction

The NDEx Project (Network Data Exchange) constitutes an open-source framework meticulously engineered to enhance the sharing, visualization, and storage of biological networks [39]. The NDEx server was employed to acquire the high-confidence (≥ 0.7) interaction network (all experimentally validated) of the PPRE-associated DEGs (kinases) within HIPPIE—Human Scored Interactions (version 2.3), an extensive human PPI network [40]. On the basis of the network analysis parameters, closeness, degree, and betweenness, the hub genes within the interacting network were elucidated using the cytoHubba plugin integrated into Cytoscape [41].

### Correlation of the hub genes with CRC

UALCAN is an interactive web resource for analyzing cancer transcriptome datasets. It allows users to explore gene expression, survival analysis, and tumor subgroup comparisons across various cancer types [42]. The association of the hub genes with different stages of CRC was assessed using the UALCAN database. It also provides gene effect scores obtained from integrated CRISPR knockout screens conducted by the Broad Institute’s Achilles project and the Sanger Institute’s SCORE project [43, 44]. The knockout effect analysis of the hub genes across different CRC cell lines was performed. The Kaplan–Meier Plotter is a comprehensive online tool designed to evaluate the prognostic significance of genes across various cancer types. It integrates gene expression data with survival information, enabling researchers to generate survival plots and assess the impact of specific genes on patient outcomes [45]. The survival probability of CRC patients (n = 1336) in relation to the hub genes expression level was analyzed using this web tool.

### Dataset analysis for validation

NCBI Gene Expression Omnibus (GEO) is a public repository that archives and freely distributes high-throughput gene expression and other functional genomics data. Researchers can access curated datasets for reanalysis or comparison across various biological conditions and experimental platforms [46]. The microarray data (GSE113513) from fourteen samples, each of CRC patients and normal tissue [47], were analyzed using GEO2R, an interactive online tool provided by NCBI GEO that allows users to compare gene expression between sample groups in a GEO dataset. It uses the R-based limma package to identify differentially expressed genes with associated statistical metrics [48]. This analysis provides insights into the differential expression of the hub genes in the CRC patients.

To further validate that the hub genes are regulated through PPARG activity, we analyzed an additional dataset (GSE210693) using PPARG antagonist T0070907 [49]. The TPM-normalized gene count matrix was obtained, and the regulation of the hub genes across DMSO, Rosiglitazone and T0070907 treatment was plotted in a cluster heatmap using SRplot [37].

### Disease association of the hub genes

Disease Ontology (DO) serves to annotate human genes within the framework of diseases, thereby enhancing the translation of molecular discoveries into clinical understanding. The R package DOSE offers methodologies for the computation of semantic similarities between DO terms and genes, executing enrichment analyses, and facilitating the comparison of gene clusters [50]. The link between the hub genes and human diseases was analyzed using the DOSE package in R Studio, and a heatmap of the significant gene-disease associations (*p* ≤ 0.05) was created [51]. The UniProt database is a comprehensive protein sequence and functional information resource, widely used in bioinformatics and molecular biology. Its Variant Viewer tool allows users to explore and visualize natural and disease-associated variants in protein sequences, helping to understand their potential biological impact [51]. UniProt database was used to identify the mutations in the hub genes associated with carcinogenesis. The variant viewer was utilized to obtain relevant mutations that are likely pathogenic or pathogenic with high impact.

### miRNA target prediction of the hub genes

The miRNA targets of the hub genes were elucidated through exploring the CSmiRTar (http://cosbi4.ee.ncku.edu.tw/CSmiRTar/search) database [52], which compiles data from various established databases, including miRDB, TargetScan, microRNA.org, and DIANA microT. The miRNAs were filtered based on their experimental validation [54].

## Results

### Peak calling and gene annotation based on ChIP-seq data

**Fig. 1:**
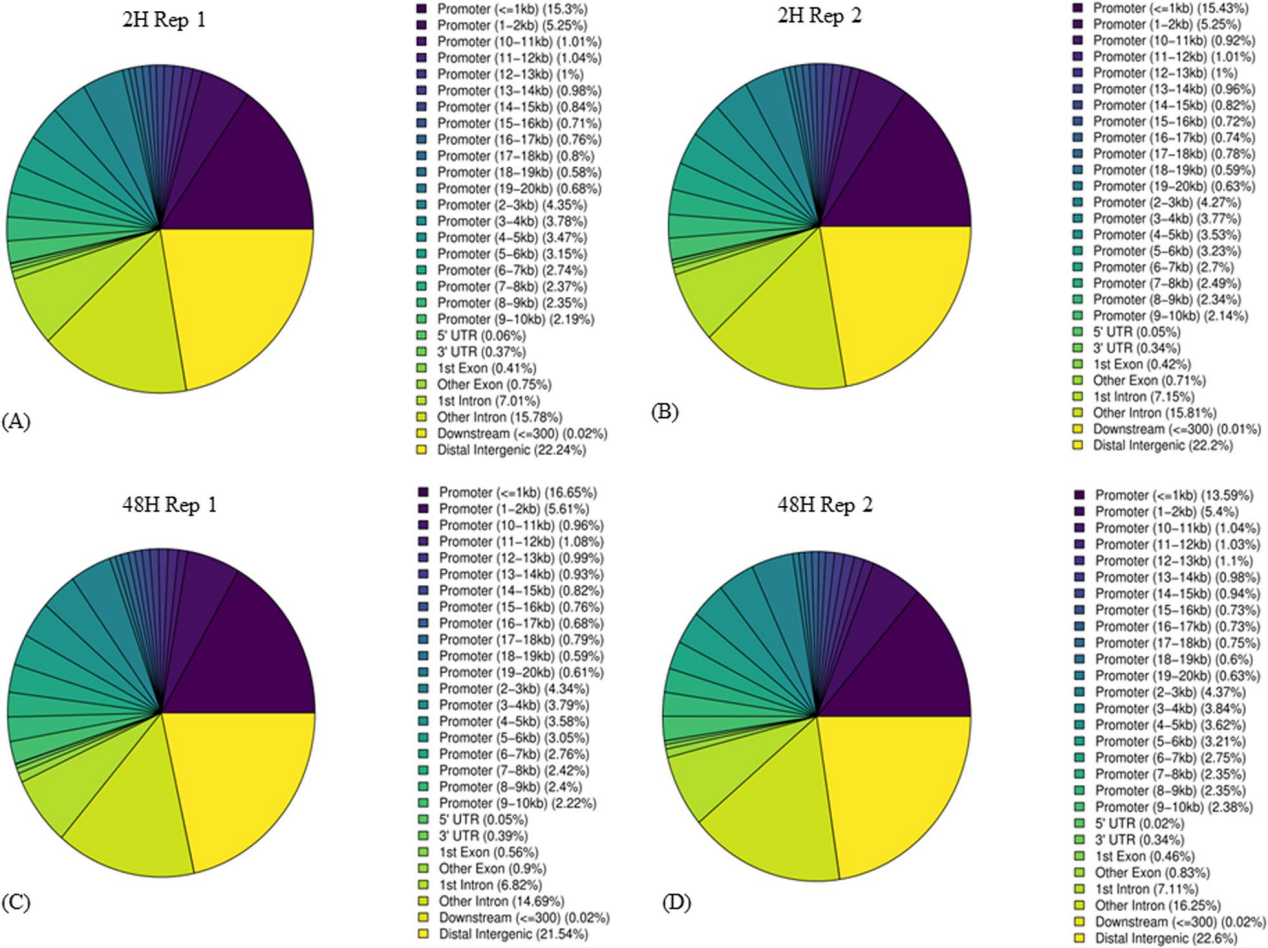
Peak distribution of all four ChIP-seq samples. 13.59–16.65% of peaks were located near the gene promoter (≤ 1 Kb), 21.54–22.24% of the peaks were located in the distal intergenic region, and 21.51–23.36% of the peaks were located in the intronic region

A total number of 33,853 peaks were obtained in sample ‘PPARG 2 h Rosiglitazone Rep1’, 27,476 peaks in sample ‘PPARG 2 h Rosiglitazone Rep2’, 27,124 peaks in ‘PPARG 48 h Rosiglitazone Rep1’, 14,854 peaks in ‘PPARG 48 h Rosiglitazone Rep2’. To visualize the overlap of the nonredundant peaks across different experimental conditions and replicates, we employed a Venn diagram analysis using the ‘limma’ package in R Studio (Supplementary Fig. 1) [53, 54]. While a total of 12,168 peaks are common in all four samples, 4992 peaks remain unique to ‘PPARG 48 h Rosiglitazone Rep1’, 311 peaks remain unique to ‘PPARG 48 h Rosiglitazone Rep2’, 6837 peaks remain unique to ‘PPARG 2 h Rosiglitazone Rep1’, and 2926 peaks remain unique to ‘PPARG 2 h Rosiglitazone Rep2’. Further annotated peak results showed that 13.59–16.65% of peaks were located near the gene promoters (≤ 1 Kb), and 21.54–22.24% of the peaks were located in the distal intergenic regions (Fig. 1). This suggests that the PPARG transcription factor is significantly involved in gene regulation.

### Differential gene expression analysis from the RNA-seq data

The raw gene counts from RNA-seq alignment were normalized (Supplementary Fig. 2A and B). Following normalization, the median values for each sample exhibited similarity across all conditions, indicating a uniformity in the technical quality of the data. The results of the principal component analysis (PCA) between multiple samples are shown in (Supplementary Fig. 2C), and the samples in the control (DMSO) and Rosiglitazone treatment groups were clustered separately.

**Fig. 2:**
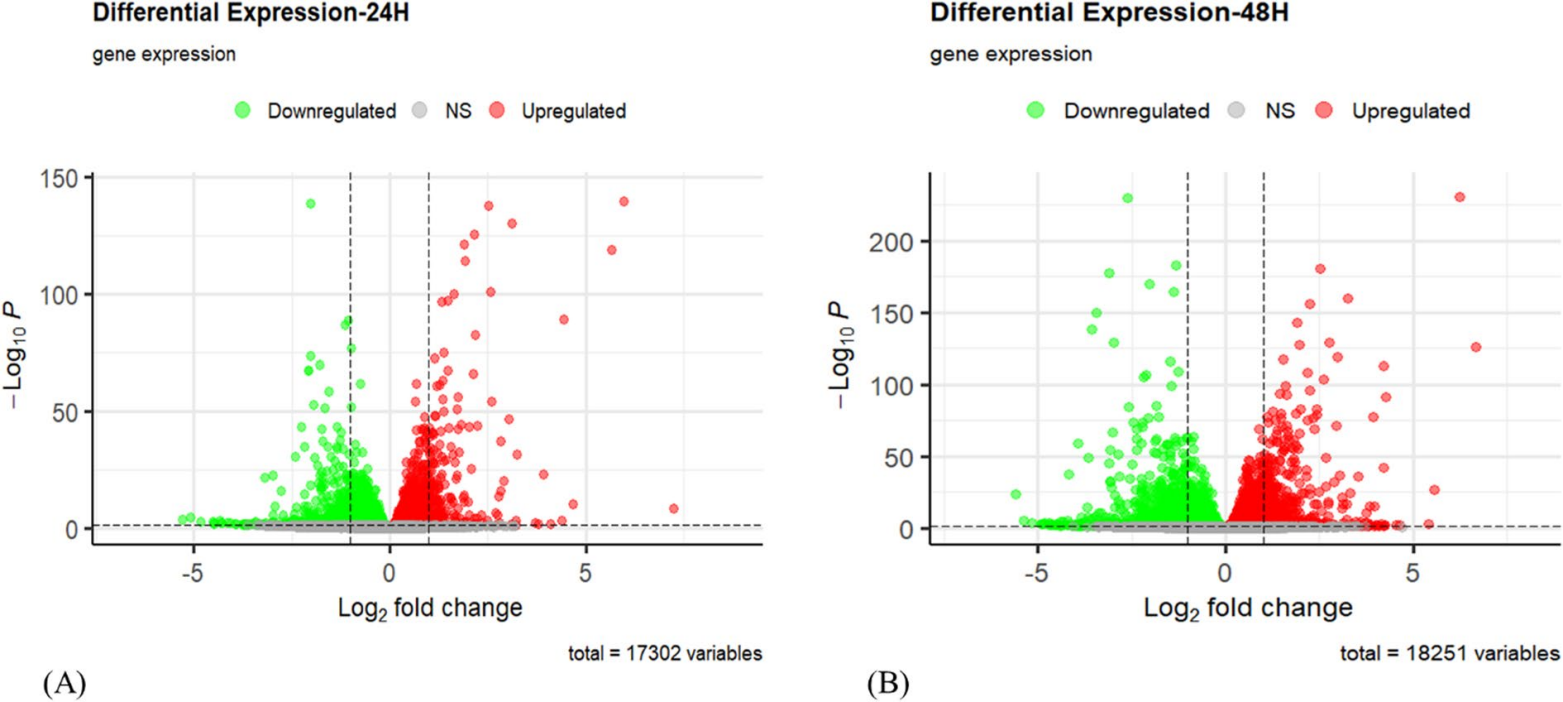
Volcano plot of the differentially expressed genes (DEGs) **A** at 24 h of Rosiglitazone-treated HT-29 cells. **B** 48 h of Rosiglitazone-treated HT-29 cells. (NS- non-significant)

**Fig. 3:**
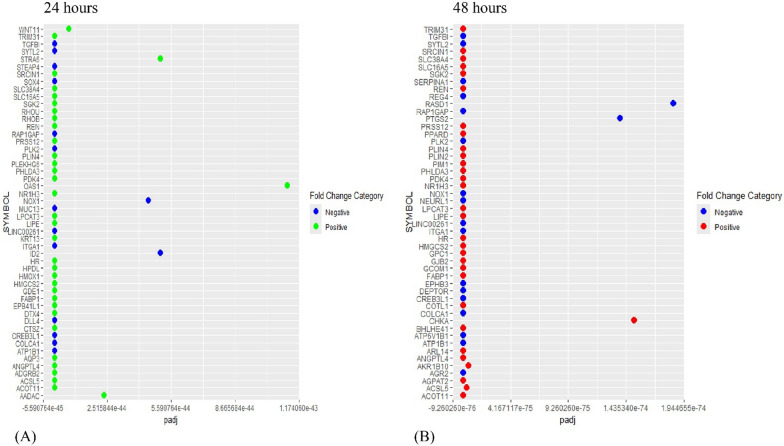
Top 50 differentially expressed genes (DEGs) (based on padj values) both in **A** 24 h and **B** 48 h Rosiglitazone treated cells

Following the differential expression analysis, 24 h of Rosiglitazone-treated HT-29 cells demonstrate 17,302 genes to be regulated. Out of which 1985 genes are downregulated (padj ≤ 0.05, log_2_FC < 0) (FC- Fold Change), and 2684 genes are upregulated (padj ≤ 0.05, log_2_FC > 0) with statistical significance (Fig. 2A). Similarly, 48 h of Rosiglitazone-treated HT-29 cells demonstrated 18,251 genes to be regulated. Out of which 3248 genes are downregulated (padj ≤ 0.05, log_2_FC < 0), and 3990 genes are upregulated (padj ≤ 0.05, log_2_FC > 0) with statistical significance (Fig. 2B). Furthermore top 50 DEGs (based on padj values) both in 24 h and 48 h Rosiglitazone treated cells were plotted in a heatmap using ‘ggplot2’ package (Fig. 3A and B) [36].

### Comparative analysis of ChIP-seq and RNA-seq data with identified PPRE-associated genes

The genes with regulatory PPRE regions denote 245, 237 245, and 184 overlapping genes amongst the annotated peaks in each ChIP-seq sample i.e. ‘PPARG 2 h Rosiglitazone Rep1’, ‘PPARG 2 h Rosiglitazone Rep2’, ‘PPARG 48 h Rosiglitazone Rep1’, ‘PPARG 48 h Rosiglitazone Rep2’ respectively. ‘jvenn’ webtool was employed to generate a Venn diagram of the overlapping genes (Supplementary Fig. 3A) [55]. Additionally, among the 29 PPRE-associated kinases, 15, 14, 15, and 13 kinase genes were identified as shared across the aforementioned samples (Table 2).

**Table 2:** List of kinases with regulatory PPRE regions identified across the annotated peaks of the PPARG-ChIP-seq samples

Serial number	2 h Rosiglitazone- Rep1	2 h Rosiglitazone- Rep2	48 h Rosiglitazone- Rep1	48 h Rosiglitazone- Rep2
1	BRSK2	BRSK2	BRSK2	BRSK2
2	CDK8	CDK8	CDK8	CDK8
3	FAM47E	FAM47E	FAM47E	FAM47E
4	KALRN	KALRN	KALRN	KALRN
5	MET	MET	MET	MET
6	PRKCH	PRKCH	PRKCH	PRKCH
7	TNK2	TNK2	TNK2	TNK2
8	MAGI2	MAGI2	MAGI2	MAGI2
9	MOB3C	MOB3C	MOB3C	PRKDC
10	PTK2	PTK2	PTK2	PTK2
11	SIK3	SIK3	SIK3	SIK3
12	STK24	STK24	STK24	STK24
13	CERK	CERK	CERK	CERK
14	PDK2		PDK2	
15	PRKCZ	PRKCZ	PRKCZ	

**Fig. 4:**
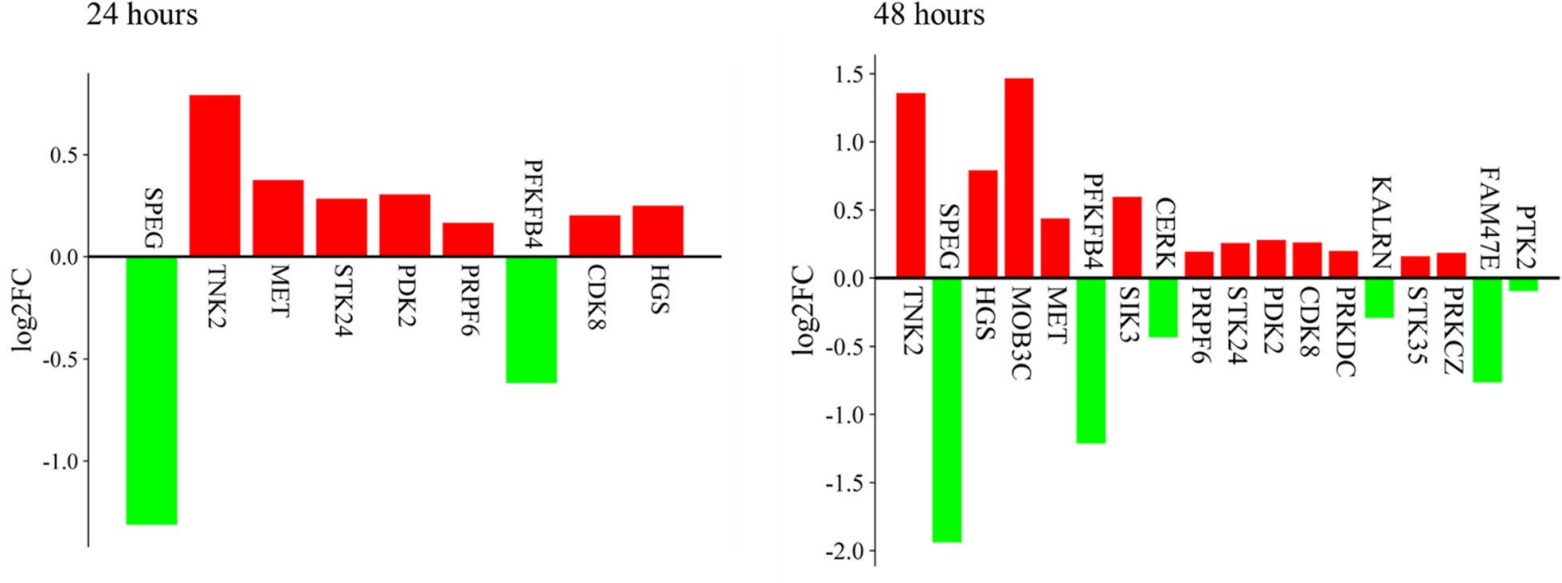
Bar graph for differential expression of the kinases, overlapping between PPRE-associated genes and the DEGs (P_adj_ ≤ 0.5) in the RNA-seq dataset

In 24 h Rosiglitazone and 48 h Rosiglitazone treated HT-29 cells 116 and 183 DEGs (padj ≤ 0.05) are overlapping with genes with regulatory PPRE regions respectively. ‘jvenn’ was again employed to generate a Venn diagram (Supplementary Fig. 3B). 26 out of 29 kinases show regulation in 24 h Rosiglitazone-treated HT-29 cells while 9 of them demonstrates statistical significance (padj ≤ 0.05) (Fig. 4) (Table 3). 27 out of 29 kinases show regulation in 48 h Rosiglitazone-treated HT-29 cells while 18 of them demonstrates statistical significance (padj ≤ 0.05) (Fig. 4) (Table 3). Also, a heatmap of the regulated kinases has been generated using SRplot (Supplementary Fig. 4) [37].

**Table 3:** Differential expression values of kinases with regulatory PPRE regions and the DEGS in the RNA-seq dataset (P_adj_ ≤ 0.05)

Treatment Hours	Gene	log_2_FoldChange	P_adj_
24 Hours	SPEG	− 1.312534637	1.59E-10
	TNK2	0.791229948	2.3E-10
	MET	0.375152835	2.52E-06
	STK24	0.283887967	8.7E-05
	PDK2	0.304869121	0.000185
	PRPF6	0.165477755	0.001507
	PFKFB4	− 0.618012886	0.002272
	CDK8	0.20208564	0.022362
	HGS	0.249448203	0.028574
48 Hours	TNK2	1.357409309	4.42E-30
	SPEG	− 1.939764685	2.86E-21
	HGS	0.790365865	2.45E-16
	MOB3C	1.465729199	5.58E-12
	MET	0.438277964	9.13E-09
	PFKFB4	− 1.211978	5.16E-08
	SIK3	0.59574155	3.67E-06
	CERK	− 0.432629411	3.87E-05
	PRPF6	0.192539018	9.39E-05
	STK24	0.256913107	0.00024
	PDK2	0.278442468	0.000516
	CDK8	0.260616434	0.001145
	PRKDC	0.198552603	0.00146
	KALRN	− 0.289148591	0.002779
	STK35	0.158934613	0.006635
	PRKCZ	0.183743567	0.015555
	FAM47E	− 0.763137943	0.018871
	PTK2	− 0.094567811	0.033603

### Gene ontology and pathway analysis of the 18 hits

**Fig. 5:**
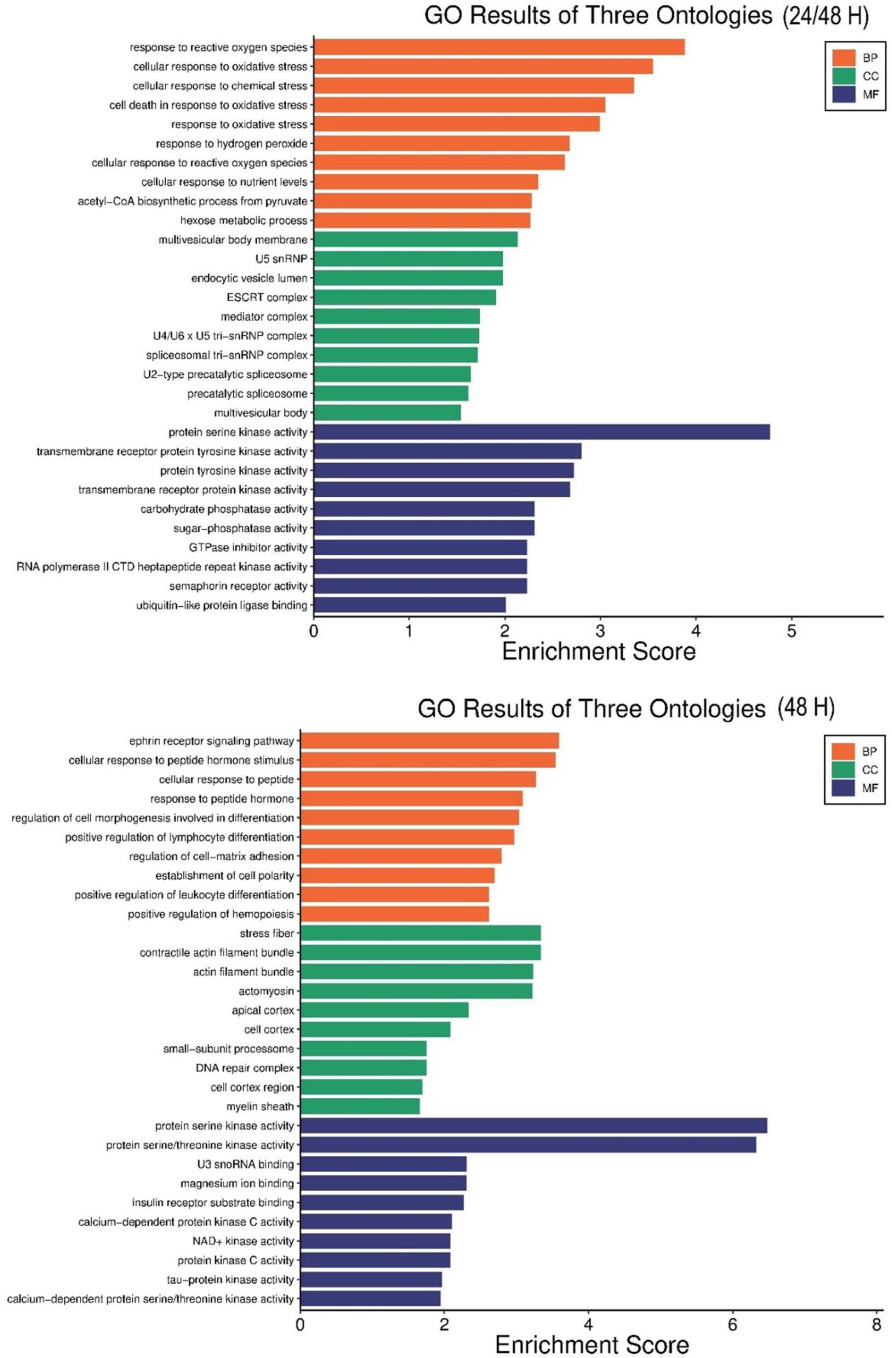
Gene Ontology (GO) enrichment of the DEGs at both 24 and 48 h, and 48 h only

SRplot was utilized for performing the GO analysis of the 18 significantly regulated kinases on a time point regulation basis. While the response to oxidative stress, nutrient levels, acetyl-CoA biosynthesis from pyruvate, hexose metabolism, GTPase inhibition, semaphorin receptor activity, and carbohydrate phosphates activity are the enriched GO terms for genes differentially regulating at both 24 and 48 h; enriched GO terms for genes differentially regulating at only 48 h are tau protein kinase activity, insulin receptor substrate binding, DNA repair complex formation, positive regulation of hemopoiesis, leukocyte differentiation, establishment of cell polarity and response to peptide hormones (Fig. 5).

**Fig. 6:**
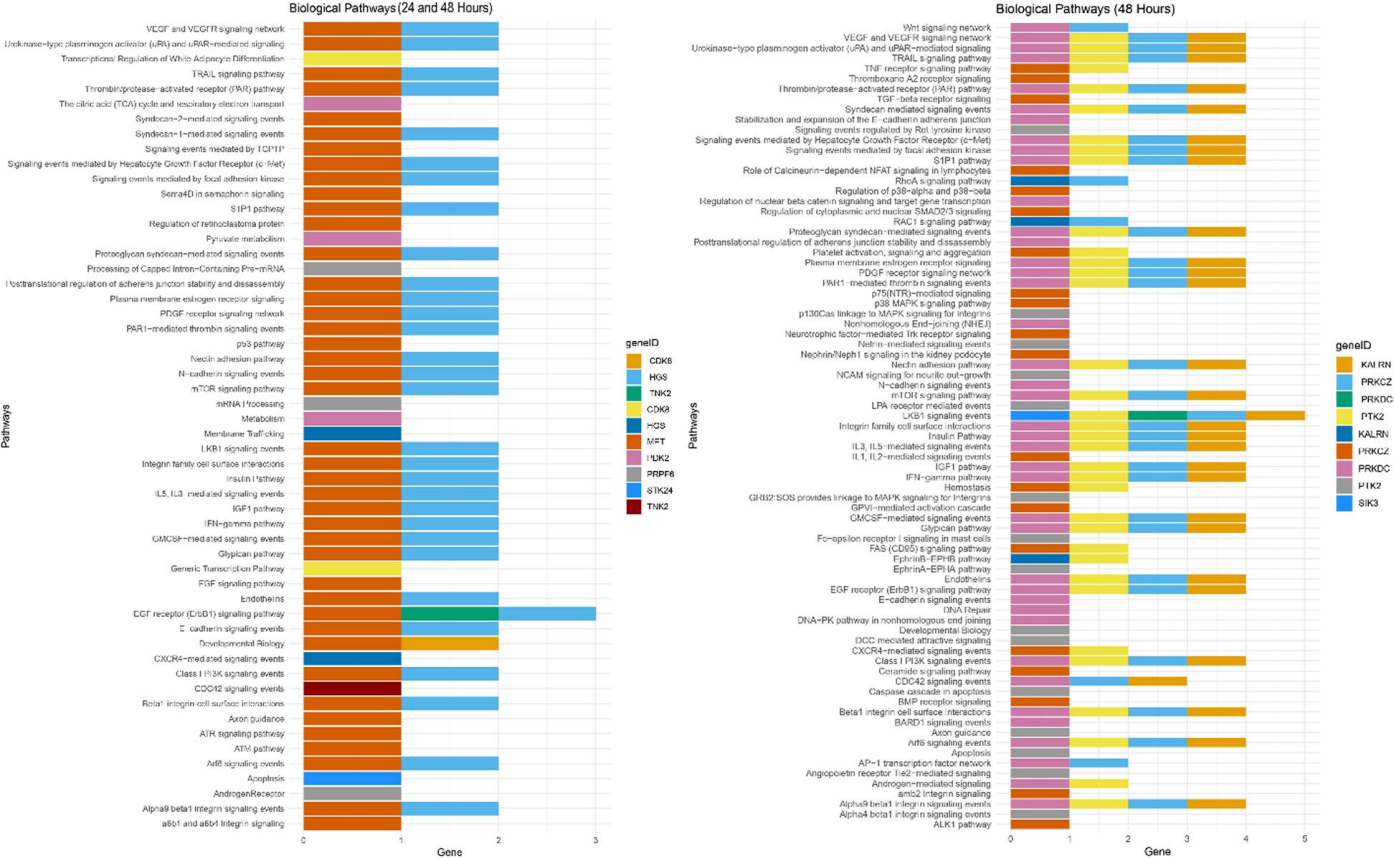
Pathway analysis of the DEGs at 24 and 48 h: The genes are substantially involved in the m-TOR signaling pathway, PDGF, GMCSF, IFN-gamma, IL-5, and IL-3 mediated signaling pathways

Further pathway analysis of the identified hits was conducted for genes regulated at both 24 and 48 h, and only at 48 h utilizing the FunRich database (12 of the 18 DEGs were annotated with pathways), and heatmaps were created using R Studio [38]. The genes are substantially involved in the m-TOR signaling pathway, PDGF, GMCSF, IFN-gamma, IL-5, and IL-3 mediated signaling pathways, and regulated by DEGs on both 24 and 48 h and 48 h only (Fig. 6). BMP, RhoA, TGF-beta, TNF, Wnt and ceramide signaling pathway, and DNA repair is only enriched at 48 h. TNK2, HGS, MET, PRKDC, KALRN, and PTK2 are mainly involved in PDGF, VEGF, S1P1, IL-3, 5, and m-TOR signaling pathways. They are also involved in Par1-mediated thrombin signal, integrin cell surface interactions, and insulin signaling events. Previously, through ChiP-seq analysis, it was revealed that PPARG binds to the promoter region of TNK2, MET, KALRN, PTK2, and in the intronic region of PRKDC.

### PPI network construction and Hub genes prediction

**Fig. 7:**
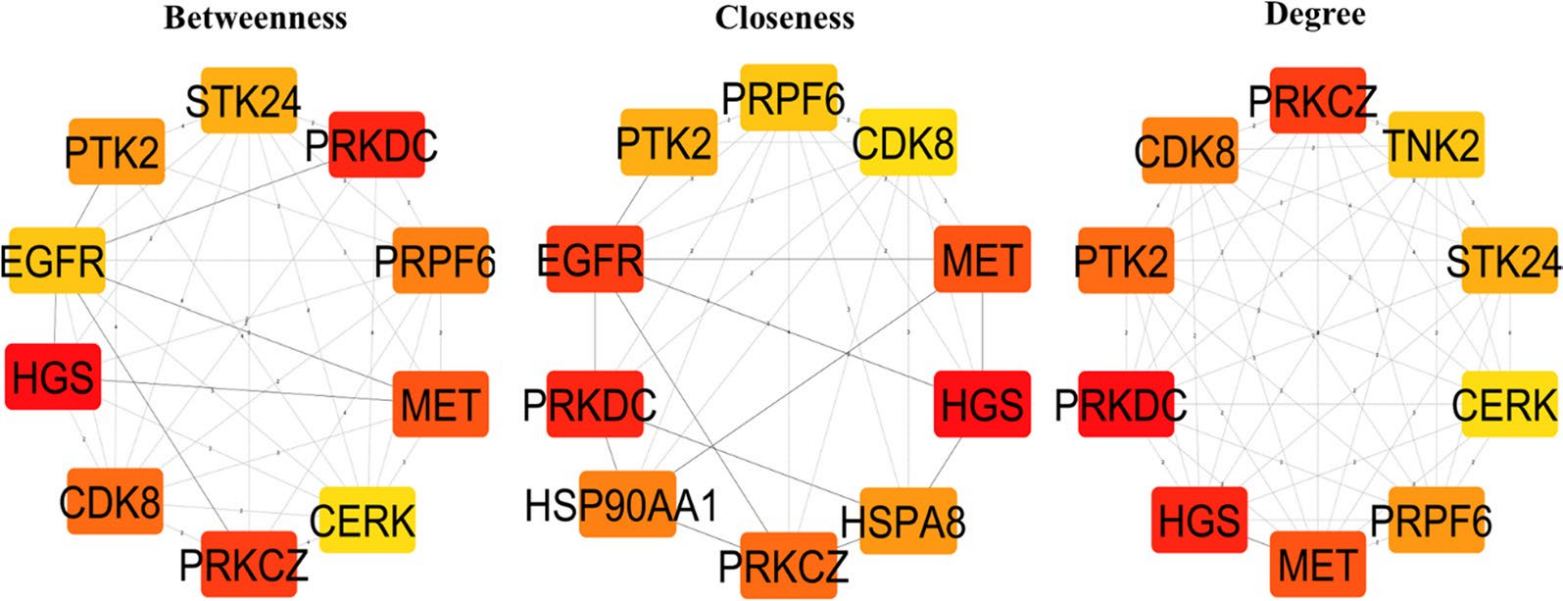
The cytoHubba interface integrated into the Cytoscape platform was utilized and the foremost 10 hub genes were outlined based on closeness, degree, and betweenness

The interactions exhibiting high confidence (confidence score ≥ 0.7) among the 18 DEGs with the HIPPIE-Human Scored Interactions PPI, were acquired from the NDEx platform (Supplementary Table 1) [39, 40]. The analysis indicated that out of the 19,485 nodes within the human PPI, the DEGs interact with 894 nodes, implying that approximately 4.36% of the PPI may be influenced by these 18 DEGs. The cytoHubba plugin within Cytoscape was employed to identify the hub genes within the network. The top 10 hub genes for closeness, degree, and betweenness were identified (Fig. 7) [41, 56]. 13 hub genes were discerned within the network (Supplementary Table 2) and seven (PRKDC, PRKCZ, MET, HGS, PTK2, PRPF6, CDK8) of which were present in all three parameters.

### Correlation of the hub genes with CRC

**Fig. 8:**
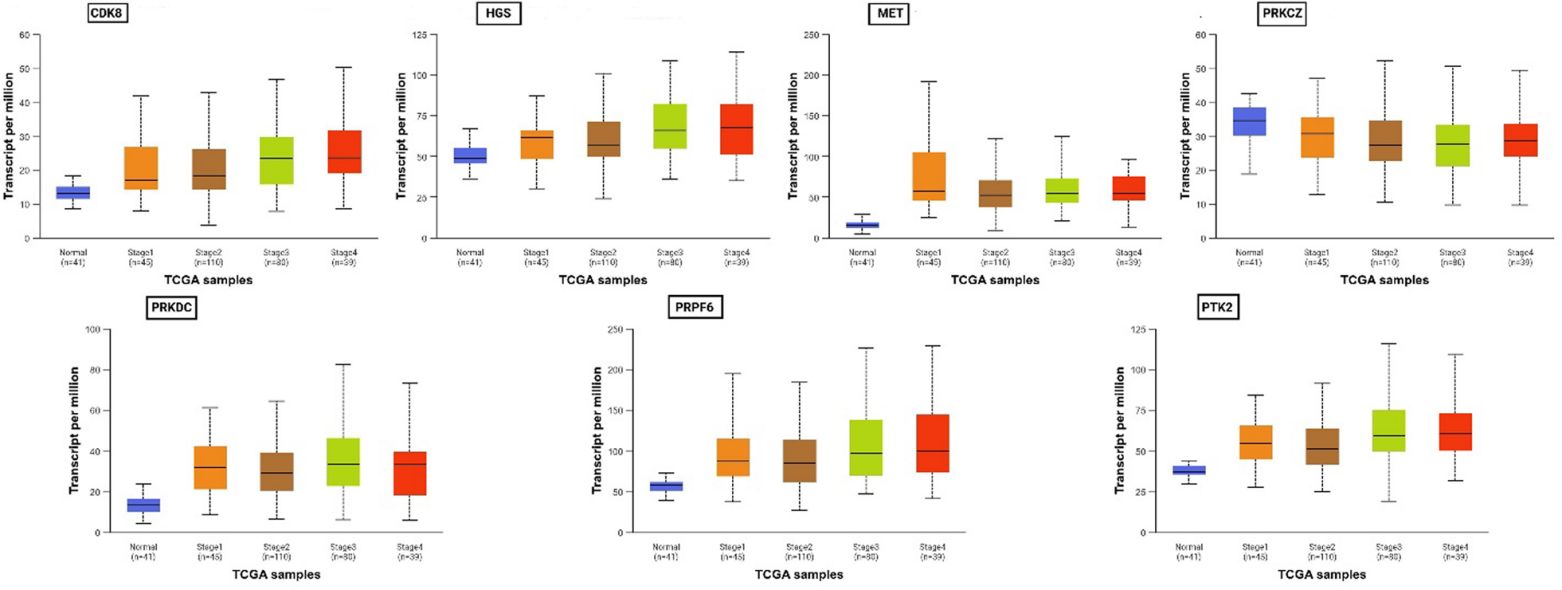
Box plots of relative expression level of the hub genes in normal and different cancer stage of colon adenocarcinoma (COAD)

The correlation of the hub genes with different stages of CRC was validated using UALCAN database. Elevated level of expression of CDK8, HGS, MET, PRKDC, PRPF6, and PTK2 is associated with different stages of CRC, whereas PRKCZ is expressed at low levels in CRC patients as compared to normal controls (Fig. 8). The gene effect scores derived from CRISPR knockout screens were also validated using the DeepMap source inside UALCAN. Negative scores imply cell growth inhibition and/or death following gene knockout (Supplementary Fig. 5). PRPF6 and HGS exhibit a unanimous negative score across all the CRC cell lines. CDK8, MET, PRKCZ, and PTK2 also exhibit negative scores against the majority of the cells, indicating inhibition of these hub genes could arrest cell proliferation required during cancer. PRKDC couldn’t be mapped using DeepMap in UALCAN.

**Fig. 9:**
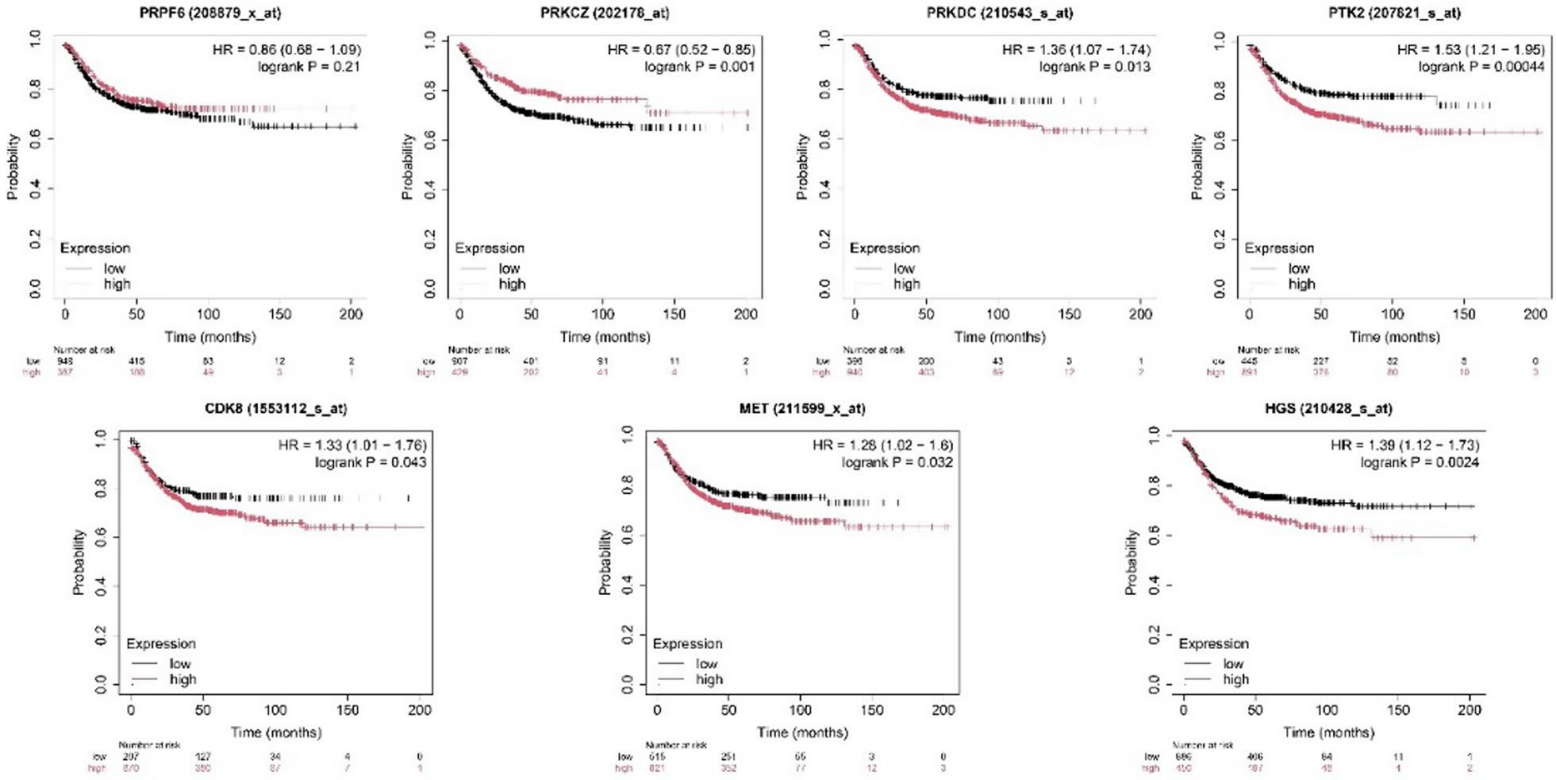
The impact of identified hub gene expression level on the overall survival of CRC patients: Low expression is indicated in black colour while red colour represents the high gene expression

The impact of identified hub gene expression level on the overall survival of CRC (n = 1336) patients was investigated using Kaplan–Meier survival analysis. High expression of PRKDC (HR = 1.37, logrank P = 0.013), PTK2 (HR = 1.53, logrank P = 0.00044), MET (HR = 1.28, logrank P = 0.032), HGS (HR = 1.39, logrank P = 0.0024), and CDK8 (HR = 1.33, logrank P = 0.043) is associated with lower survival probability. Low expression of PRPF6 (HR = 0.86, logrank P = 0.21) and PRKCZ (HR = 0.67, logrank P = 0.001) is lower survival probability (Fig. 9**)**. HR (Hazard Ratio) is a measure of the relative risk of an event (e.g., death) between the two groups. HR > 1 suggests a higher risk in the "high" expression group, and HR < 1 suggests a lower risk.

### Additional dataset analysis for validation

**Fig. 10:**
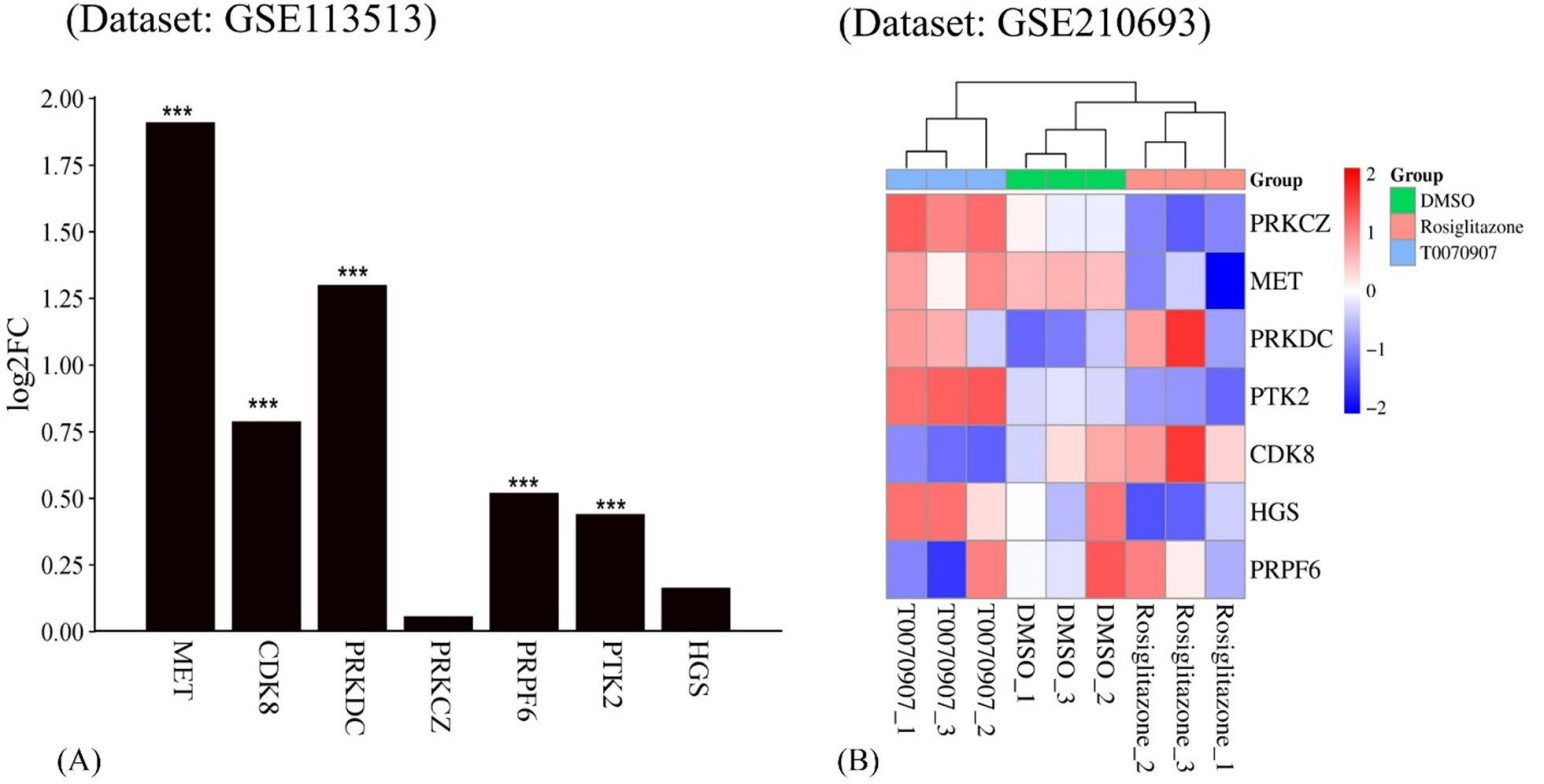
**(A)** The hub genes expression validated with microarray data containing samples from non-cancerous and CRC patients. All the hub genes expressed differential regulation in cancerous samples as compared to non-cancerous controls (*** represents padj ≤ 0.05). **(B)** On HT-1197 cells, T0070907, a PPARG antagonist, exhibits a reversal in the expression of the hub genes compared to the rosiglitazone treatment

The expression of the hub genes was also validated with additional microarray data containing samples from non-cancerous and CRC patients. All the hub genes expressed differential regulation in cancerous samples as compared to non-cancerous controls, and MET, CDK8, PRKDC, PRPF6, and PTK2 exhibit statistical significance (padj ≤ 0.05) (Fig. 10A). To further establish the transcriptional regulation of PPARG on the hub genes, gene counts from HT-1197 cells with PPARG antagonist T0070907 and agonist rosiglitazone and control (DMSO) were analyzed (Fig. 10B). T0070907 exhibits a reverse in the expression of the hub genes compared to the rosiglitazone treatment.

### Disease association of the hub genes

**Fig. 11:**
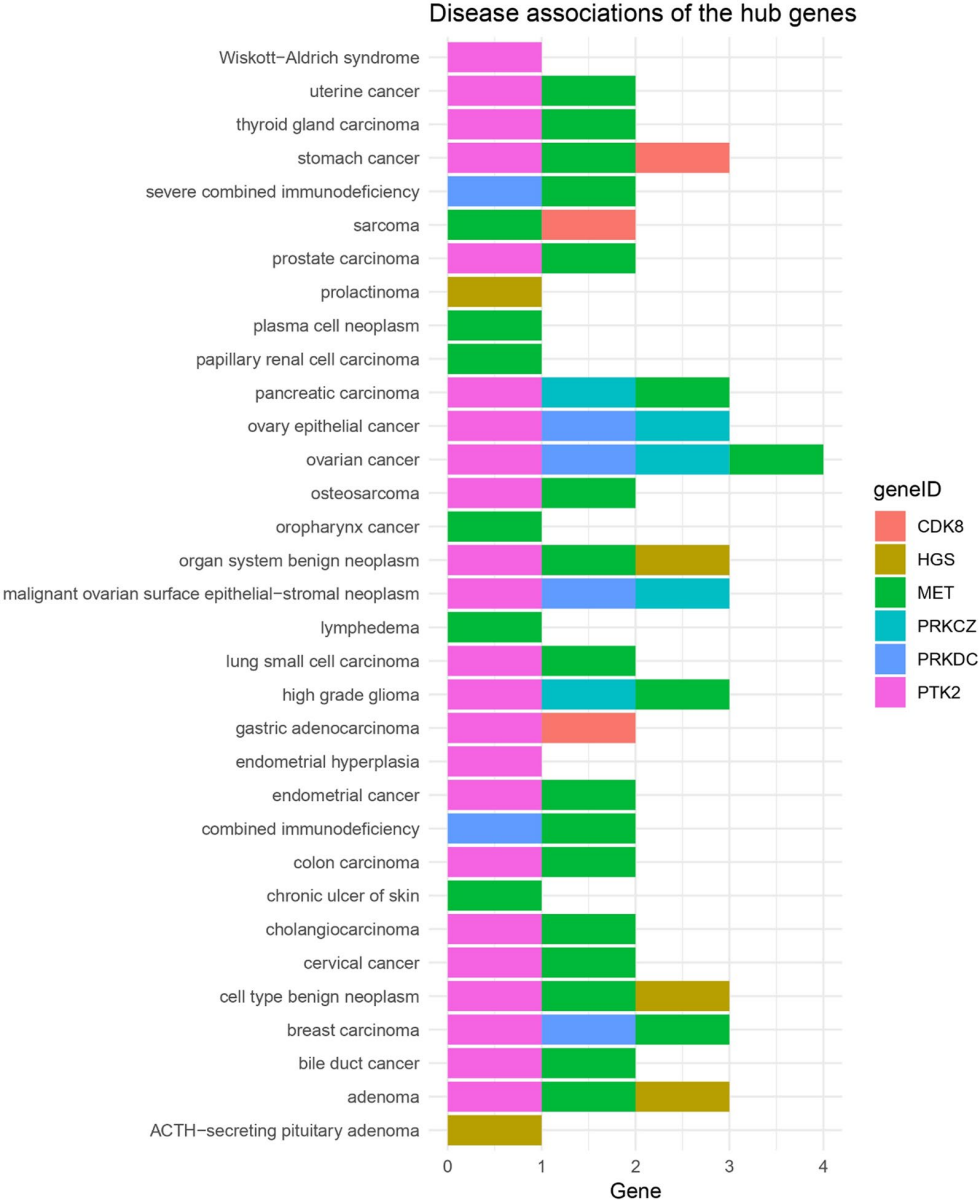
Heatmap of the hub genes and disease associations (*p* ≤ 0.05) was constructed using the DOSE package in R Studio. It was observed that the genes are involved in stomach, breast, lung, pancreas, prostate carcinoma, adenoma, glioma, severe combined immunodeficiency, Wiskott-Aldrich syndrome, and ACTH-secreting pituitary adenoma

The link between the hub genes and human diseases was explored and retrieved using the DOSE package in R studio, and a heatmap of the significant disease gene associations (*p* ≤ 0.05) was created (Fig. 11). It was observed that the genes are involved in stomach, breast, lung, pancreas, prostate carcinoma, adenoma, glioma, severe combined immunodeficiency, Wiskott-Aldrich syndrome, and ACTH-secreting pituitary adenoma. Mutational analysis of the hub genes using the UniProt database reveals that all the hub genes are associated with adenocarcinoma (Supplementary Table 3). A total of 174 pathogenic or likely pathogenic mutations in the hub genes with high impact have been elucidated in association with adenoma and adenocarcinoma, ductal lobular neoplasm, acute lymphoblastic leukemia, squamous cell neoplasms, glioma, plasma cell tumors, melanomas, and transitional cell papilloma (Supplementary Table 3).

### miRNA target prediction of the hub genes

CSmiRTar database reveals a total of 37 target experimentally validated miRNAs for six of the seven hub genes (Table 4). Within the CSmiRTar database, the experimentally validated targets are supported by miRTarBase.

**Table 4:** Target mi-RNAs of the hub genes. (ANS- Average Normalized Score; Not annotated: –)

Target Gene	miRNA	Normalized Score of				ANS	Supported databases
		miRNA from					
		DIANA-microT	miRanda.org	miRDB	Targetscan		
PTK2	hsa-miR-7-5p	0.429	0.306	0.557	0.281	0.393	4
	hsa-miR-138-5p	0.403	0.093	0.219	0.58	0.324	4
	hsa-miR-543	0.115	0.067	–	0	0.061	3
	hsa-miR-193a-3p	0.75	0.048	–	–	0.399	2
	hsa-let-7e-5p	0.201	0.281	–	–	0.241	2
	hsa-let-7c-5p	0.192	0.281	–	–	0.237	2
	hsa-miR-21-5p	0.283	0.071	–	–	0.177	2
PRKDC	hsa-miR-218-5p	–	0.088	–	0.267	0.178	2
	hsa-miR-101-5p	0.192	0.134	–	–	0.163	2
HGS	hsa-miR-142-3p	1	0.822	0.244	1	0.767	4
MET	hsa-miR-34c-5p	1	0.5	1	0.834	0.834	4
	hsa-miR-449b-5p	0.998	0.496	1	0.821	0.829	4
	hsa-miR-34a-5p	0.94	0.504	1	0.821	0.816	4
	hsa-miR-449a	0.938	0.496	1	0.821	0.814	4
	hsa-miR-130a-3p	0.996	0.438	0.269	0.597	0.575	4
	hsa-miR-23b-3p	0.949	0.513	0.019	0.291	0.443	4
	hsa-miR-34b-5p	1	0.424	0.543	–	0.656	3
	hsa-miR-206	0.804	0.561	–	0.365	0.577	3
	hsa-miR-1-3p	0.729	0.559	–	0.365	0.551	3
	hsa-miR-27a-3p	0.942	0.291	–	0.186	0.473	3
	hsa-miR-340-5p	0.889	0.265	–	0.029	0.394	3
	hsa-miR-31-5p	0.391	0.219	–	0.565	0.392	3
	hsa-miR-34b-3p	0.549	0.337	–	0.263	0.383	3
	hsa-miR-148a-3p	0.207	0.641	–	0.161	0.336	3
	hsa-miR-410-3p	0.311	0.479	–	0.103	0.298	3
	hsa-miR-144-3p	0.562	0.027	–	0	0.196	3
	hsa-miR-101-3p	0.055	0.43	–	0	0.162	3
	hsa-miR-198	–	0.264	–	0.541	0.403	2
	hsa-miR-409-3p	0.738	0.066	–	–	0.402	2
	hsa-miR-133b	–	0.005	–	0.108	0.057	2
	hsa-miR-562	0.22		–	–	0.22	1
	hsa-miR-30a-5p	–	0.136	–	–	0.136	1
	hsa-miR-199a-3p	–	0.029	–	–	0.029	1
	hsa-miR-137	–	0.016	–	–	0.016	1
	hsa-miR-1303	–	0.013	–	–	0.013	1
CDK8	hsa-miR-26a-5p	1	0.996	0.589	0.552	0.784	4
	hsa-miR-107	0.362	0.434	0.284	0.745	0.456	4

## Discussion

This study conducts an in-depth investigation into the involvement of PPARG in gene regulation by analyzing RNA-seq and ChIP-seq data in HT-29 cells treated with Rosiglitazone. The comprehensive mapping of PPARG binding sites and the identification of differentially expressed genes provide valuable insights into the transcriptional landscape controlled by PPARG in CRC. The observation that a considerable number of peaks are situated in proximity to gene promoters, intergenic regions, and intronic regions highlights the wide-ranging regulatory influence of PPARG throughout the human genome. The 18 DEGs associated with PPRE-linked kinases play a significant role in essential biological processes such as the response to peptide hormones, protein phosphorylation, tau protein kinase activity, insulin receptor substrate binding, acetyl-CoA biosynthesis from pyruvate, hexose metabolism, DNA repair complex formation, positive regulation of hemopoiesis, leukocyte differentiation, establishment of cell polarity and reactive oxygen species, while also showing enrichment in serine/threonine kinase activity and localization in specific cellular components like stress fibers and actin filament bundles. These results indicate that the activation of PPARG by Rosiglitazone not only governs metabolic pathways but also impacts cell structure and stress response mechanisms. Tau protein phosphorylation, specifically at Ser199/202, has been identified as a potential predictor of non-metastatic colon cancer [57].

The pyruvate dehydrogenase complex (PDC) in the mitochondria of colon cancer is largely responsible for converting pyruvate into acetyl-CoA, an essential substance for energy production and biosynthesis. Pyruvate is transported into the mitochondria, where it is converted to acetyl-CoA, NADH, and CO_2_ through oxidative decarboxylation [58, 59]. In colon cancer, hexose metabolism, particularly glucose, is significantly altered to support the rapid proliferation and survival of cancer cells [60]. DNA repair is essential for preserving genomic stability and averting cancer-causing mutations. One of the main contributing factors to the development and spread of colon cancer is the deregulation of various repair pathways [61]. In CRC, peptide hormones are essential in regulating tumor growth, invasion, angiogenesis, and metastasis, with several important peptide hormones such as ghrelin, neurotensin, parathyroid hormone-related peptide (PTHrP), and corticotropin-releasing hormone (CRH) being linked to various stages of CRC development; their abnormal expression can lead to tumor proliferation and unfavorable prognosis [62]. Irregular phosphorylation patterns frequently act as biomarkers for diagnosing and developing treatment plans; crucial pathways involved include the activation of kinases such as Src, EGFR, and PI3K, which results in the phosphorylation of specific tyrosine residues on target proteins, significantly influencing CRC progression [63–65]. The expression of serine-threonine kinase receptor-associated protein (STRAP) is heightened in CRCs and correlates with adverse outcomes [66]. Reactive oxygen species (ROS) play a pivotal role in triggering CRC and are involved in several processes such as epithelial-to-mesenchymal transition (EMT), angiogenesis, and apoptosis. A moderate rise in ROS can foster cancer development, while excessive amounts can induce cell death [67–69]. Stress fibers are contractile bundles of actin filaments that are vital for cell shape, migration, and mechano-transduction. In CRC, changes in the organization and localization of stress fibers have been linked to numerous facets of tumor advancement, including cell adhesion, invasion, and metastasis [70]. Focal adhesions are specialized structures where stress fibers connect to the extracellular matrix. In CRC, these focal adhesions are frequently dysregulated, resulting in enhanced cell motility and invasion [71–73]. Integrins are cell surface receptors that facilitate cell–matrix interactions and are critical for the formation and signaling of stress fibers. Abnormal integrin expression and signaling have been noted in CRC, contributing to tumor advancement [74].

The DEGs are also substantially involved in the m-TOR signaling pathway, PDGF, GMCSF, IFN-gamma, IL-5, and IL-3 mediated signaling pathways. The mTOR signaling pathway is essential in tumor development through its regulation of cell proliferation, metabolism, and metastasis, and through its hyperactivation within the PI3K/Akt/mTOR cascade. Elevated levels of activated mTOR (phosphorylated mTOR) are commonly found in CRC tissues, which correlate with advanced tumor stages characterized by the inhibition of apoptosis, heightened tumor aggressiveness, and diminished patient survival [75–77]. In CRC, the PDGF signaling pathway significantly contributes to tumor growth, invasion, angiogenesis, and metastasis by stimulating various downstream signaling cascades, primarily through the overproduction of PDGF ligands and their receptors (PDGFRs), particularly PDGFR-β, which results in increased cell proliferation, migration, and survival [78, 79]. The activation of PDGFR-β initiates pathways such as PI3K/Akt, MAPK (Ras/Raf/MEK/ERK), and the NOTCH pathway, thereby aiding tumor progression [80, 81]. Elevated levels of GM-CSF or its receptor in CRC often indicate a poor prognosis due to their involvement in tumor progression and immune evasion [82]. The GM-CSF signaling pathway primarily operates by activating the JAK2/STAT5 pathway, promoting tumor cell proliferation, survival, and immune suppression through the recruitment and activation of tumor-associated macrophages (TAMs) that typically display an M2-like phenotype; this process occurs through the binding of GM-CSF to its receptor on myeloid cells, activating downstream signaling cascades like the PI3K and MAPK pathways as well [83]. IL-5 is the main cytokine responsible for the differentiation and activation of eosinophils, while IL-3 also plays a role in eosinophil development, establishing them as crucial components of the tumor microenvironment when levels of these cytokines are heightened. Research indicates that increased eosinophil infiltration within CRC tumors, potentially influenced by IL-5 and IL-3, may be linked to a better prognosis, suggesting a possible anti-tumor effect of these cells [84, 85].

The recognition of seven hub genes (PRKDC, PRKCZ, MET, PTK2, CDK8, PRPF6, and HGS) in the protein–protein interaction network underscores their central role in mediating the effects of PPARG activation. These genes were additionally affirmed using gene impact scores from CRISPR screens and GEO datasets, demonstrating their differential expression in CRC samples and contribution to cancer cell survival. Furthermore, Kaplan–Meier plots revealed that high expression of five hub genes (MET, PRKDC, CDK8, HGS, PTK2) was associated with poor patient survival probability, whereas decreased expression of PRKCZ and PRPF6 was associated with lower survival probability. This establishes these genes as not only potential PPARG targets but also indicators for CRC prognosis. Transcriptional validation utilizing a second dataset (GSE210693) treated with PPARG antagonist T0070907 demonstrated that hub gene expression is reversed when compared to rosiglitazone therapy, reinforcing PPARG's regulatory role. Disease ontology and mutation analyses confirm the importance of these hub genes in a variety of malignancies, including stomach, lung, and prostate carcinomas. Furthermore, miRNA target prediction identified 37 experimentally verified miRNAs for six hub genes, indicating an additional regulatory layer that could be influenced by PPARG activity.

## Conclusion

This study offers valuable insights into the molecular and clinical implications of PPARG activation and its diverse implications in human health. The incorporation of various data types and comprehensive bioinformatics analyses establishes a sturdy framework for future investigations, potentially paving the way for novel therapeutic approaches targeting PPARG pathways.

## Supplementary Information

Additional file 1.

## Data Availability

The data supporting the findings of this study are available within the article and its supplementary materials or available on request from the authors. The European Nucleotide Archive (ENA) database was used to access the FASTQ files from the GSE77039 dataset (https://www.ebi.ac.uk/ena/browser/view/PRJNA309305). The NCBI Gene Expression Omnibus (GEO) was used to access the GSE113513 (https://www.ncbi.nlm.nih.gov/geo/query/acc.cgi?acc=GSE113513) and GSE210693 (https://www.ncbi.nlm.nih.gov/geo/query/acc.cgi?acc=GSE210693) datasets.

## Abbreviations

NR: Nuclear receptor
PPARS: Peroxisome proliferator-activated receptors
PPARG: PPAR-γ
CRC: Colorectal cancer
DEGs: Differentially expressed genes
RXR: Retinoid X receptors
PPRE: PPAR response element
PPI: Protein–protein interactome
ENA: European nucleotide archive
EBI: European bioinformatics institute
MACS2: Model-based analysis of ChIP-seq
P_adj_: Adjusted *P*-values
GO: Gene ontology
BP: Biological process
MF: Molecular function
CC: Cellular component
PCA: Principal component analysis
FC: Fold change
NS: Non-significant
PTHRP: Parathyroid hormone-related peptide
CRH: Corticotropin-releasing hormone
STRAP: Serine-threonine kinase receptor-associated protein
ROS: Reactive oxygen species
EMT: Epithelial-to-mesenchymal transition
TAMs: Tumor-associated macrophages
