# Supplementary material for: A multi-omics exploration of PPARG activation in colon cancer: kinases featuring a PPRE sequence within regulatory regions
Source: Biol Direct. 2025 Jun 11;20:68. doi: 10.1186/s13062-025-00654-7 (PMC12153098; doi:10.1186/s13062-025-00654-7)
Supplement: Supplementary file 1 — Additional file 1. [file 13062_2025_654_MOESM1_ESM.docx]

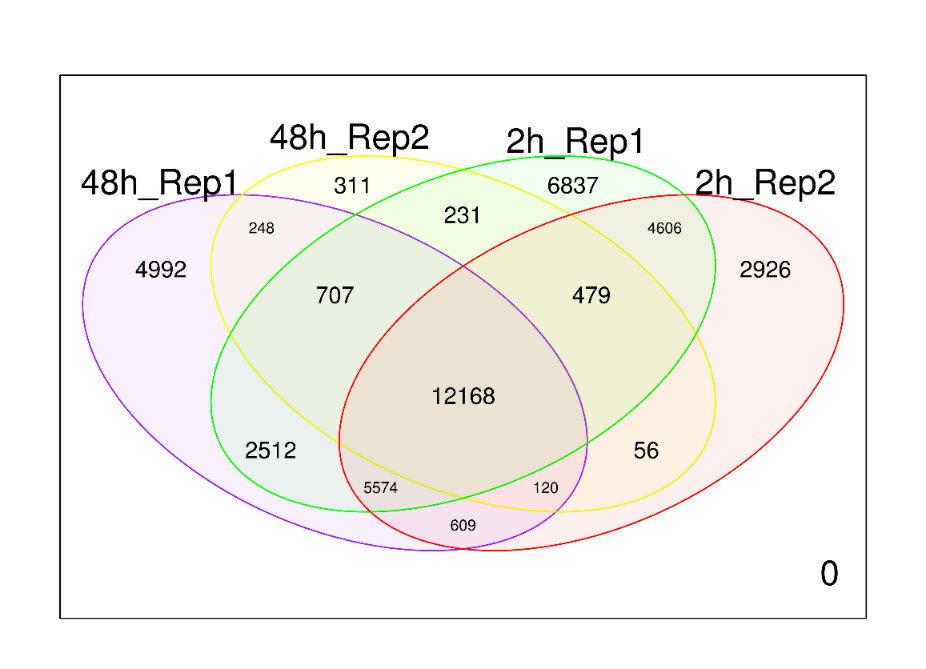


**Fig. 1:** Venn diagram of peaks in all four Chip-seq samples. While a total of 12168 peaks are common in all four samples, 4992 peaks remain unique to ‘PPARG 48h Rosiglitazone Rep1’, 311 peaks remain unique to ‘PPARG 48h Rosiglitazone Rep2’, 6837 peaks remain unique to ‘PPARG 2h Rosiglitazone Rep1’, and 2926 peaks remain unique to ‘PPARG 2h Rosiglitazone Rep2’.


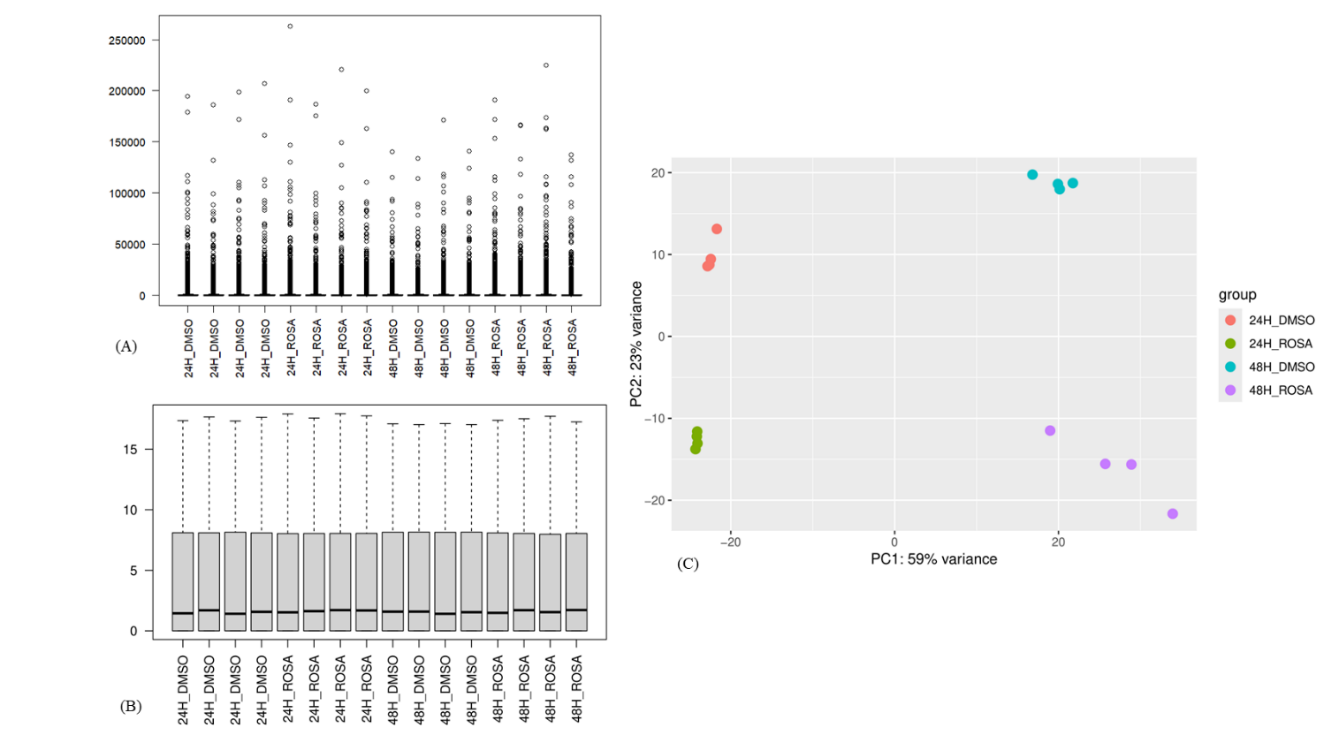


**Fig. 2:** Box plots of gene expression data **(A)** prior to normalization and **(B)** following normalization. **(C)** Principal component analysis (PCA) showed that the samples clustered into four groups.

**
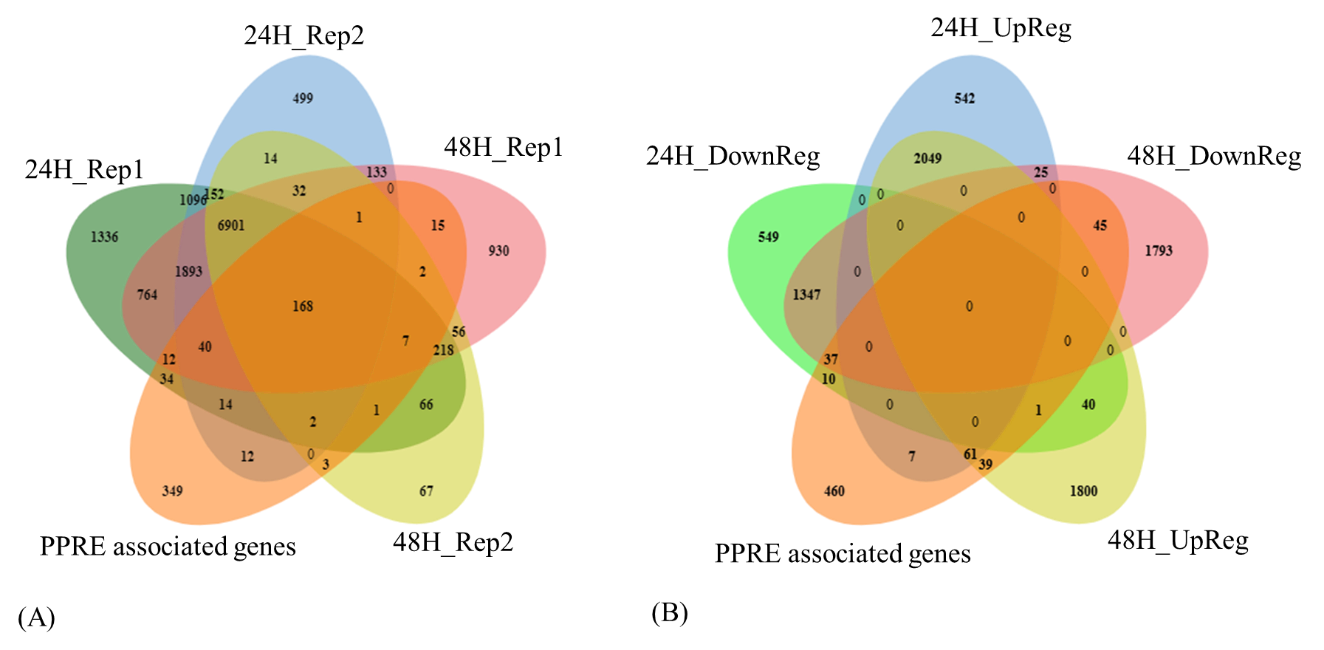
**

**Fig. 3:** Venn diagram representing the overlap between PPRE-associated genes and (A) the annotated Chip-Seq peaks. (B) DEGS in the RNA-Seq dataset (padj ≤ 0.05)


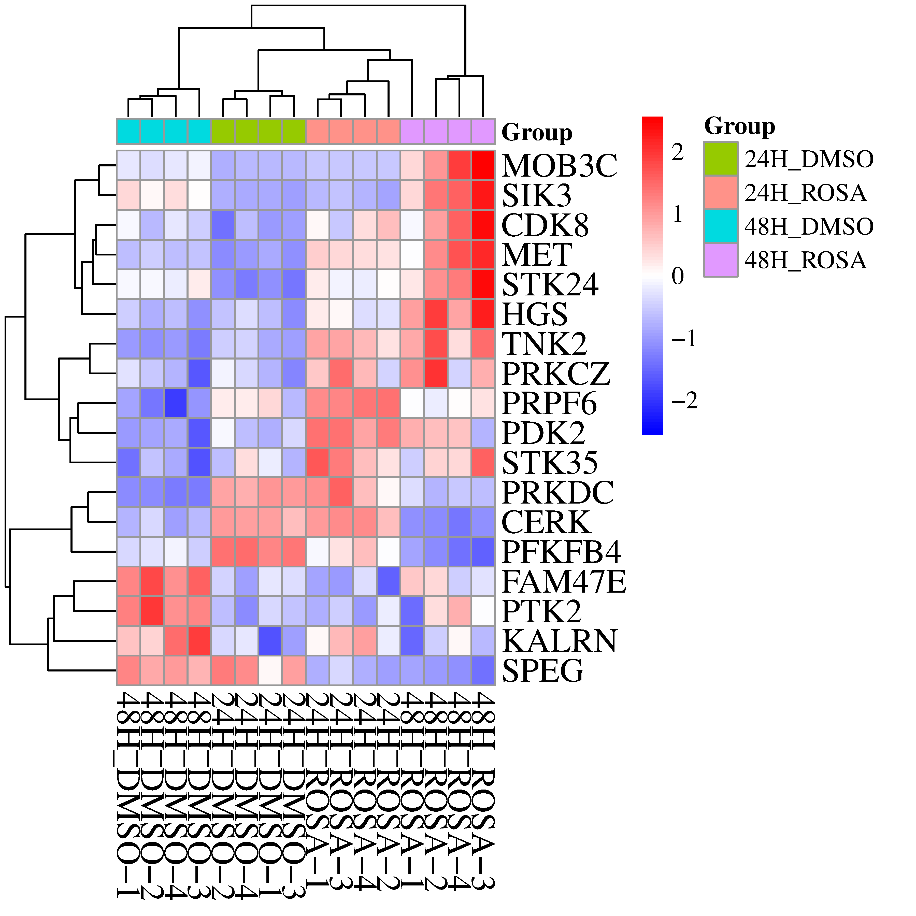


**Fig. 4**: Differential expression heatmap of the kinases, overlapping between PPRE-associated genes and the DEGs in the RNA-Seq dataset (padj ≤ 0.5).

**Fig. 5:** The gene effect scores derived from CRISPR knockout screens validated using the DeepMap source inside UALCAN. Negative scores imply cell growth inhibition and/or death following gene knockout.

**
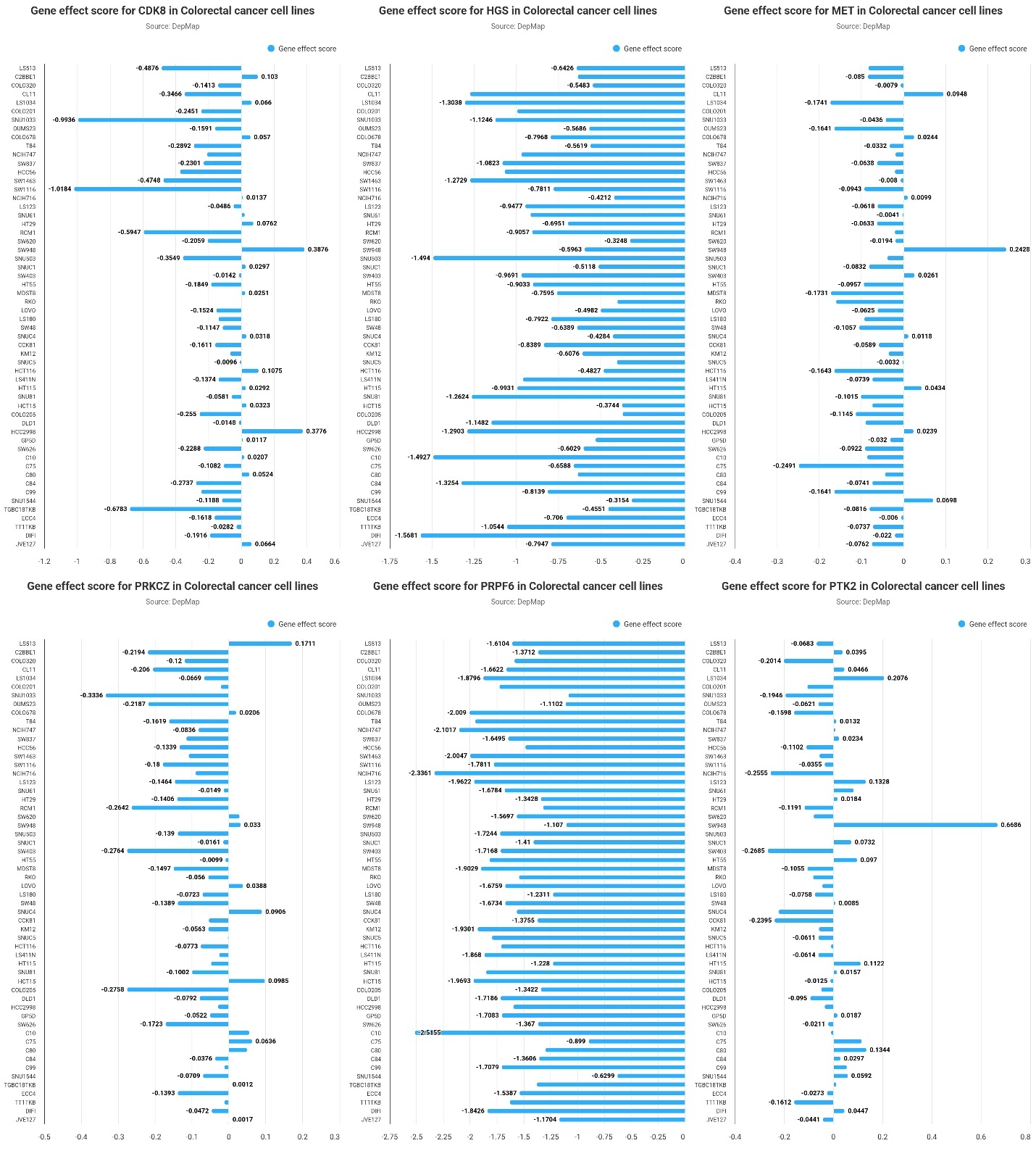
**

**Table 1:** High confidence interactions (confidence score ≥ 0.7) of the 18 DEGs with the human PPI**.**

| **Source Node** | **Target Node** | **Confidence Value** | **Interaction Detection Methods** |
| --- | --- | --- | --- |
| AAR2 | PRPF6 | 0.8 | MI:0004(affinity chromatography technology) |
| ACAD9 | PDK2 | 0.87 | MI:0004(affinity chromatography technology)\|MI:0007(anti tag coimmunoprecipitation)\|bioid |
| ADAP1 | PRKCZ | 0.89 | MI:0492(in vitro)\|Affinity Capture-Western\|Reconstituted Complex\|Biochemical Activity\|MI:0415(enzymatic study)\|MI:0004(affinity chromatography technology)\|MI:0096(pull down) |
| ADCK2 | PDK2 | 0.72 | MI:0004(affinity chromatography technology)\|MI:0007(anti tag coimmunoprecipitation) |
| ADRB2 | HGS | 0.75 | MI:0004(affinity chromatography technology)\|MI:0428(imaging technique) |
| AES | HGS | 0.87 | MI:0004(affinity chromatography technology)\|MI:0018(two hybrid)\|MI:0401(biochemical) |
| AGR2 | PRKDC | 0.72 | MI:0004(affinity chromatography technology)\|bioid |
| AKT1 | PRKDC | 0.88 | MI:0492(in vitro)\|MI:0415(enzymatic study)\|MI:0004(affinity chromatography technology)\|MI:0096(pull down) |
| ANXA2 | PTK2 | 0.72 | MI:0030(cross-linking study)\|bioid |
| ARAF | PRKDC | 0.73 | MI:0004(affinity chromatography technology) |
| ARHGAP26 | PTK2 | 0.76 | MI:0492(in vitro)\|MI:0493(in vivo)\|Reconstituted Complex\|MI:0096(pull down) |
| ARMC8 | HGS | 0.72 | MI:0004(affinity chromatography technology)\|MI:0018(two hybrid) |
| ARRB1 | HGS | 0.82 | MI:0004(affinity chromatography technology)\|MI:0007(anti tag coimmunoprecipitation) |
| ARRDC3 | HGS | 0.83 | MI:0416(fluorescence microscopy)\|MI:0007(anti tag coimmunoprecipitation)\|MI:0004(affinity chromatography technology) |
| ASAP1 | PTK2 | 0.96 | MI:0492(in vitro)\|MI:0493(in vivo)\|MI:0018(two hybrid)\|MI:0004(affinity chromatography technology)\|MI:0114(x-ray crystallography)\|bioid |
| ATG101 | PRKDC | 0.72 | MI:0007(anti tag coimmunoprecipitation)\|MI:0004(affinity chromatography technology) |
| ATG4C | PRKDC | 0.72 | MI:0007(anti tag coimmunoprecipitation)\|MI:0004(affinity chromatography technology) |
| ATM | PRKDC | 0.86 | MI:0492(in vitro)\|Protein-peptide\|Affinity Capture-Western\|MI:0004(affinity chromatography technology) |
| ATP6V1C2 | STK24 | 0.83 | MI:0004(affinity chromatography technology)\|MI:0007(anti tag coimmunoprecipitation) |
| AURKB | PRKDC | 0.78 | MI:0096(pull down)\|MI:0004(affinity chromatography technology) |
| AVPR2 | HGS | 0.75 | MI:0004(affinity chromatography technology)\|MI:0428(imaging technique) |
| BAD | PRKDC | 0.72 | MI:0007(anti tag coimmunoprecipitation)\|MI:0004(affinity chromatography technology) |
| BAG3 | TNK2 | 0.84 | MI:0096(pull down)\|MI:0004(affinity chromatography technology)\|MI:0007(anti tag coimmunoprecipitation) |
| BCAR3 | MET | 0.75 | MI:0053(fluorescence polarization spectroscopy)\|MI:0096(pull down) |
| BECN1 | PRKDC | 0.72 | MI:0007(anti tag coimmunoprecipitation)\|MI:0004(affinity chromatography technology) |
| BIN1 | PTK2 | 0.73 | MI:0492(in vitro)\|Affinity Capture-Western\|MI:0004(affinity chromatography technology) |
| BIRC5 | PRKDC | 0.73 | MI:0004(affinity chromatography technology) |
| BLK | MET | 0.75 | MI:0053(fluorescence polarization spectroscopy)\|MI:0096(pull down) |
| BRD4 | PRPF6 | 0.73 | MI:0004(affinity chromatography technology) |
| BRD4 | PRKDC | 0.73 | MI:0004(affinity chromatography technology) |
| BSG | PRPF6 | 0.83 | MI:0004(affinity chromatography technology)\|MI:0007(anti tag coimmunoprecipitation) |
| C1D | PRKDC | 0.79 | MI:0492(in vitro)\|MI:0018(two hybrid)\|Reconstituted Complex\|Biochemical Activity\|Affinity Capture-Western\|MI:0415(enzymatic study)\|MI:0004(affinity chromatography technology)\|MI:0096(pull down) |
| C1QBP | PRKCZ | 0.97 | MI:0492(in vitro)\|MI:0493(in vivo)\|Reconstituted Complex\|Affinity Capture-Western\|MI:0004(affinity chromatography technology)\|MI:0096(pull down)\|MI:0006(anti bait coimmunoprecipitation)\|MI:0007(anti tag coimmunoprecipitation) |
| C4orf19 | STK24 | 0.83 | MI:0004(affinity chromatography technology)\|MI:0007(anti tag coimmunoprecipitation) |
| CABCOCO1 | MOB3C | 0.73 | MI:0004(affinity chromatography technology) |
| CAMKK2 | PRKDC | 0.72 | MI:0007(anti tag coimmunoprecipitation)\|MI:0004(affinity chromatography technology) |
| CASP2 | PRKDC | 0.71 | MI:0027(cosedimentation)\|MI:0006(anti bait coimmunoprecipitation)\|MI:0096(pull down) |
| CBL | MET | 0.9 | MI:0492(in vitro)\|MI:0493(in vivo)\|MI:0018(two hybrid)\|Biochemical Activity\|Affinity Capture-Western\|Co-crystal Structure\|MI:0006(anti bait coimmunoprecipitation)\|MI:0019(coimmunoprecipitation)\|MI:0114(x-ray crystallography)\|MI:0045(experimental interaction detection)\|MI:0065(isothermal titration calorimetry)\|MI:0415(enzymatic study)\|MI:0004(affinity chromatography technology)\|MI:0096(pull down)\|MI:0107(surface plasmon resonance)\|MI:0069(mass spectrometry studies of complexes)\|MI:0047(far western blotting)\|MI:0428(imaging technique) |
| CBL | PTK2 | 0.76 | MI:0428(imaging technique)\|MI:0004(affinity chromatography technology) |
| CBLB | PTK2 | 0.73 | MI:0004(affinity chromatography technology) |
| CCNA1 | PTK2 | 0.75 | MI:0006(anti bait coimmunoprecipitation)\|MI:0428(imaging technique) |
| CCNDBP1 | CERK | 0.83 | MI:0004(affinity chromatography technology)\|MI:0007(anti tag coimmunoprecipitation) |
| CCR5 | PTK2 | 0.74 | MI:0492(in vitro)\|MI:0493(in vivo)\|Affinity Capture-Western\|MI:0004(affinity chromatography technology) |
| CD2BP2 | PRPF6 | 0.9 | Affinity Capture-MS\|MI:0006(anti bait coimmunoprecipitation)\|MI:0018(two hybrid)\|MI:0059(gst pull down)\|MI:0004(affinity chromatography technology)\|MI:0096(pull down)\|MI:0007(anti tag coimmunoprecipitation)\|MI:0676(tandem affinity purification)\|MI:0401(biochemical) |
| CD44 | MET | 0.84 | MI:0030(cross-linking study)\|MI:0055(fluorescent resonance energy transfer) |
| CD44 | PRKCZ | 0.82 | MI:0004(affinity chromatography technology)\|MI:0007(anti tag coimmunoprecipitation) |
| CD83 | CERK | 0.82 | MI:0004(affinity chromatography technology)\|MI:0007(anti tag coimmunoprecipitation) |
| CDC5L | PRKDC | 0.88 | Affinity Capture-MS\|MI:0025(copurification)\|MI:0004(affinity chromatography technology)\|MI:0006(anti bait coimmunoprecipitation)\|MI:0401(biochemical) |
| CDC5L | PRPF6 | 0.86 | MI:0006(anti bait coimmunoprecipitation)\|MI:0004(affinity chromatography technology)\|bioid |
| CDC5L | KALRN | 0.82 | MI:0018(two hybrid)\|MI:0399(two hybrid fragment pooling approach) |
| CDC5L | STK24 | 0.72 | MI:0006(anti bait coimmunoprecipitation)\|MI:0004(affinity chromatography technology) |
| CDH1 | MET | 0.72 | Affinity Capture-Western\|MI:0004(affinity chromatography technology) |
| CDK19 | CDK8 | 0.8 | MI:0226(ion exchange chromatography)\|MI:0004(affinity chromatography technology) |
| CDK2 | PRKDC | 0.73 | MI:0004(affinity chromatography technology) |
| CDK8 | CCNC | 1 | MI:0492(in vitro)\|MI:0493(in vivo)\|Affinity Capture-Western\|Co-fractionation\|Reconstituted Complex\|MI:0006(anti bait coimmunoprecipitation)\|MI:0096(pull down)\|MI:0424(protein kinase assay)\|MI:0004(affinity chromatography technology)\|MI:0055(fluorescent resonance energy transfer)\|MI:0114(x-ray crystallography)\|MI:0364(inferred by curator)\|MI:0676(tandem affinity purification)\|MI:0018(two hybrid)\|MI:0091(chromatography technology)\|MI:0071(molecular sieving)\|MI:0007(anti tag coimmunoprecipitation)\|MI:0012(bioluminescence resonance energy transfer)\|bioid\|MI:0401(biochemical) |
| CDK8 | MED7 | 0.96 | Affinity Capture-Western\|MI:0004(affinity chromatography technology)\|MI:0676(tandem affinity purification)\|MI:0096(pull down)\|MI:0007(anti tag coimmunoprecipitation)\|bioid |
| CDK8 | KAT2B | 0.93 | Affinity Capture-Western\|MI:0004(affinity chromatography technology)\|bioid |
| CDK8 | KAT2A | 0.91 | MI:0006(anti bait coimmunoprecipitation)\|MI:0226(ion exchange chromatography)\|bioid |
| CDK8 | MED12 | 0.9 | MI:0493(in vivo)\|Affinity Capture-MS\|Affinity Capture-Western\|MI:0004(affinity chromatography technology)\|MI:0676(tandem affinity purification)\|MI:0096(pull down)\|MI:0007(anti tag coimmunoprecipitation)\|MI:0006(anti bait coimmunoprecipitation)\|bioid\|MI:0401(biochemical) |
| CDK8 | MED23 | 0.9 | Affinity Capture-Western\|MI:0004(affinity chromatography technology)\|MI:0676(tandem affinity purification)\|MI:0007(anti tag coimmunoprecipitation)\|MI:0096(pull down)\|MI:0006(anti bait coimmunoprecipitation)\|bioid |
| CDK8 | MED14 | 0.9 | Affinity Capture-Western\|Affinity Capture-MS\|MI:0004(affinity chromatography technology)\|MI:0676(tandem affinity purification)\|MI:0007(anti tag coimmunoprecipitation)\|MI:0096(pull down)\|MI:0006(anti bait coimmunoprecipitation)\|bioid\|MI:0401(biochemical) |
| CDK8 | MED13 | 0.9 | Affinity Capture-MS\|Affinity Capture-Western\|MI:0004(affinity chromatography technology)\|MI:0676(tandem affinity purification)\|MI:0096(pull down)\|MI:0007(anti tag coimmunoprecipitation)\|MI:0006(anti bait coimmunoprecipitation)\|bioid |
| CDK8 | MED1 | 0.9 | Affinity Capture-MS\|Affinity Capture-Western\|MI:0004(affinity chromatography technology)\|MI:0226(ion exchange chromatography)\|MI:0006(anti bait coimmunoprecipitation)\|MI:0676(tandem affinity purification)\|MI:0007(anti tag coimmunoprecipitation)\|MI:0096(pull down)\|bioid\|MI:0401(biochemical) |
| CDK8 | MED24 | 0.9 | Affinity Capture-MS\|Affinity Capture-Western\|MI:0004(affinity chromatography technology)\|MI:0676(tandem affinity purification)\|MI:0007(anti tag coimmunoprecipitation)\|MI:0096(pull down)\|MI:0006(anti bait coimmunoprecipitation)\|bioid\|MI:0401(biochemical) |
| CDK8 | MED16 | 0.9 | Affinity Capture-MS\|Affinity Capture-Western\|MI:0004(affinity chromatography technology)\|MI:0676(tandem affinity purification)\|MI:0007(anti tag coimmunoprecipitation)\|MI:0096(pull down)\|MI:0006(anti bait coimmunoprecipitation)\|bioid |
| CDK8 | MED17 | 0.9 | Affinity Capture-MS\|Affinity Capture-Western\|MI:0004(affinity chromatography technology)\|MI:0676(tandem affinity purification)\|MI:0007(anti tag coimmunoprecipitation)\|MI:0096(pull down)\|MI:0006(anti bait coimmunoprecipitation)\|bioid |
| CDK8 | MED31 | 0.9 | Affinity Capture-MS\|MI:0004(affinity chromatography technology)\|MI:0676(tandem affinity purification)\|MI:0007(anti tag coimmunoprecipitation)\|MI:0096(pull down)\|bioid\|MI:0401(biochemical) |
| CDK8 | MED20 | 0.9 | Affinity Capture-Western\|MI:0004(affinity chromatography technology)\|MI:0676(tandem affinity purification)\|MI:0007(anti tag coimmunoprecipitation)\|MI:0096(pull down)\|bioid |
| CDK8 | MED15 | 0.9 | MI:0006(anti bait coimmunoprecipitation)\|MI:0004(affinity chromatography technology)\|MI:0226(ion exchange chromatography)\|MI:0676(tandem affinity purification)\|MI:0007(anti tag coimmunoprecipitation)\|MI:0096(pull down)\|bioid\|MI:0401(biochemical) |
| CDK8 | MED22 | 0.89 | MI:0004(affinity chromatography technology)\|MI:0676(tandem affinity purification)\|MI:0096(pull down)\|MI:0007(anti tag coimmunoprecipitation)\|bioid |
| CDK8 | MED13L | 0.89 | MI:0676(tandem affinity purification)\|MI:0004(affinity chromatography technology)\|MI:0007(anti tag coimmunoprecipitation)\|MI:0096(pull down)\|bioid |
| CDK8 | MED18 | 0.89 | MI:0676(tandem affinity purification)\|MI:0004(affinity chromatography technology)\|MI:0007(anti tag coimmunoprecipitation)\|MI:0096(pull down)\|bioid |
| CDK8 | MED4 | 0.89 | MI:0676(tandem affinity purification)\|MI:0004(affinity chromatography technology)\|MI:0007(anti tag coimmunoprecipitation)\|MI:0096(pull down)\|bioid |
| CDK8 | MED11 | 0.89 | MI:0676(tandem affinity purification)\|MI:0004(affinity chromatography technology)\|MI:0007(anti tag coimmunoprecipitation)\|MI:0096(pull down)\|bioid |
| CDK8 | MED27 | 0.89 | MI:0676(tandem affinity purification)\|MI:0004(affinity chromatography technology)\|MI:0007(anti tag coimmunoprecipitation)\|MI:0096(pull down)\|bioid |
| CDK8 | MED12L | 0.88 | MI:0004(affinity chromatography technology)\|MI:0096(pull down)\|MI:0007(anti tag coimmunoprecipitation)\|bioid |
| CDK8 | MTBP | 0.87 | MI:0004(affinity chromatography technology)\|MI:0007(anti tag coimmunoprecipitation)\|bioid |
| CDK8 | ZNF131 | 0.86 | MI:0004(affinity chromatography technology)\|MI:0007(anti tag coimmunoprecipitation)\|bioid |
| CDK8 | CDC37 | 0.84 | MI:0096(pull down)\|MI:0004(affinity chromatography technology)\|bioid |
| CDK8 | CTDP1 | 0.83 | MI:0415(enzymatic study)\|MI:0004(affinity chromatography technology)\|MI:0007(anti tag coimmunoprecipitation) |
| CDK8 | HSP90AA5P | 0.83 | MI:0004(affinity chromatography technology)\|MI:0007(anti tag coimmunoprecipitation) |
| CDK8 | PPP1R12A | 0.83 | MI:0004(affinity chromatography technology)\|MI:0007(anti tag coimmunoprecipitation) |
| CDK8 | CDK16 | 0.83 | MI:0004(affinity chromatography technology)\|MI:0007(anti tag coimmunoprecipitation) |
| CDK8 | POLR2C | 0.82 | MI:0004(affinity chromatography technology)\|MI:0007(anti tag coimmunoprecipitation) |
| CDK8 | CDK9 | 0.78 | MI:0096(pull down)\|MI:0006(anti bait coimmunoprecipitation) |
| CDK8 | SMARCB1 | 0.77 | Affinity Capture-Western\|Co-fractionation\|MI:0004(affinity chromatography technology)\|MI:0401(biochemical) |
| CDK8 | SMAD1 | 0.73 | MI:0007(anti tag coimmunoprecipitation)\|MI:0004(affinity chromatography technology)\|MI:0415(enzymatic study) |
| CDK8 | TP53BP1 | 0.73 | Bioid |
| CDK8 | CEBPB | 0.72 | Affinity Capture-Western\|MI:0004(affinity chromatography technology) |
| CDK8 | THRAP3 | 0.72 | Affinity Capture-MS\|MI:0004(affinity chromatography technology) |
| CDK8 | HNRNPF | 0.72 | MI:0676(tandem affinity purification)\|MI:0004(affinity chromatography technology) |
| CDK8 | HERC2 | 0.72 | MI:0676(tandem affinity purification)\|MI:0004(affinity chromatography technology) |
| CDK8 | CELF1 | 0.72 | MI:0004(affinity chromatography technology)\|MI:0007(anti tag coimmunoprecipitation) |
| CDK8 | SRGAP1 | 0.72 | MI:0004(affinity chromatography technology)\|MI:0007(anti tag coimmunoprecipitation) |
| CDK8 | GEMIN5 | 0.72 | MI:0004(affinity chromatography technology)\|MI:0007(anti tag coimmunoprecipitation) |
| CDK8 | WWP1 | 0.72 | MI:0004(affinity chromatography technology)\|MI:0007(anti tag coimmunoprecipitation) |
| CDK8 | BUD13 | 0.72 | MI:0004(affinity chromatography technology)\|MI:0007(anti tag coimmunoprecipitation) |
| CDK8 | DENND4C | 0.72 | MI:0004(affinity chromatography technology)\|MI:0007(anti tag coimmunoprecipitation) |
| CDK8 | RBM4 | 0.72 | MI:0004(affinity chromatography technology)\|MI:0007(anti tag coimmunoprecipitation) |
| CDK8 | UBL4A | 0.72 | MI:0004(affinity chromatography technology)\|MI:0007(anti tag coimmunoprecipitation) |
| CDK8 | BAG6 | 0.72 | MI:0004(affinity chromatography technology)\|MI:0007(anti tag coimmunoprecipitation) |
| CDK8 | TAF1 | 0.72 | MI:0004(affinity chromatography technology)\|MI:0007(anti tag coimmunoprecipitation) |
| CDK8 | TBX3 | 0.72 | MI:0004(affinity chromatography technology)\|MI:0007(anti tag coimmunoprecipitation) |
| CDK8 | GCFC2 | 0.72 | MI:0004(affinity chromatography technology)\|MI:0007(anti tag coimmunoprecipitation) |
| CDK8 | PCF11 | 0.72 | MI:0004(affinity chromatography technology)\|MI:0007(anti tag coimmunoprecipitation) |
| CDK8 | GET4 | 0.72 | MI:0004(affinity chromatography technology)\|MI:0007(anti tag coimmunoprecipitation) |
| CDK8 | SUPT7L | 0.72 | MI:0004(affinity chromatography technology)\|MI:0007(anti tag coimmunoprecipitation) |
| CDK8 | POLR1D | 0.72 | MI:0004(affinity chromatography technology)\|MI:0007(anti tag coimmunoprecipitation) |
| CDK8 | RBM45 | 0.72 | MI:0004(affinity chromatography technology)\|MI:0007(anti tag coimmunoprecipitation) |
| CDK8 | CCNT1 | 0.7 | MI:0006(anti bait coimmunoprecipitation) |
| CDK8 | KPNA1 | 0.7 | bioid |
| CDK9 | PRKDC | 0.74 | MI:0004(affinity chromatography technology) |
| CEACAM21 | MET | 0.83 | MI:0004(affinity chromatography technology)\|MI:0007(anti tag coimmunoprecipitation) |
| CENPA | PRKDC | 0.72 | MI:0004(affinity chromatography technology)\|MI:0676(tandem affinity purification) |
| CERK | NOTCH2NLA | 0.73 | MI:0018(two hybrid) |
| CERK | U2AF2 | 0.72 | MI:0004(affinity chromatography technology)\|MI:0007(anti tag coimmunoprecipitation) |
| CERK | CELF1 | 0.72 | MI:0004(affinity chromatography technology)\|MI:0007(anti tag coimmunoprecipitation) |
| CERK | TBC1D22A | 0.72 | MI:0004(affinity chromatography technology)\|MI:0007(anti tag coimmunoprecipitation) |
| CERK | CCDC174 | 0.72 | MI:0004(affinity chromatography technology)\|MI:0007(anti tag coimmunoprecipitation) |
| CERK | APOL2 | 0.72 | MI:0004(affinity chromatography technology)\|MI:0007(anti tag coimmunoprecipitation) |
| CERK | WDR43 | 0.72 | MI:0004(affinity chromatography technology)\|MI:0007(anti tag coimmunoprecipitation) |
| CERK | USP1 | 0.72 | MI:0004(affinity chromatography technology)\|MI:0007(anti tag coimmunoprecipitation) |
| CERK | BAG6 | 0.72 | MI:0004(affinity chromatography technology)\|MI:0007(anti tag coimmunoprecipitation) |
| CERK | TAF13 | 0.72 | MI:0004(affinity chromatography technology)\|MI:0007(anti tag coimmunoprecipitation) |
| CERK | UTP4 | 0.72 | MI:0004(affinity chromatography technology)\|MI:0007(anti tag coimmunoprecipitation) |
| CERK | PRRC2B | 0.72 | MI:0004(affinity chromatography technology)\|MI:0007(anti tag coimmunoprecipitation) |
| CERK | ILKAP | 0.72 | MI:0004(affinity chromatography technology)\|MI:0007(anti tag coimmunoprecipitation) |
| CERK | HLTF | 0.72 | MI:0004(affinity chromatography technology)\|MI:0007(anti tag coimmunoprecipitation) |
| CERK | SPG7 | 0.72 | MI:0004(affinity chromatography technology)\|MI:0007(anti tag coimmunoprecipitation) |
| CERK | DDX23 | 0.72 | MI:0004(affinity chromatography technology)\|MI:0007(anti tag coimmunoprecipitation) |
| CERK | PRPF4 | 0.72 | MI:0004(affinity chromatography technology)\|MI:0007(anti tag coimmunoprecipitation) |
| CERK | UBL4A | 0.72 | MI:0004(affinity chromatography technology)\|MI:0007(anti tag coimmunoprecipitation) |
| CERK | GEMIN2 | 0.72 | MI:0004(affinity chromatography technology)\|MI:0007(anti tag coimmunoprecipitation) |
| CERK | IK | 0.72 | MI:0004(affinity chromatography technology)\|MI:0007(anti tag coimmunoprecipitation) |
| CERK | PRMT1 | 0.72 | MI:0004(affinity chromatography technology)\|MI:0007(anti tag coimmunoprecipitation) |
| CERK | NR3C1 | 0.72 | MI:0004(affinity chromatography technology)\|MI:0007(anti tag coimmunoprecipitation) |
| CERK | ALDH18A1 | 0.72 | MI:0004(affinity chromatography technology)\|MI:0007(anti tag coimmunoprecipitation) |
| CERK | RAD18 | 0.72 | MI:0004(affinity chromatography technology)\|MI:0007(anti tag coimmunoprecipitation) |
| CERK | NCBP1 | 0.72 | MI:0004(affinity chromatography technology)\|MI:0007(anti tag coimmunoprecipitation) |
| CERK | BUB1B | 0.72 | MI:0004(affinity chromatography technology)\|MI:0007(anti tag coimmunoprecipitation) |
| CERK | EIF4ENIF1 | 0.72 | MI:0004(affinity chromatography technology)\|MI:0007(anti tag coimmunoprecipitation) |
| CERK | RBM26 | 0.72 | MI:0004(affinity chromatography technology)\|MI:0007(anti tag coimmunoprecipitation) |
| CFTR | PRKDC | 0.85 | MI:0006(anti bait coimmunoprecipitation)\|MI:0004(affinity chromatography technology)\|MI:0096(pull down) |
| CHD1L | PRKDC | 0.82 | MI:0004(affinity chromatography technology)\|MI:0007(anti tag coimmunoprecipitation) |
| CIB1 | PTK2 | 0.73 | MI:0493(in vivo)\|Affinity Capture-Western\|MI:0004(affinity chromatography technology) |
| CLK2 | PRPF6 | 0.78 | MI:0096(pull down)\|MI:0018(two hybrid) |
| CLN3 | PRKDC | 0.82 | MI:0007(anti tag coimmunoprecipitation)\|MI:0004(affinity chromatography technology) |
| CLTC | HGS | 0.96 | MI:0492(in vitro)\|MI:0018(two hybrid)\|Reconstituted Complex\|MI:0004(affinity chromatography technology)\|MI:0096(pull down) |
| COPS5 | PRKDC | 0.72 | MI:0004(affinity chromatography technology)\|MI:0676(tandem affinity purification) |
| CREBBP | CDK8 | 0.77 | Co-fractionation\|Affinity Capture-Western\|MI:0004(affinity chromatography technology)\|MI:0401(biochemical) |
| CRK | PTK2 | 0.9 | MI:0492(in vitro)\|MI:0493(in vivo)\|Affinity Capture-Western\|Reconstituted Complex\|MI:0004(affinity chromatography technology)\|MI:0096(pull down)\|MI:0006(anti bait coimmunoprecipitation)\|MI:0018(two hybrid) |
| CSNK1A1 | PRKDC | 0.73 | MI:0004(affinity chromatography technology) |
| CSTF2 | HGS | 0.73 | MI:0018(two hybrid) |
| CTNNB1 | MET | 0.83 | MI:0493(in vivo)\|Affinity Capture-Western\|MI:0004(affinity chromatography technology) |
| CTNNB1 | PRPF6 | 0.73 | MI:0004(affinity chromatography technology) |
| CTSB | PRKDC | 0.72 | MI:0004(affinity chromatography technology)\|bioid |
| CTSS | PRKDC | 0.72 | MI:0004(affinity chromatography technology)\|bioid |
| CTTNBP2NL | STK24 | 0.9 | Affinity Capture-MS\|Affinity Capture-Western\|MI:0007(anti tag coimmunoprecipitation)\|MI:0004(affinity chromatography technology)\|MI:0676(tandem affinity purification)\|MI:0096(pull down) |
| CUL3 | PRKDC | 0.86 | MI:0004(affinity chromatography technology)\|MI:0676(tandem affinity purification)\|MI:0030(cross-linking study) |
| CUL5 | PRKDC | 0.72 | MI:0004(affinity chromatography technology)\|MI:0676(tandem affinity purification) |
| CUL7 | PRPF6 | 0.73 | MI:0004(affinity chromatography technology) |
| CXCR4 | PTK2 | 0.74 | MI:0492(in vitro)\|MI:0493(in vivo)\|Affinity Capture-Western\|MI:0004(affinity chromatography technology) |
| DAPK3 | PRKCZ | 0.75 | MI:0492(in vitro)\|Reconstituted Complex\|MI:0096(pull down) |
| DCLRE1B | PRKDC | 0.72 | MI:0004(affinity chromatography technology)\|MI:0007(anti tag coimmunoprecipitation) |
| DCLRE1C | PRKDC | 0.9 | MI:0492(in vitro)\|MI:0493(in vivo)\|Reconstituted Complex\|Biochemical Activity\|Affinity Capture-Western\|MI:0415(enzymatic study)\|MI:0004(affinity chromatography technology)\|MI:0096(pull down)\|MI:0007(anti tag coimmunoprecipitation)\|MI:0006(anti bait coimmunoprecipitation) |
| DDA1 | PRKDC | 0.72 | MI:0007(anti tag coimmunoprecipitation)\|MI:0004(affinity chromatography technology) |
| DDX23 | PRPF6 | 0.89 | MI:0004(affinity chromatography technology)\|MI:0007(anti tag coimmunoprecipitation)\|bioid\|MI:0401(biochemical) |
| DEPDC1B | PRPF6 | 0.72 | MI:0007(anti tag coimmunoprecipitation)\|MI:0004(affinity chromatography technology) |
| DHX9 | PRKDC | 0.73 | MI:0415(enzymatic study)\|MI:0004(affinity chromatography technology)\|MI:0401(biochemical) |
| DLAT | PDK2 | 0.86 | MI:0492(in vitro)\|Reconstituted Complex\|MI:0096(pull down) |
| DNAJC5 | HGS | 0.87 | MI:0012(bioluminescence resonance energy transfer)\|MI:0055(fluorescent resonance energy transfer)\|bioid |
| DNAJC7 | PRKDC | 0.72 | MI:0004(affinity chromatography technology)\|MI:0007(anti tag coimmunoprecipitation) |
| DNM2 | PTK2 | 0.75 | MI:0493(in vivo)\|MI:0004(affinity chromatography technology) |
| DPP4 | PRKDC | 0.72 | MI:0004(affinity chromatography technology)\|bioid |
| EAPP | PRPF6 | 0.73 | MI:0004(affinity chromatography technology) |
| EBP | PTK2 | 0.72 | MI:0030(cross-linking study)\|bioid |
| ECT2 | PRKCZ | 0.72 | Affinity Capture-Western\|MI:0004(affinity chromatography technology) |
| EGFR | MET | 0.9 | MI:0492(in vitro)\|MI:0493(in vivo)\|MI:0006(anti bait coimmunoprecipitation)\|MI:0676(tandem affinity purification)\|MI:0004(affinity chromatography technology)\|MI:0424(protein kinase assay) |
| EGFR | HGS | 0.89 | MI:0492(in vitro)\|Affinity Capture-Western\|MI:0004(affinity chromatography technology)\|MI:0096(pull down)\|MI:0006(anti bait coimmunoprecipitation) |
| EGFR | PRKCZ | 0.87 | MI:0018(two hybrid)\|MI:0112(ubiquitin reconstruction)\|MI:0090(protein complementation assay) |
| EGFR | PRKDC | 0.73 | MI:0004(affinity chromatography technology) |
| EGLN3 | SIK3 | 0.72 | MI:0004(affinity chromatography technology)\|MI:0007(anti tag coimmunoprecipitation) |
| EHD1 | PRKDC | 0.72 | MI:0004(affinity chromatography technology)\|MI:0401(biochemical) |
| ELAVL1 | PTK2 | 0.7 | MI:0004(affinity chromatography technology) |
| ELK1 | PRKDC | 0.72 | MI:0007(anti tag coimmunoprecipitation)\|MI:0004(affinity chromatography technology) |
| ENO3 | KALRN | 0.72 | MI:0399(two hybrid fragment pooling approach)\|MI:0018(two hybrid) |
| EP300 | PRKDC | 0.85 | MI:0006(anti bait coimmunoprecipitation)\|MI:0004(affinity chromatography technology)\|MI:0096(pull down) |
| EPHA1 | PRKDC | 0.82 | MI:0004(affinity chromatography technology)\|MI:0007(anti tag coimmunoprecipitation) |
| EPS15 | HGS | 0.9 | MI:0492(in vitro)\|MI:0493(in vivo)\|Reconstituted Complex\|MI:0018(two hybrid)\|Co-fractionation\|MI:0096(pull down)\|MI:0004(affinity chromatography technology)\|MI:0401(biochemical) |
| ERBB2 | MET | 0.73 | MI:0090(protein complementation assay)\|MI:0004(affinity chromatography technology)\|MI:0428(imaging technique) |
| ESR1 | CDK8 | 0.75 | Reconstituted Complex\|MI:0096(pull down) |
| ESR1 | PRKDC | 0.74 | MI:0004(affinity chromatography technology) |
| ESR2 | CDK8 | 0.75 | Reconstituted Complex\|MI:0096(pull down) |
| F11R | PRKCZ | 0.8 | MI:0059(gst pull down)\|MI:0096(pull down)\|MI:0428(imaging technique) |
| FADD | PRKCZ | 0.83 | MI:0493(in vivo)\|Affinity Capture-Western\|MI:0004(affinity chromatography technology) |
| FAM118A | MOB3C | 0.73 | MI:0018(two hybrid) |
| FBXO6 | MET | 0.73 | MI:0004(affinity chromatography technology) |
| FECH | PDK2 | 0.82 | MI:0004(affinity chromatography technology)\|MI:0007(anti tag coimmunoprecipitation) |
| FEZ1 | PRKCZ | 0.83 | MI:0493(in vivo)\|MI:0018(two hybrid)\|Affinity Capture-Western\|MI:0004(affinity chromatography technology) |
| FEZ2 | PRKCZ | 0.73 | MI:0493(in vivo)\|Affinity Capture-Western\|MI:0004(affinity chromatography technology) |
| FLII | PRKDC | 0.72 | MI:0004(affinity chromatography technology)\|MI:0401(biochemical) |
| FOXD4 | PDK2 | 0.82 | MI:0004(affinity chromatography technology)\|MI:0007(anti tag coimmunoprecipitation) |
| FYN | PRPF6 | 0.72 | MI:0007(anti tag coimmunoprecipitation)\|MI:0004(affinity chromatography technology) |
| FZR1 | MET | 0.75 | MI:0055(fluorescent resonance energy transfer) |
| GAB2 | MET | 0.72 | Affinity Capture-Western\|MI:0004(affinity chromatography technology) |
| GABARAP | PRKCZ | 0.84 | MI:0007(anti tag coimmunoprecipitation)\|MI:0004(affinity chromatography technology)\|MI:0096(pull down) |
| GAK | TNK2 | 0.72 | MI:0004(affinity chromatography technology)\|MI:0007(anti tag coimmunoprecipitation) |
| GALT | TNK2 | 0.73 | MI:0018(two hybrid) |
| GCFC2 | PRPF6 | 0.83 | MI:0004(affinity chromatography technology)\|MI:0007(anti tag coimmunoprecipitation) |
| GIT1 | PTK2 | 0.9 | MI:0492(in vitro)\|MI:0493(in vivo)\|Affinity Capture-Western\|Reconstituted Complex\|MI:0004(affinity chromatography technology)\|MI:0096(pull down) |
| GJA1 | HGS | 0.75 | MI:0428(imaging technique)\|bioid |
| GLIS2 | MET | 0.75 | MI:0055(fluorescent resonance energy transfer) |
| GLMN | MET | 0.79 | MI:0493(in vivo)\|MI:0018(two hybrid)\|Reconstituted Complex\|Affinity Capture-Western\|MI:0004(affinity chromatography technology)\|MI:0096(pull down) |
| GMNN | CERK | 0.82 | MI:0004(affinity chromatography technology)\|MI:0007(anti tag coimmunoprecipitation) |
| GOLT1B | HGS | 0.72 | MI:0004(affinity chromatography technology)\|MI:0007(anti tag coimmunoprecipitation) |
| GPR156 | PRPF6 | 0.82 | MI:0004(affinity chromatography technology)\|MI:0007(anti tag coimmunoprecipitation) |
| GRB14 | MET | 0.75 | MI:0053(fluorescence polarization spectroscopy)\|MI:0096(pull down) |
| GRB14 | PRKCZ | 0.73 | MI:0493(in vivo)\|Affinity Capture-Western\|MI:0004(affinity chromatography technology) |
| GRB2 | HGS | 0.72 | MI:0007(anti tag coimmunoprecipitation)\|MI:0004(affinity chromatography technology) |
| GRB7 | PTK2 | 0.96 | MI:0492(in vitro)\|MI:0493(in vivo)\|Reconstituted Complex\|Affinity Capture-Western\|MI:0004(affinity chromatography technology)\|MI:0096(pull down)\|MI:0018(two hybrid) |
| GSK3B | PTK2 | 0.83 | MI:0493(in vivo)\|Affinity Capture-Western\|MI:0004(affinity chromatography technology) |
| GSN | PTK2 | 0.72 | Affinity Capture-Western\|MI:0004(affinity chromatography technology) |
| GTF2F1 | CDK8 | 0.82 | MI:0004(affinity chromatography technology)\|bioid |
| GTSE1 | TNK2 | 0.72 | MI:0004(affinity chromatography technology)\|MI:0007(anti tag coimmunoprecipitation) |
| H2AFX | PRKDC | 0.86 | MI:0006(anti bait coimmunoprecipitation)\|MI:0415(enzymatic study)\|MI:0004(affinity chromatography technology)\|MI:0096(pull down) |
| HAVCR2 | CERK | 0.82 | MI:0004(affinity chromatography technology)\|MI:0007(anti tag coimmunoprecipitation) |
| HCK | PTK2 | 0.72 | MI:0004(affinity chromatography technology)\|MI:0018(two hybrid) |
| HDAC11 | PRKDC | 0.72 | MI:0007(anti tag coimmunoprecipitation)\|MI:0004(affinity chromatography technology) |
| HDAC11 | PRPF6 | 0.72 | MI:0007(anti tag coimmunoprecipitation)\|MI:0004(affinity chromatography technology) |
| HDGF | PRKDC | 0.75 | MI:0004(affinity chromatography technology)\|MI:0096(pull down)\|MI:0676(tandem affinity purification) |
| HDLBP | PRKDC | 0.72 | Affinity Capture-Western\|MI:0004(affinity chromatography technology) |
| HEPACAM2 | CERK | 0.83 | MI:0004(affinity chromatography technology)\|MI:0007(anti tag coimmunoprecipitation) |
| HGH1 | HGS | 0.72 | MI:0004(affinity chromatography technology)\|MI:0401(biochemical) |
| HGS | TSG101 | 0.97 | MI:0493(in vivo)\|Affinity Capture-Western\|MI:0007(anti tag coimmunoprecipitation)\|MI:0416(fluorescence microscopy)\|MI:0018(two hybrid)\|MI:0004(affinity chromatography technology)\|MI:0096(pull down)\|MI:0428(imaging technique)\|MI:0114(x-ray crystallography)\|MI:0065(isothermal titration calorimetry) |
| HGS | STAM | 0.97 | MI:0493(in vivo)\|Affinity Capture-Western\|Co-fractionation\|MI:0018(two hybrid)\|MI:0004(affinity chromatography technology)\|MI:0096(pull down)\|MI:0428(imaging technique)\|MI:0071(molecular sieving)\|MI:0114(x-ray crystallography)\|MI:0038(dynamic light scattering)\|MI:0028(cosedimentation in solution)\|MI:0067(light scattering)\|MI:0007(anti tag coimmunoprecipitation)\|MI:0401(biochemical) |
| HGS | STAM2 | 0.96 | Co-fractionation\|Affinity Capture-Western\|MI:0018(two hybrid)\|MI:0004(affinity chromatography technology)\|MI:0428(imaging technique)\|MI:0401(biochemical) |
| HGS | HAP1 | 0.9 | MI:0492(in vitro)\|MI:0493(in vivo)\|MI:0018(two hybrid)\|Reconstituted Complex\|Affinity Capture-Western\|MI:0004(affinity chromatography technology)\|MI:0096(pull down) |
| HGS | SNX1 | 0.89 | MI:0493(in vivo)\|Reconstituted Complex\|MI:0096(pull down)\|MI:0004(affinity chromatography technology) |
| HGS | NEDD4 | 0.88 | MI:0492(in vitro)\|MI:0493(in vivo)\|Biochemical Activity\|MI:0007(anti tag coimmunoprecipitation)\|MI:0415(enzymatic study)\|MI:0004(affinity chromatography technology) |
| HGS | ACTN4 | 0.88 | MI:0004(affinity chromatography technology)\|MI:0018(two hybrid)\|MI:0428(imaging technique)\|MI:0401(biochemical) |
| HGS | HSPA8 | 0.87 | Reconstituted Complex\|MI:0096(pull down)\|MI:0004(affinity chromatography technology) |
| HGS | GGA2 | 0.87 | Reconstituted Complex\|MI:0018(two hybrid)\|MI:0096(pull down) |
| HGS | MAP3K7 | 0.86 | MI:0492(in vitro)\|MI:0493(in vivo)\|Reconstituted Complex\|MI:0096(pull down) |
| HGS | DLG4 | 0.86 | MI:0493(in vivo)\|MI:0018(two hybrid)\|Affinity Capture-Western\|MI:0004(affinity chromatography technology) |
| HGS | PAK1 | 0.85 | MI:0493(in vivo)\|Reconstituted Complex\|MI:0096(pull down) |
| HGS | APP | 0.85 | Reconstituted Complex\|MI:0096(pull down) |
| HGS | UBA1 | 0.84 | Reconstituted Complex\|MI:0096(pull down) |
| HGS | UBQLN1 | 0.83 | MI:0018(two hybrid)\|MI:0004(affinity chromatography technology) |
| HGS | CCDC114 | 0.83 | MI:0018(two hybrid)\|MI:0004(affinity chromatography technology) |
| HGS | ARRB2 | 0.82 | MI:0007(anti tag coimmunoprecipitation)\|MI:0004(affinity chromatography technology) |
| HGS | MET | 0.76 | MI:0493(in vivo)\|MI:0004(affinity chromatography technology) |
| HGS | APLP2 | 0.75 | Reconstituted Complex\|MI:0096(pull down) |
| HGS | ATP2A2 | 0.75 | Reconstituted Complex\|MI:0096(pull down) |
| HGS | ARL6IP1 | 0.75 | Reconstituted Complex\|MI:0096(pull down) |
| HGS | BSG | 0.75 | Reconstituted Complex\|MI:0096(pull down) |
| HGS | BRINP3 | 0.75 | Reconstituted Complex\|MI:0096(pull down) |
| HGS | AHCYL1 | 0.75 | Reconstituted Complex\|MI:0096(pull down) |
| HGS | CASK | 0.75 | Reconstituted Complex\|MI:0096(pull down) |
| HGS | TJP2 | 0.75 | Reconstituted Complex\|MI:0096(pull down) |
| HGS | MAP3K10 | 0.75 | Reconstituted Complex\|MI:0096(pull down) |
| HGS | STXBP1 | 0.75 | Reconstituted Complex\|MI:0096(pull down) |
| HGS | SCRN1 | 0.75 | Reconstituted Complex\|MI:0096(pull down) |
| HGS | CRMP1 | 0.75 | Reconstituted Complex\|MI:0096(pull down) |
| HGS | DCTN2 | 0.75 | Reconstituted Complex\|MI:0096(pull down) |
| HGS | GFAP | 0.75 | Reconstituted Complex\|MI:0096(pull down) |
| HGS | MARK4 | 0.75 | Reconstituted Complex\|MI:0096(pull down) |
| HGS | PPP1R16A | 0.75 | Reconstituted Complex\|MI:0096(pull down) |
| HGS | RHOBTB3 | 0.75 | Reconstituted Complex\|MI:0096(pull down) |
| HGS | TUBB2A | 0.75 | Reconstituted Complex\|MI:0096(pull down) |
| HGS | ESRRG | 0.75 | Reconstituted Complex\|MI:0096(pull down) |
| HGS | ILKAP | 0.75 | Reconstituted Complex\|MI:0096(pull down) |
| HGS | PPP1R7 | 0.75 | Reconstituted Complex\|MI:0096(pull down) |
| HGS | RSU1 | 0.75 | Reconstituted Complex\|MI:0096(pull down) |
| HGS | ACLY | 0.75 | Reconstituted Complex\|MI:0096(pull down) |
| HGS | CBS | 0.75 | Reconstituted Complex\|MI:0096(pull down) |
| HGS | DECR1 | 0.75 | Reconstituted Complex\|MI:0096(pull down) |
| HGS | MAT2A | 0.75 | Reconstituted Complex\|MI:0096(pull down) |
| HGS | MTHFD1L | 0.75 | Reconstituted Complex\|MI:0096(pull down) |
| HGS | OSBPL5 | 0.75 | Reconstituted Complex\|MI:0096(pull down) |
| HGS | PLD3 | 0.75 | Reconstituted Complex\|MI:0096(pull down) |
| HGS | PFKM | 0.75 | Reconstituted Complex\|MI:0096(pull down) |
| HGS | HNRNPDL | 0.75 | Reconstituted Complex\|MI:0096(pull down) |
| HGS | RPS3A | 0.75 | Reconstituted Complex\|MI:0096(pull down) |
| HGS | SF3B3 | 0.75 | Reconstituted Complex\|MI:0096(pull down) |
| HGS | LINC00265 | 0.75 | Reconstituted Complex\|MI:0096(pull down) |
| HGS | PTCD3 | 0.75 | Reconstituted Complex\|MI:0096(pull down) |
| HGS | ZNF302 | 0.75 | Reconstituted Complex\|MI:0096(pull down) |
| HGS | MEST | 0.75 | Reconstituted Complex\|MI:0096(pull down) |
| HGS | TMCC2 | 0.75 | Reconstituted Complex\|MI:0096(pull down) |
| HGS | ATP1A1 | 0.75 | Reconstituted Complex\|MI:0096(pull down) |
| HGS | TRAP1 | 0.75 | Reconstituted Complex\|MI:0096(pull down) |
| HGS | TUBB | 0.75 | Reconstituted Complex\|MI:0096(pull down) |
| HGS | MED7 | 0.74 | MI:0018(two hybrid) |
| HGS | DAZAP2 | 0.74 | MI:0018(two hybrid) |
| HGS | UBE2I | 0.74 | MI:0018(two hybrid) |
| HGS | CCDC33 | 0.74 | MI:0018(two hybrid) |
| HGS | MIF4GD | 0.74 | MI:0018(two hybrid) |
| HGS | EXOC7 | 0.74 | MI:0018(two hybrid) |
| HGS | USHBP1 | 0.74 | MI:0018(two hybrid) |
| HGS | CEP55 | 0.74 | MI:0018(two hybrid) |
| HGS | BEGAIN | 0.73 | MI:0018(two hybrid) |
| HGS | EHMT2 | 0.73 | MI:0018(two hybrid) |
| HGS | EXOC8 | 0.73 | MI:0018(two hybrid) |
| HGS | GKAP1 | 0.73 | MI:0018(two hybrid) |
| HGS | ITCH | 0.73 | MI:0004(affinity chromatography technology) |
| HGS | LURAP1 | 0.73 | MI:0018(two hybrid) |
| HGS | TMEM189 | 0.73 | MI:0004(affinity chromatography technology)\|MI:0055(fluorescent resonance energy transfer) |
| HGS | ABI2 | 0.73 | MI:0018(two hybrid) |
| HGS | NDC80 | 0.73 | MI:0018(two hybrid) |
| HGS | ING5 | 0.73 | MI:0018(two hybrid) |
| HGS | POGZ | 0.73 | MI:0018(two hybrid) |
| HGS | MRFAP1L1 | 0.73 | MI:0018(two hybrid) |
| HGS | INTS4 | 0.73 | MI:0018(two hybrid) |
| HGS | KRT40 | 0.73 | MI:0018(two hybrid) |
| HGS | NUP54 | 0.73 | MI:0018(two hybrid) |
| HGS | P4HA3 | 0.73 | MI:0018(two hybrid) |
| HGS | LITAF | 0.73 | MI:0018(two hybrid) |
| HGS | MAPK1IP1L | 0.73 | MI:0018(two hybrid) |
| HGS | CEP63 | 0.73 | MI:0018(two hybrid) |
| HGS | CDR2 | 0.73 | MI:0018(two hybrid) |
| HGS | CRX | 0.73 | MI:0018(two hybrid) |
| HGS | TEKT1 | 0.73 | MI:0018(two hybrid) |
| HGS | KRT33B | 0.73 | MI:0018(two hybrid) |
| HGS | KRT13 | 0.73 | MI:0018(two hybrid) |
| HGS | C1orf94 | 0.73 | MI:0018(two hybrid) |
| HGS | DYDC1 | 0.73 | MI:0018(two hybrid) |
| HGS | TRIM27 | 0.73 | MI:0018(two hybrid) |
| HGS | MAGED1 | 0.73 | MI:0018(two hybrid) |
| HGS | TRAF4 | 0.73 | MI:0018(two hybrid) |
| HGS | KIAA0753 | 0.73 | MI:0018(two hybrid) |
| HGS | FAM168A | 0.73 | MI:0018(two hybrid) |
| HGS | NUP62 | 0.73 | MI:0018(two hybrid) |
| HGS | TFG | 0.73 | MI:0018(two hybrid) |
| HGS | VPS37B | 0.73 | MI:0018(two hybrid) |
| HGS | TRIM17 | 0.73 | MI:0018(two hybrid) |
| HGS | TRIM69 | 0.73 | MI:0018(two hybrid) |
| HGS | SCAMP3 | 0.72 | Affinity Capture-Western\|MI:0004(affinity chromatography technology) |
| HGS | WDR77 | 0.72 | MI:0004(affinity chromatography technology)\|MI:0401(biochemical) |
| HGS | REPIN1 | 0.72 | MI:0004(affinity chromatography technology)\|MI:0401(biochemical) |
| HIF1AN | SIK3 | 0.72 | MI:0004(affinity chromatography technology)\|MI:0007(anti tag coimmunoprecipitation) |
| HIST1H3A | PRKDC | 0.89 | MI:0004(affinity chromatography technology)\|MI:0428(imaging technique)\|MI:0676(tandem affinity purification)\|bioid\|MI:0401(biochemical) |
| HIST4H4 | PRPF6 | 0.73 | MI:0004(affinity chromatography technology) |
| HIST4H4 | PRKDC | 0.73 | MI:0004(affinity chromatography technology) |
| HOXB7 | PRKDC | 0.75 | MI:0096(pull down)\|MI:0006(anti bait coimmunoprecipitation)\|MI:0004(affinity chromatography technology) |
| HSCB | STK24 | 0.72 | MI:0006(anti bait coimmunoprecipitation)\|MI:0004(affinity chromatography technology) |
| HSH2D | TNK2 | 0.88 | MI:0493(in vivo)\|MI:0018(two hybrid)\|Biochemical Activity\|Affinity Capture-Western\|MI:0415(enzymatic study)\|MI:0004(affinity chromatography technology) |
| HSP90AB1 | PRKCZ | 0.82 | MI:0007(anti tag coimmunoprecipitation)\|MI:0004(affinity chromatography technology) |
| HSP90AB1 | PRKDC | 0.72 | MI:0006(anti bait coimmunoprecipitation)\|MI:0004(affinity chromatography technology) |
| HSP90AB2P | TNK2 | 0.85 | MI:0492(in vitro)\|MI:0493(in vivo)\|MI:0004(affinity chromatography technology)\|MI:0007(anti tag coimmunoprecipitation) |
| HSPA5 | PRKDC | 0.72 | MI:0004(affinity chromatography technology)\|MI:0007(anti tag coimmunoprecipitation) |
| HSPA8 | PRKDC | 0.82 | MI:0004(affinity chromatography technology)\|MI:0401(biochemical) |
| HSPBP1 | HGS | 0.82 | MI:0004(affinity chromatography technology)\|MI:0007(anti tag coimmunoprecipitation) |
| HTT | PDK2 | 0.73 | MI:0018(two hybrid) |
| HTT | HGS | 0.72 | MI:0007(anti tag coimmunoprecipitation)\|MI:0004(affinity chromatography technology) |
| IGF1R | PTK2 | 0.72 | Affinity Capture-Western\|MI:0004(affinity chromatography technology) |
| IKBKG | PRKDC | 0.86 | MI:0676(tandem affinity purification)\|MI:0007(anti tag coimmunoprecipitation)\|MI:0004(affinity chromatography technology)\|MI:0415(enzymatic study) |
| IL20RA | CERK | 0.82 | MI:0004(affinity chromatography technology)\|MI:0007(anti tag coimmunoprecipitation) |
| IL2RB | HGS | 0.88 | Affinity Capture-Western\|Reconstituted Complex\|MI:0004(affinity chromatography technology)\|MI:0096(pull down) |
| ILF2 | PRKDC | 0.89 | MI:0492(in vitro)\|MI:0493(in vivo)\|MI:0025(copurification)\|Affinity Capture-Western\|MI:0004(affinity chromatography technology)\|MI:0401(biochemical) |
| ILK | PRKDC | 0.88 | MI:0676(tandem affinity purification)\|MI:0004(affinity chromatography technology)\|MI:0007(anti tag coimmunoprecipitation)\|MI:0096(pull down) |
| INPPL1 | MET | 0.89 | MI:0492(in vitro)\|MI:0493(in vivo)\|MI:0018(two hybrid)\|MI:0004(affinity chromatography technology)\|MI:0053(fluorescence polarization spectroscopy)\|MI:0096(pull down) |
| IQCB1 | PRKDC | 0.72 | MI:0007(anti tag coimmunoprecipitation)\|MI:0004(affinity chromatography technology) |
| IRAK1 | PRKCZ | 0.73 | MI:0493(in vivo)\|Affinity Capture-Western\|MI:0004(affinity chromatography technology) |
| ISG15 | PRKDC | 0.78 | MI:0096(pull down)\|MI:0004(affinity chromatography technology) |
| ITGB4 | PTK2 | 0.83 | MI:0492(in vitro)\|MI:0007(anti tag coimmunoprecipitation)\|MI:0006(anti bait coimmunoprecipitation) |
| ITGB5 | PTK2 | 0.95 | Reconstituted Complex\|Affinity Capture-Western\|MI:0004(affinity chromatography technology)\|MI:0096(pull down) |
| JAK1 | PRKCZ | 0.77 | MI:0492(in vitro)\|MI:0493(in vivo)\|MI:0004(affinity chromatography technology) |
| KALRN | DISC1 | 0.83 | MI:0018(two hybrid)\|MI:0399(two hybrid fragment pooling approach) |
| KALRN | NDEL1 | 0.83 | MI:0018(two hybrid)\|MI:0399(two hybrid fragment pooling approach) |
| KALRN | PAM | 0.79 | MI:0018(two hybrid) |
| KCTD17 | CERK | 0.83 | MI:0004(affinity chromatography technology)\|MI:0007(anti tag coimmunoprecipitation) |
| KDELR2 | MET | 0.75 | MI:0055(fluorescent resonance energy transfer) |
| KIAA1143 | PRPF6 | 0.82 | MI:0004(affinity chromatography technology)\|MI:0007(anti tag coimmunoprecipitation) |
| KIF21B | PRPF6 | 0.72 | MI:0004(affinity chromatography technology)\|MI:0007(anti tag coimmunoprecipitation) |
| KRT15 | HGS | 0.73 | MI:0018(two hybrid) |
| KRT18 | HGS | 0.74 | MI:0018(two hybrid) |
| KRT19 | HGS | 0.83 | MI:0018(two hybrid)\|bioid |
| KRT31 | HGS | 0.83 | MI:0018(two hybrid)\|MI:0004(affinity chromatography technology) |
| KRT38 | HGS | 0.74 | MI:0018(two hybrid) |
| KRT6A | HGS | 0.73 | MI:0018(two hybrid) |
| LCK | PTK2 | 0.77 | MI:0492(in vitro)\|MI:0493(in vivo)\|MI:0018(two hybrid) |
| LCK | MET | 0.75 | MI:0053(fluorescence polarization spectroscopy)\|MI:0096(pull down) |
| LDOC1 | HGS | 0.74 | MI:0018(two hybrid) |
| LGALS9 | MET | 0.83 | MI:0004(affinity chromatography technology)\|MI:0007(anti tag coimmunoprecipitation) |
| LLGL1 | PRKCZ | 0.92 | MI:0007(anti tag coimmunoprecipitation)\|MI:0004(affinity chromatography technology)\|MI:0096(pull down) |
| LMNA | PRPF6 | 0.82 | MI:0004(affinity chromatography technology)\|MI:0018(two hybrid) |
| LMNA | PRKDC | 0.72 | MI:0004(affinity chromatography technology)\|MI:0007(anti tag coimmunoprecipitation) |
| LPAR4 | PRKDC | 0.72 | MI:0004(affinity chromatography technology)\|MI:0007(anti tag coimmunoprecipitation) |
| LPXN | PTK2 | 0.88 | MI:0493(in vivo)\|MI:0018(two hybrid)\|MI:0004(affinity chromatography technology)\|MI:0007(anti tag coimmunoprecipitation) |
| LRIG1 | MET | 0.86 | Affinity Capture-Western\|MI:0004(affinity chromatography technology)\|MI:0428(imaging technique)\|MI:0006(anti bait coimmunoprecipitation) |
| LRRFIP1 | PRKDC | 0.72 | MI:0004(affinity chromatography technology)\|MI:0401(biochemical) |
| LRRK2 | PRKDC | 0.83 | MI:0004(affinity chromatography technology)\|MI:0007(anti tag coimmunoprecipitation) |
| LRRK2 | PRPF6 | 0.82 | MI:0676(tandem affinity purification)\|MI:0007(anti tag coimmunoprecipitation) |
| LSM1 | PRPF6 | 0.7 | bioid |
| LSM2 | PRPF6 | 0.7 | bioid |
| LSM3 | PRPF6 | 0.7 | MI:0004(affinity chromatography technology) |
| LSM4 | PRPF6 | 0.82 | MI:0004(affinity chromatography technology)\|bioid |
| LSM5 | PRPF6 | 0.7 | MI:0004(affinity chromatography technology) |
| LSM6 | PRPF6 | 0.7 | MI:0004(affinity chromatography technology) |
| LSM7 | PRPF6 | 0.7 | MI:0004(affinity chromatography technology) |
| LTBR | HGS | 0.83 | MI:0004(affinity chromatography technology)\|MI:0007(anti tag coimmunoprecipitation) |
| LYN | MET | 0.87 | MI:0053(fluorescence polarization spectroscopy)\|MI:0096(pull down)\|bioid |
| LYST | HGS | 0.87 | MI:0492(in vitro)\|MI:0018(two hybrid)\|Reconstituted Complex\|MI:0096(pull down) |
| LZIC | CDK8 | 0.72 | MI:0004(affinity chromatography technology)\|MI:0007(anti tag coimmunoprecipitation) |
| MAGIX | STK24 | 0.83 | MI:0004(affinity chromatography technology)\|MI:0007(anti tag coimmunoprecipitation) |
| MAP2K3 | MET | 0.75 | MI:0055(fluorescent resonance energy transfer) |
| MAP2K5 | PRKCZ | 0.88 | MI:0493(in vivo)\|Affinity Capture-Western\|Reconstituted Complex\|MI:0004(affinity chromatography technology)\|MI:0096(pull down) |
| MAP2K5 | MET | 0.75 | MI:0055(fluorescent resonance energy transfer) |
| MAP3K3 | PRKDC | 0.72 | MI:0676(tandem affinity purification)\|MI:0007(anti tag coimmunoprecipitation) |
| MAPK7 | PRKCZ | 0.73 | MI:0415(enzymatic study)\|MI:0018(two hybrid)\|MI:0004(affinity chromatography technology) |
| MAPK8IP3 | PTK2 | 0.86 | MI:0492(in vitro)\|MI:0493(in vivo)\|Affinity Capture-Western\|MI:0004(affinity chromatography technology)\|MI:0096(pull down) |
| MAU2 | MOB3C | 0.73 | MI:0004(affinity chromatography technology) |
| MCC | STK24 | 0.76 | Affinity Capture-MS\|MI:0004(affinity chromatography technology)\|MI:0006(anti bait coimmunoprecipitation) |
| MDC1 | PRKDC | 0.7 | MI:0004(affinity chromatography technology)\|MI:0096(pull down)\|MI:0428(imaging technique) |
| MDM2 | PRPF6 | 0.73 | MI:0004(affinity chromatography technology) |
| MED10 | CDK8 | 0.97 | Affinity Capture-MS\|Affinity Capture-Western\|MI:0007(anti tag coimmunoprecipitation)\|MI:0004(affinity chromatography technology)\|MI:0676(tandem affinity purification)\|MI:0096(pull down)\|bioid\|MI:0401(biochemical) |
| MED19 | CDK8 | 0.89 | Affinity Capture-MS\|MI:0007(anti tag coimmunoprecipitation)\|MI:0004(affinity chromatography technology)\|MI:0096(pull down)\|bioid |
| MED21 | CDK8 | 0.9 | MI:0493(in vivo)\|Co-fractionation\|Affinity Capture-Western\|Affinity Capture-MS\|MI:0004(affinity chromatography technology)\|MI:0676(tandem affinity purification)\|MI:0007(anti tag coimmunoprecipitation)\|MI:0096(pull down)\|bioid\|MI:0401(biochemical) |
| MED25 | CDK8 | 0.9 | MI:0493(in vivo)\|Affinity Capture-MS\|MI:0004(affinity chromatography technology)\|MI:0007(anti tag coimmunoprecipitation)\|MI:0096(pull down)\|MI:0006(anti bait coimmunoprecipitation)\|bioid |
| MED26 | CDK8 | 0.9 | MI:0492(in vitro)\|Affinity Capture-MS\|Affinity Capture-Western\|MI:0007(anti tag coimmunoprecipitation)\|MI:0004(affinity chromatography technology)\|MI:0096(pull down)\|MI:0006(anti bait coimmunoprecipitation) |
| MED28 | CDK8 | 0.9 | Affinity Capture-MS\|MI:0004(affinity chromatography technology)\|MI:0676(tandem affinity purification)\|MI:0007(anti tag coimmunoprecipitation)\|MI:0096(pull down)\|bioid |
| MED29 | CDK8 | 0.9 | Affinity Capture-MS\|MI:0007(anti tag coimmunoprecipitation)\|MI:0004(affinity chromatography technology)\|MI:0676(tandem affinity purification)\|MI:0096(pull down)\|bioid |
| MED30 | CDK8 | 0.89 | MI:0004(affinity chromatography technology)\|MI:0676(tandem affinity purification)\|MI:0007(anti tag coimmunoprecipitation)\|MI:0096(pull down)\|bioid |
| MED6 | CDK8 | 0.97 | Affinity Capture-MS\|Affinity Capture-Western\|MI:0004(affinity chromatography technology)\|MI:0676(tandem affinity purification)\|MI:0007(anti tag coimmunoprecipitation)\|MI:0096(pull down)\|bioid\|MI:0401(biochemical) |
| MED8 | CDK8 | 0.9 | MI:0492(in vitro)\|MI:0676(tandem affinity purification)\|MI:0004(affinity chromatography technology)\|MI:0007(anti tag coimmunoprecipitation)\|MI:0096(pull down)\|bioid\|MI:0401(biochemical) |
| MED9 | CDK8 | 0.9 | MI:0492(in vitro)\|Affinity Capture-MS\|MI:0007(anti tag coimmunoprecipitation)\|MI:0004(affinity chromatography technology)\|MI:0676(tandem affinity purification)\|MI:0096(pull down)\|bioid |
| MEPCE | PRPF6 | 0.72 | Affinity Capture-MS\|MI:0004(affinity chromatography technology) |
| MET | GAB1 | 0.95 | MI:0492(in vitro)\|MI:0493(in vivo)\|Affinity Capture-Western\|MI:0007(anti tag coimmunoprecipitation)\|MI:0004(affinity chromatography technology) |
| MET | GRB2 | 0.9 | MI:0492(in vitro)\|MI:0493(in vivo)\|MI:0018(two hybrid)\|Reconstituted Complex\|Affinity Capture-Western\|MI:0004(affinity chromatography technology)\|MI:0096(pull down)\|MI:0114(x-ray crystallography) |
| MET | HGF | 0.9 | MI:0492(in vitro)\|MI:0493(in vivo)\|Reconstituted Complex\|Biochemical Activity\|Affinity Capture-Western\|MI:0114(x-ray crystallography)\|MI:0411(enzyme linked immunosorbent assay)\|MI:0055(fluorescent resonance energy transfer)\|MI:0415(enzymatic study)\|MI:0004(affinity chromatography technology)\|MI:0096(pull down)\|MI:0030(cross-linking study)\|MI:0440(saturation binding)\|MI:0826(x ray scattering) |
| MET | PTPN1 | 0.88 | MI:0006(anti bait coimmunoprecipitation)\|MI:0434(phosphatase assay)\|MI:0030(cross-linking study) |
| MET | CRK | 0.87 | MI:0053(fluorescence polarization spectroscopy)\|MI:0004(affinity chromatography technology)\|MI:0096(pull down) |
| MET | PLCG1 | 0.86 | MI:0492(in vitro)\|MI:0053(fluorescence polarization spectroscopy)\|MI:0096(pull down) |
| MET | DNAJA3 | 0.85 | MI:0018(two hybrid)\|MI:0006(anti bait coimmunoprecipitation)\|MI:0096(pull down)\|MI:0663(confocal microscopy) |
| MET | PIK3R1 | 0.85 | MI:0492(in vitro)\|MI:0053(fluorescence polarization spectroscopy)\|MI:0096(pull down) |
| MET | ERBB3 | 0.82 | MI:0004(affinity chromatography technology)\|MI:0006(anti bait coimmunoprecipitation) |
| MET | SH2B1 | 0.75 | MI:0053(fluorescence polarization spectroscopy)\|MI:0096(pull down) |
| MET | SHD | 0.75 | MI:0053(fluorescence polarization spectroscopy)\|MI:0096(pull down) |
| MET | FGR | 0.75 | MI:0053(fluorescence polarization spectroscopy)\|MI:0096(pull down) |
| MET | SH3BP2 | 0.75 | MI:0053(fluorescence polarization spectroscopy)\|MI:0096(pull down) |
| MET | PLCG2 | 0.75 | MI:0053(fluorescence polarization spectroscopy)\|MI:0096(pull down) |
| MET | TNS2 | 0.75 | MI:0053(fluorescence polarization spectroscopy)\|MI:0096(pull down) |
| MET | TNS1 | 0.75 | MI:0053(fluorescence polarization spectroscopy)\|MI:0096(pull down) |
| MET | BTK | 0.75 | MI:0053(fluorescence polarization spectroscopy)\|MI:0096(pull down) |
| MET | SH2D3C | 0.75 | MI:0053(fluorescence polarization spectroscopy)\|MI:0096(pull down) |
| MET | HCK | 0.75 | MI:0053(fluorescence polarization spectroscopy)\|MI:0096(pull down) |
| MET | VAV2 | 0.75 | MI:0053(fluorescence polarization spectroscopy)\|MI:0096(pull down) |
| MET | TNS3 | 0.75 | MI:0053(fluorescence polarization spectroscopy)\|MI:0096(pull down) |
| MET | HSH2D | 0.75 | MI:0053(fluorescence polarization spectroscopy)\|MI:0096(pull down) |
| MET | PIK3R3 | 0.75 | MI:0053(fluorescence polarization spectroscopy)\|MI:0096(pull down) |
| MET | GRB7 | 0.75 | MI:0053(fluorescence polarization spectroscopy)\|MI:0096(pull down) |
| MET | SHC3 | 0.75 | MI:0053(fluorescence polarization spectroscopy)\|MI:0096(pull down) |
| MET | STAP1 | 0.75 | MI:0053(fluorescence polarization spectroscopy)\|MI:0096(pull down) |
| MET | YES1 | 0.75 | MI:0053(fluorescence polarization spectroscopy)\|MI:0096(pull down) |
| MET | FES | 0.75 | MI:0053(fluorescence polarization spectroscopy)\|MI:0096(pull down) |
| MET | SOCS5 | 0.75 | MI:0053(fluorescence polarization spectroscopy)\|MI:0096(pull down) |
| MET | SYK | 0.75 | MI:0053(fluorescence polarization spectroscopy)\|MI:0096(pull down) |
| MET | ZAP70 | 0.75 | MI:0053(fluorescence polarization spectroscopy)\|MI:0096(pull down) |
| MET | SOCS2 | 0.75 | MI:0053(fluorescence polarization spectroscopy)\|MI:0096(pull down) |
| MET | ABL2 | 0.75 | MI:0053(fluorescence polarization spectroscopy)\|MI:0096(pull down) |
| MET | MATK | 0.75 | MI:0053(fluorescence polarization spectroscopy)\|MI:0096(pull down) |
| MET | ITK | 0.75 | MI:0053(fluorescence polarization spectroscopy)\|MI:0096(pull down) |
| MET | RASA1 | 0.75 | MI:0053(fluorescence polarization spectroscopy)\|MI:0096(pull down) |
| MET | TERT | 0.75 | MI:0055(fluorescent resonance energy transfer) |
| MET | STK11 | 0.75 | MI:0055(fluorescent resonance energy transfer) |
| MET | CCND2 | 0.75 | MI:0055(fluorescent resonance energy transfer) |
| MET | EPHA2 | 0.75 | MI:0055(fluorescent resonance energy transfer) |
| MET | CDKN2B | 0.75 | MI:0055(fluorescent resonance energy transfer) |
| MET | CDK6 | 0.75 | MI:0055(fluorescent resonance energy transfer) |
| MET | CDK4 | 0.75 | MI:0055(fluorescent resonance energy transfer) |
| MET | FGFR4 | 0.75 | MI:0055(fluorescent resonance energy transfer) |
| MET | LATS2 | 0.75 | MI:0055(fluorescent resonance energy transfer) |
| MET | STAT3 | 0.73 | MI:0493(in vivo)\|Affinity Capture-Western\|MI:0004(affinity chromatography technology) |
| MET | HSP90AA1 | 0.73 | MI:0004(affinity chromatography technology) |
| MET | SOS1 | 0.72 | Affinity Capture-Western\|MI:0004(affinity chromatography technology) |
| MET | PLXNB3 | 0.72 | MI:0007(anti tag coimmunoprecipitation)\|MI:0006(anti bait coimmunoprecipitation) |
| METTL1 | PRKDC | 0.72 | MI:0004(affinity chromatography technology)\|MI:0401(biochemical) |
| METTL14 | PRKDC | 0.72 | MI:0007(anti tag coimmunoprecipitation)\|MI:0004(affinity chromatography technology) |
| METTL3 | PRKDC | 0.72 | MI:0007(anti tag coimmunoprecipitation)\|MI:0004(affinity chromatography technology) |
| MLH1 | PRKDC | 0.72 | Affinity Capture-MS\|MI:0004(affinity chromatography technology) |
| MOB3C | ZBTB10 | 0.73 | MI:0018(two hybrid) |
| MRAP2 | CERK | 0.82 | MI:0004(affinity chromatography technology)\|MI:0007(anti tag coimmunoprecipitation) |
| MYC | PRPF6 | 0.87 | MI:0676(tandem affinity purification)\|MI:0006(anti bait coimmunoprecipitation)\|MI:0004(affinity chromatography technology) |
| MYC | PRKDC | 0.82 | MI:0676(tandem affinity purification)\|MI:0004(affinity chromatography technology) |
| MYC | MET | 0.75 | MI:0055(fluorescent resonance energy transfer) |
| MYC | SPEG | 0.72 | MI:0676(tandem affinity purification)\|MI:0004(affinity chromatography technology) |
| NBN | PRKDC | 0.71 | MI:0492(in vitro)\|MI:0493(in vivo)\|MI:0096(pull down)\|MI:0004(affinity chromatography technology) |
| NCK1 | PTK2 | 0.88 | Reconstituted Complex\|Affinity Capture-Western\|MI:0004(affinity chromatography technology)\|MI:0096(pull down) |
| NCK1 | MET | 0.75 | MI:0053(fluorescence polarization spectroscopy)\|MI:0096(pull down) |
| NCK2 | PTK2 | 0.89 | MI:0493(in vivo)\|Reconstituted Complex\|Affinity Capture-Western\|MI:0004(affinity chromatography technology)\|MI:0096(pull down) |
| NCK2 | TNK2 | 0.89 | MI:0004(affinity chromatography technology)\|MI:0007(anti tag coimmunoprecipitation) |
| NCK2 | MET | 0.75 | MI:0053(fluorescence polarization spectroscopy)\|MI:0096(pull down) |
| NCOA6 | PRKDC | 0.87 | MI:0492(in vitro)\|Affinity Capture-MS\|Biochemical Activity\|MI:0415(enzymatic study)\|MI:0004(affinity chromatography technology) |
| NEDD4L | HGS | 0.75 | MI:0415(enzymatic study)\|MI:0004(affinity chromatography technology) |
| NEDD9 | PTK2 | 0.88 | MI:0492(in vitro)\|MI:0493(in vivo)\|MI:0018(two hybrid)\|Affinity Capture-Western\|MI:0004(affinity chromatography technology) |
| NEK4 | PRKDC | 0.72 | MI:0007(anti tag coimmunoprecipitation)\|MI:0004(affinity chromatography technology) |
| NEK4 | STK24 | 0.72 | MI:0007(anti tag coimmunoprecipitation)\|MI:0004(affinity chromatography technology) |
| NF2 | HGS | 0.9 | MI:0492(in vitro)\|MI:0493(in vivo)\|MI:0018(two hybrid)\|Reconstituted Complex\|Affinity Capture-Western\|MI:0004(affinity chromatography technology)\|MI:0096(pull down)\|MI:0012(bioluminescence resonance energy transfer)\|MI:0663(confocal microscopy)\|MI:0006(anti bait coimmunoprecipitation)\|MI:0090(protein complementation assay)\|MI:0055(fluorescent resonance energy transfer) |
| NF2 | MET | 0.75 | MI:0055(fluorescent resonance energy transfer) |
| NFATC2 | PRKCZ | 0.77 | MI:0493(in vivo)\|Affinity Capture-Western\|Biochemical Activity\|MI:0415(enzymatic study)\|MI:0004(affinity chromatography technology) |
| NFKB1 | PRKDC | 0.82 | MI:0007(anti tag coimmunoprecipitation)\|MI:0676(tandem affinity purification) |
| NFKB2 | PRKDC | 0.72 | MI:0676(tandem affinity purification)\|MI:0007(anti tag coimmunoprecipitation) |
| NHEJ1 | PRKDC | 0.75 | MI:0007(anti tag coimmunoprecipitation)\|MI:0415(enzymatic study) |
| NIPSNAP2 | PRKDC | 0.72 | MI:0007(anti tag coimmunoprecipitation)\|MI:0004(affinity chromatography technology) |
| NMI | HGS | 0.74 | MI:0018(two hybrid) |
| NOP16 | CERK | 0.72 | MI:0030(cross-linking study)\|bioid |
| NOTCH1 | PRKDC | 0.76 | MI:0004(affinity chromatography technology)\|MI:0676(tandem affinity purification)\|MI:0007(anti tag coimmunoprecipitation) |
| NOTCH1 | PRPF6 | 0.72 | MI:0004(affinity chromatography technology)\|MI:0676(tandem affinity purification) |
| NPHP1 | TNK2 | 0.84 | MI:0004(affinity chromatography technology)\|MI:0018(two hybrid)\|MI:0096(pull down) |
| NR1H4 | PRKDC | 0.75 | MI:0004(affinity chromatography technology)\|MI:0096(pull down)\|MI:0007(anti tag coimmunoprecipitation) |
| NUCB1 | PRKDC | 0.72 | MI:0004(affinity chromatography technology)\|MI:0401(biochemical) |
| OCIAD2 | SIK3 | 0.72 | MI:0030(cross-linking study)\|bioid |
| PARD3 | PRKCZ | 0.95 | Affinity Capture-Western\|MI:0004(affinity chromatography technology)\|MI:0007(anti tag coimmunoprecipitation) |
| PARD6A | PRKCZ | 0.99 | MI:0492(in vitro)\|MI:0493(in vivo)\|Reconstituted Complex\|MI:0007(anti tag coimmunoprecipitation)\|MI:0022(colocalization by immunostaining)\|MI:0018(two hybrid)\|MI:0004(affinity chromatography technology)\|MI:0428(imaging technique)\|MI:0096(pull down) |
| PARD6B | PRKCZ | 0.9 | MI:0492(in vitro)\|MI:0493(in vivo)\|Reconstituted Complex\|MI:0018(two hybrid)\|MI:0096(pull down)\|MI:0019(coimmunoprecipitation)\|MI:0007(anti tag coimmunoprecipitation)\|MI:0004(affinity chromatography technology) |
| PARD6G | PRKCZ | 0.95 | MI:0492(in vitro)\|MI:0018(two hybrid)\|MI:0007(anti tag coimmunoprecipitation)\|MI:0004(affinity chromatography technology)\|MI:0096(pull down) |
| PARP1 | CDK8 | 0.78 | MI:0004(affinity chromatography technology)\|MI:0096(pull down) |
| PAWR | PRKCZ | 0.89 | MI:0492(in vitro)\|MI:0493(in vivo)\|MI:0018(two hybrid)\|Reconstituted Complex\|Affinity Capture-Western\|MI:0004(affinity chromatography technology)\|MI:0096(pull down) |
| PCBP1 | MET | 0.83 | MI:0004(affinity chromatography technology)\|MI:0007(anti tag coimmunoprecipitation) |
| PDCD10 | STK24 | 0.9 | Affinity Capture-MS\|MI:0007(anti tag coimmunoprecipitation)\|MI:0018(two hybrid)\|MI:0004(affinity chromatography technology)\|MI:0006(anti bait coimmunoprecipitation)\|MI:0676(tandem affinity purification)\|MI:0096(pull down)\|MI:0401(biochemical) |
| PDHB | PDK2 | 0.83 | MI:0004(affinity chromatography technology)\|MI:0007(anti tag coimmunoprecipitation) |
| PDK2 | PDK1 | 0.9 | MI:0492(in vitro)\|Reconstituted Complex\|MI:0096(pull down)\|MI:0004(affinity chromatography technology)\|MI:0007(anti tag coimmunoprecipitation)\|bioid |
| PDK2 | PDHX | 0.86 | MI:0492(in vitro)\|Reconstituted Complex\|MI:0096(pull down) |
| PDK2 | PDHA1 | 0.86 | MI:0492(in vitro)\|MI:0424(protein kinase assay)\|bioid |
| PDK2 | PDK2 | 0.7 | MI:0492(in vitro) |
| PDK3 | PDK2 | 0.85 | MI:0004(affinity chromatography technology)\|MI:0096(pull down)\|MI:0007(anti tag coimmunoprecipitation) |
| PDPK1 | PRKCZ | 0.9 | MI:0492(in vitro)\|MI:0493(in vivo)\|MI:0018(two hybrid)\|Affinity Capture-Western\|Biochemical Activity\|Reconstituted Complex\|MI:0415(enzymatic study)\|MI:0004(affinity chromatography technology)\|MI:0096(pull down) |
| PELP1 | HGS | 0.76 | MI:0492(in vitro)\|MI:0493(in vivo)\|MI:0018(two hybrid)\|MI:0004(affinity chromatography technology)\|MI:0096(pull down) |
| PFKFB3 | PFKFB4 | 0.82 | MI:0004(affinity chromatography technology)\|MI:0007(anti tag coimmunoprecipitation) |
| PFKFB4 | PFKFB1 | 0.82 | MI:0018(two hybrid)\|MI:0004(affinity chromatography technology) |
| PHKG2 | PRKDC | 0.72 | MI:0676(tandem affinity purification)\|MI:0004(affinity chromatography technology) |
| PIAS1 | PTK2 | 0.79 | MI:0492(in vitro)\|MI:0493(in vivo)\|MI:0018(two hybrid)\|MI:0428(imaging technique) |
| PIK3R2 | MET | 0.75 | MI:0053(fluorescence polarization spectroscopy)\|MI:0096(pull down) |
| PINK1 | PRKDC | 0.73 | MI:0004(affinity chromatography technology) |
| PKD1 | PTK2 | 0.77 | MI:0492(in vitro)\|MI:0493(in vivo)\|MI:0004(affinity chromatography technology) |
| PLK1 | PRKDC | 0.78 | MI:0096(pull down)\|MI:0004(affinity chromatography technology) |
| PLXNB1 | MET | 0.92 | MI:0492(in vitro)\|MI:0493(in vivo)\|MI:0007(anti tag coimmunoprecipitation)\|MI:0006(anti bait coimmunoprecipitation) |
| PLXNB2 | MET | 0.72 | MI:0007(anti tag coimmunoprecipitation)\|MI:0006(anti bait coimmunoprecipitation) |
| PMEPA1 | HGS | 0.75 | MI:0004(affinity chromatography technology)\|MI:0096(pull down)\|MI:0018(two hybrid) |
| PMS2 | PRKDC | 0.72 | Affinity Capture-MS\|MI:0004(affinity chromatography technology) |
| POLR2A | CDK8 | 0.98 | MI:0492(in vitro)\|MI:0493(in vivo)\|Co-fractionation\|Affinity Capture-Western\|MI:0004(affinity chromatography technology)\|MI:0415(enzymatic study)\|MI:0401(biochemical) |
| POT1 | PRKDC | 0.72 | MI:0004(affinity chromatography technology)\|MI:0007(anti tag coimmunoprecipitation) |
| PPP1CB | PTK2 | 0.92 | MI:0492(in vitro)\|MI:0493(in vivo)\|Affinity Capture-Western\|MI:0004(affinity chromatography technology) |
| PPP3CA | SIK3 | 0.83 | MI:0004(affinity chromatography technology)\|MI:0007(anti tag coimmunoprecipitation) |
| PPP3CC | SIK3 | 0.83 | MI:0004(affinity chromatography technology)\|MI:0007(anti tag coimmunoprecipitation) |
| PRCP | STK24 | 0.72 | MI:0004(affinity chromatography technology)\|MI:0401(biochemical) |
| PRKAA2 | PRKDC | 0.78 | MI:0096(pull down)\|MI:0004(affinity chromatography technology) |
| PRKAB2 | PRKDC | 0.72 | MI:0007(anti tag coimmunoprecipitation)\|MI:0004(affinity chromatography technology) |
| PRKACA | SIK3 | 0.83 | MI:0018(two hybrid)\|MI:0004(affinity chromatography technology) |
| PRKCZ | AKT1 | 0.9 | MI:0492(in vitro)\|MI:0493(in vivo)\|Affinity Capture-Western\|MI:0006(anti bait coimmunoprecipitation)\|MI:0424(protein kinase assay)\|MI:0004(affinity chromatography technology)\|MI:0415(enzymatic study) |
| PRKCZ | YWHAB | 0.89 | MI:0492(in vitro)\|MI:0493(in vivo)\|Biochemical Activity\|Affinity Capture-Western\|MI:0415(enzymatic study)\|MI:0004(affinity chromatography technology)\|MI:0018(two hybrid) |
| PRKCZ | AKT3 | 0.89 | MI:0492(in vitro)\|MI:0493(in vivo)\|MI:0018(two hybrid)\|Affinity Capture-Western\|Biochemical Activity\|MI:0415(enzymatic study)\|MI:0004(affinity chromatography technology)\|MI:0428(imaging technique) |
| PRKCZ | RELA | 0.89 | MI:0492(in vitro)\|MI:0493(in vivo)\|Biochemical Activity\|Affinity Capture-Western\|MI:0045(experimental interaction detection)\|MI:0415(enzymatic study)\|MI:0004(affinity chromatography technology) |
| PRKCZ | PRKCI | 0.89 | MI:0019(coimmunoprecipitation)\|MI:0004(affinity chromatography technology)\|MI:0007(anti tag coimmunoprecipitation)\|MI:0006(anti bait coimmunoprecipitation)\|MI:0096(pull down) |
| PRKCZ | CSNK2B | 0.88 | MI:0492(in vitro)\|MI:0493(in vivo)\|Affinity Capture-Western\|MI:0004(affinity chromatography technology)\|MI:0007(anti tag coimmunoprecipitation) |
| PRKCZ | RAF1 | 0.88 | MI:0493(in vivo)\|Affinity Capture-Western\|Reconstituted Complex\|MI:0004(affinity chromatography technology)\|MI:0096(pull down) |
| PRKCZ | NUMB | 0.85 | MI:0492(in vitro)\|MI:0007(anti tag coimmunoprecipitation)\|MI:0059(gst pull down)\|MI:0424(protein kinase assay)\|MI:0096(pull down)\|MI:0415(enzymatic study) |
| PRKCZ | LLGL2 | 0.85 | MI:0004(affinity chromatography technology)\|MI:0007(anti tag coimmunoprecipitation)\|MI:0096(pull down) |
| PRKCZ | NIPSNAP2 | 0.85 | MI:0004(affinity chromatography technology)\|MI:0096(pull down)\|MI:0007(anti tag coimmunoprecipitation) |
| PRKCZ | CALU | 0.84 | MI:0096(pull down)\|MI:0007(anti tag coimmunoprecipitation)\|MI:0004(affinity chromatography technology) |
| PRKCZ | PSME3 | 0.84 | MI:0096(pull down)\|MI:0007(anti tag coimmunoprecipitation)\|MI:0004(affinity chromatography technology) |
| PRKCZ | HADHB | 0.84 | MI:0096(pull down)\|MI:0007(anti tag coimmunoprecipitation)\|MI:0004(affinity chromatography technology) |
| PRKCZ | HADHA | 0.84 | MI:0096(pull down)\|MI:0007(anti tag coimmunoprecipitation)\|MI:0004(affinity chromatography technology) |
| PRKCZ | IPO5 | 0.84 | MI:0096(pull down)\|MI:0007(anti tag coimmunoprecipitation)\|MI:0004(affinity chromatography technology) |
| PRKCZ | NCF1 | 0.83 | MI:0492(in vitro)\|MI:0415(enzymatic study)\|MI:0096(pull down)\|MI:0006(anti bait coimmunoprecipitation)\|MI:0416(fluorescence microscopy) |
| PRKCZ | HSP90AA1 | 0.83 | MI:0018(two hybrid)\|MI:0004(affinity chromatography technology) |
| PRKCZ | PIAS4 | 0.83 | MI:0018(two hybrid)\|MI:0004(affinity chromatography technology)\|MI:0415(enzymatic study) |
| PRKCZ | NPM1 | 0.83 | MI:0004(affinity chromatography technology)\|MI:0007(anti tag coimmunoprecipitation) |
| PRKCZ | NIPSNAP1 | 0.82 | MI:0004(affinity chromatography technology)\|MI:0007(anti tag coimmunoprecipitation) |
| PRKCZ | NPM3 | 0.82 | MI:0004(affinity chromatography technology)\|MI:0007(anti tag coimmunoprecipitation) |
| PRKCZ | KEAP1 | 0.82 | MI:0004(affinity chromatography technology)\|MI:0007(anti tag coimmunoprecipitation) |
| PRKCZ | CDC42BPB | 0.82 | MI:0004(affinity chromatography technology)\|MI:0007(anti tag coimmunoprecipitation) |
| PRKCZ | BAG2 | 0.82 | MI:0004(affinity chromatography technology)\|MI:0007(anti tag coimmunoprecipitation) |
| PRKCZ | SLC25A5 | 0.82 | MI:0004(affinity chromatography technology)\|MI:0007(anti tag coimmunoprecipitation) |
| PRKCZ | MRPL53 | 0.82 | MI:0004(affinity chromatography technology)\|MI:0007(anti tag coimmunoprecipitation) |
| PRKCZ | CDC37 | 0.82 | MI:0004(affinity chromatography technology)\|MI:0007(anti tag coimmunoprecipitation) |
| PRKCZ | WDR26 | 0.82 | MI:0004(affinity chromatography technology)\|MI:0007(anti tag coimmunoprecipitation) |
| PRKCZ | HSPB1 | 0.82 | MI:0004(affinity chromatography technology)\|MI:0007(anti tag coimmunoprecipitation) |
| PRKCZ | NAP1L1 | 0.82 | MI:0004(affinity chromatography technology)\|MI:0007(anti tag coimmunoprecipitation) |
| PRKCZ | TUBB6 | 0.82 | MI:0004(affinity chromatography technology)\|MI:0007(anti tag coimmunoprecipitation) |
| PRKCZ | SLC25A3 | 0.82 | MI:0004(affinity chromatography technology)\|MI:0007(anti tag coimmunoprecipitation) |
| PRKCZ | MRPL12 | 0.82 | MI:0004(affinity chromatography technology)\|MI:0007(anti tag coimmunoprecipitation) |
| PRKCZ | NCL | 0.79 | MI:0492(in vitro)\|MI:0493(in vivo)\|Biochemical Activity\|MI:0415(enzymatic study) |
| PRKCZ | YWHAQ | 0.79 | Affinity Capture-Western\|Reconstituted Complex\|Biochemical Activity\|MI:0415(enzymatic study)\|MI:0004(affinity chromatography technology)\|MI:0096(pull down) |
| PRKCZ | PRKCA | 0.78 | MI:0492(in vitro)\|MI:0493(in vivo)\|MI:0004(affinity chromatography technology) |
| PRKCZ | PRDX4 | 0.78 | MI:0004(affinity chromatography technology)\|MI:0096(pull down) |
| PRKCZ | EIF5A | 0.78 | MI:0004(affinity chromatography technology)\|MI:0096(pull down) |
| PRKCZ | PRKCQ | 0.77 | MI:0006(anti bait coimmunoprecipitation)\|MI:0018(two hybrid)\|MI:0424(protein kinase assay) |
| PRKCZ | PEBP1 | 0.73 | MI:0492(in vitro)\|MI:0493(in vivo)\|MI:0096(pull down) |
| PRKCZ | KRT10 | 0.73 | MI:0493(in vivo)\|Affinity Capture-Western\|MI:0004(affinity chromatography technology) |
| PRKCZ | CFL1 | 0.73 | MI:0004(affinity chromatography technology) |
| PRKCZ | RPS6KB1 | 0.72 | Affinity Capture-Western\|MI:0004(affinity chromatography technology) |
| PRKCZ | YWHAG | 0.72 | Affinity Capture-Western\|MI:0004(affinity chromatography technology) |
| PRKCZ | YWHAH | 0.72 | Affinity Capture-Western\|MI:0004(affinity chromatography technology) |
| PRKCZ | DYNLL1 | 0.72 | MI:0004(affinity chromatography technology)\|MI:0007(anti tag coimmunoprecipitation) |
| PRKCZ | AGER | 0.72 | MI:0007(anti tag coimmunoprecipitation)\|MI:0006(anti bait coimmunoprecipitation) |
| PRKCZ | GLG1 | 0.72 | MI:0007(anti tag coimmunoprecipitation)\|MI:0004(affinity chromatography technology) |
| PRKCZ | AP3S1 | 0.72 | MI:0007(anti tag coimmunoprecipitation)\|MI:0004(affinity chromatography technology) |
| PRKCZ | FAT1 | 0.72 | MI:0007(anti tag coimmunoprecipitation)\|MI:0004(affinity chromatography technology) |
| PRKCZ | RCN2 | 0.72 | MI:0007(anti tag coimmunoprecipitation)\|MI:0004(affinity chromatography technology) |
| PRKCZ | FBL | 0.72 | MI:0007(anti tag coimmunoprecipitation)\|MI:0004(affinity chromatography technology) |
| PRKCZ | UBE2O | 0.72 | MI:0007(anti tag coimmunoprecipitation)\|MI:0004(affinity chromatography technology) |
| PRKCZ | TRIM28 | 0.72 | MI:0007(anti tag coimmunoprecipitation)\|MI:0004(affinity chromatography technology) |
| PRKCZ | DDA1 | 0.72 | MI:0007(anti tag coimmunoprecipitation)\|MI:0004(affinity chromatography technology) |
| PRKCZ | AIFM1 | 0.72 | MI:0007(anti tag coimmunoprecipitation)\|MI:0004(affinity chromatography technology) |
| PRKCZ | LOC100290337 | 0.72 | MI:0007(anti tag coimmunoprecipitation)\|MI:0004(affinity chromatography technology) |
| PRKCZ | PRKACB | 0.72 | MI:0007(anti tag coimmunoprecipitation)\|MI:0004(affinity chromatography technology) |
| PRKCZ | NEDD8 | 0.72 | MI:0007(anti tag coimmunoprecipitation)\|MI:0004(affinity chromatography technology) |
| PRKCZ | RPUSD4 | 0.72 | MI:0007(anti tag coimmunoprecipitation)\|MI:0004(affinity chromatography technology) |
| PRKCZ | AP2M1 | 0.72 | MI:0007(anti tag coimmunoprecipitation)\|MI:0004(affinity chromatography technology) |
| PRKCZ | EPB41L4B | 0.72 | MI:0007(anti tag coimmunoprecipitation)\|MI:0004(affinity chromatography technology) |
| PRKCZ | SLC25A6 | 0.72 | MI:0007(anti tag coimmunoprecipitation)\|MI:0004(affinity chromatography technology) |
| PRKCZ | SLC25A4 | 0.72 | MI:0007(anti tag coimmunoprecipitation)\|MI:0004(affinity chromatography technology) |
| PRKCZ | PRKAR1A | 0.72 | MI:0007(anti tag coimmunoprecipitation)\|MI:0004(affinity chromatography technology) |
| PRKCZ | MRPS2 | 0.72 | MI:0007(anti tag coimmunoprecipitation)\|MI:0004(affinity chromatography technology) |
| PRKCZ | DCAF1 | 0.72 | MI:0007(anti tag coimmunoprecipitation)\|MI:0004(affinity chromatography technology) |
| PRKCZ | NOC2L | 0.72 | MI:0007(anti tag coimmunoprecipitation)\|MI:0004(affinity chromatography technology) |
| PRKCZ | GTF3C5 | 0.72 | MI:0007(anti tag coimmunoprecipitation)\|MI:0004(affinity chromatography technology) |
| PRKCZ | HSPA8 | 0.72 | MI:0007(anti tag coimmunoprecipitation)\|MI:0004(affinity chromatography technology) |
| PRKCZ | CPT1A | 0.72 | MI:0007(anti tag coimmunoprecipitation)\|MI:0004(affinity chromatography technology) |
| PRKCZ | DNAJA2 | 0.72 | MI:0007(anti tag coimmunoprecipitation)\|MI:0004(affinity chromatography technology) |
| PRKCZ | POLD1 | 0.72 | MI:0007(anti tag coimmunoprecipitation)\|MI:0004(affinity chromatography technology) |
| PRKCZ | NAP1L4 | 0.72 | MI:0007(anti tag coimmunoprecipitation)\|MI:0004(affinity chromatography technology) |
| PRKCZ | ADH1C | 0.72 | MI:0007(anti tag coimmunoprecipitation)\|MI:0004(affinity chromatography technology) |
| PRKCZ | CAD | 0.72 | MI:0007(anti tag coimmunoprecipitation)\|MI:0004(affinity chromatography technology) |
| PRKCZ | PPFIBP1 | 0.72 | MI:0007(anti tag coimmunoprecipitation)\|MI:0004(affinity chromatography technology) |
| PRKCZ | TUBB4B | 0.72 | MI:0007(anti tag coimmunoprecipitation)\|MI:0004(affinity chromatography technology) |
| PRKCZ | CSNK2A1 | 0.72 | MI:0007(anti tag coimmunoprecipitation)\|MI:0004(affinity chromatography technology) |
| PRKCZ | ITSN2 | 0.72 | MI:0007(anti tag coimmunoprecipitation)\|MI:0004(affinity chromatography technology) |
| PRKCZ | HSPA6 | 0.72 | MI:0007(anti tag coimmunoprecipitation)\|MI:0004(affinity chromatography technology) |
| PRKCZ | HUWE1 | 0.72 | MI:0007(anti tag coimmunoprecipitation)\|MI:0004(affinity chromatography technology) |
| PRKCZ | DNAJA1 | 0.72 | MI:0007(anti tag coimmunoprecipitation)\|MI:0004(affinity chromatography technology) |
| PRKCZ | CAMSAP3 | 0.72 | MI:0007(anti tag coimmunoprecipitation)\|MI:0004(affinity chromatography technology) |
| PRKCZ | TIMM50 | 0.72 | MI:0007(anti tag coimmunoprecipitation)\|MI:0004(affinity chromatography technology) |
| PRKCZ | AP2A2 | 0.72 | MI:0007(anti tag coimmunoprecipitation)\|MI:0004(affinity chromatography technology) |
| PRKCZ | ASPH | 0.72 | MI:0007(anti tag coimmunoprecipitation)\|MI:0004(affinity chromatography technology) |
| PRKCZ | TRIM26 | 0.72 | MI:0007(anti tag coimmunoprecipitation)\|MI:0004(affinity chromatography technology) |
| PRKCZ | FARP2 | 0.72 | MI:0007(anti tag coimmunoprecipitation)\|MI:0004(affinity chromatography technology) |
| PRKCZ | HSPA7 | 0.72 | MI:0007(anti tag coimmunoprecipitation)\|MI:0004(affinity chromatography technology) |
| PRKCZ | POLRMT | 0.72 | MI:0007(anti tag coimmunoprecipitation)\|MI:0004(affinity chromatography technology) |
| PRKCZ | TUBB | 0.72 | MI:0007(anti tag coimmunoprecipitation)\|MI:0004(affinity chromatography technology) |
| PRKCZ | BLVRA | 0.7 | MI:0004(affinity chromatography technology)\|MI:0096(pull down)\|MI:0415(enzymatic study) |
| PRKCZ | MAP1LC3B | 0.7 | MI:0004(affinity chromatography technology)\|MI:0096(pull down)\|MI:0415(enzymatic study) |
| PRKDC | RPA2 | 0.9 | MI:0492(in vitro)\|Affinity Capture-Western\|Reconstituted Complex\|MI:0019(coimmunoprecipitation)\|MI:0004(affinity chromatography technology)\|MI:0096(pull down)\|MI:0415(enzymatic study) |
| PRKDC | RPA1 | 0.89 | MI:0492(in vitro)\|Reconstituted Complex\|MI:0019(coimmunoprecipitation)\|MI:0096(pull down)\|MI:0004(affinity chromatography technology) |
| PRKDC | TP53 | 0.88 | MI:0492(in vitro)\|MI:0493(in vivo)\|Protein-peptide\|Biochemical Activity\|MI:0415(enzymatic study)\|MI:0004(affinity chromatography technology) |
| PRKDC | LIG4 | 0.88 | MI:0492(in vitro)\|Protein-peptide\|MI:0415(enzymatic study)\|MI:0004(affinity chromatography technology)\|MI:0006(anti bait coimmunoprecipitation) |
| PRKDC | CIB1 | 0.88 | MI:0492(in vitro)\|MI:0493(in vivo)\|MI:0018(two hybrid)\|Reconstituted Complex\|MI:0096(pull down)\|MI:0004(affinity chromatography technology) |
| PRKDC | ILF3 | 0.88 | MI:0025(copurification)\|Affinity Capture-Western\|MI:0004(affinity chromatography technology)\|MI:0401(biochemical) |
| PRKDC | ERG | 0.88 | MI:0096(pull down)\|MI:0402(chromatin immunoprecipitation assay)\|MI:0006(anti bait coimmunoprecipitation)\|MI:0007(anti tag coimmunoprecipitation)\|MI:0004(affinity chromatography technology) |
| PRKDC | CHEK1 | 0.87 | Protein-peptide\|Reconstituted Complex\|MI:0096(pull down) |
| PRKDC | PIDD1 | 0.87 | MI:0006(anti bait coimmunoprecipitation)\|MI:0007(anti tag coimmunoprecipitation)\|MI:0096(pull down)\|MI:0004(affinity chromatography technology) |
| PRKDC | AR | 0.86 | Affinity Capture-MS\|MI:0004(affinity chromatography technology)\|MI:0006(anti bait coimmunoprecipitation) |
| PRKDC | PPP6C | 0.85 | MI:0004(affinity chromatography technology)\|MI:0096(pull down)\|MI:0007(anti tag coimmunoprecipitation) |
| PRKDC | PPP6R2 | 0.85 | MI:0004(affinity chromatography technology)\|MI:0096(pull down)\|MI:0007(anti tag coimmunoprecipitation) |
| PRKDC | PARP1 | 0.84 | MI:0493(in vivo)\|MI:0006(anti bait coimmunoprecipitation)\|MI:0004(affinity chromatography technology) |
| PRKDC | BRCA1 | 0.84 | Protein-peptide\|MI:0004(affinity chromatography technology)\|MI:0096(pull down) |
| PRKDC | MAPK9 | 0.83 | Biochemical Activity\|MI:0415(enzymatic study)\|MI:0004(affinity chromatography technology) |
| PRKDC | MRE11 | 0.82 | Protein-peptide\|MI:0004(affinity chromatography technology) |
| PRKDC | PRPF8 | 0.82 | MI:0004(affinity chromatography technology)\|MI:0401(biochemical) |
| PRKDC | EFTUD2 | 0.82 | MI:0004(affinity chromatography technology)\|MI:0401(biochemical) |
| PRKDC | SRF | 0.79 | MI:0492(in vitro)\|MI:0493(in vivo)\|MI:0415(enzymatic study)\|MI:0007(anti tag coimmunoprecipitation) |
| PRKDC | CHUK | 0.78 | MI:0493(in vivo)\|Biochemical Activity\|Reconstituted Complex\|MI:0415(enzymatic study)\|MI:0096(pull down) |
| PRKDC | JUN | 0.77 | MI:0492(in vitro)\|MI:0415(enzymatic study)\|MI:0006(anti bait coimmunoprecipitation) |
| PRKDC | XPA | 0.77 | MI:0492(in vitro)\|MI:0415(enzymatic study)\|MI:0007(anti tag coimmunoprecipitation) |
| PRKDC | LYN | 0.75 | MI:0492(in vitro)\|Reconstituted Complex\|MI:0096(pull down) |
| PRKDC | PRKCD | 0.75 | MI:0492(in vitro)\|MI:0007(anti tag coimmunoprecipitation) |
| PRKDC | ABL1 | 0.75 | MI:0492(in vitro)\|Reconstituted Complex\|MI:0096(pull down) |
| PRKDC | HSP90AA1 | 0.75 | MI:0492(in vitro)\|MI:0004(affinity chromatography technology) |
| PRKDC | HSF1 | 0.75 | MI:0492(in vitro)\|Reconstituted Complex\|MI:0096(pull down) |
| PRKDC | AKT2 | 0.75 | Biochemical Activity\|MI:0415(enzymatic study) |
| PRKDC | PCNA | 0.73 | MI:0492(in vitro)\|Affinity Capture-MS\|MI:0004(affinity chromatography technology) |
| PRKDC | RBBP8 | 0.73 | MI:0004(affinity chromatography technology) |
| PRKDC | RUVBL2 | 0.73 | MI:0004(affinity chromatography technology) |
| PRKDC | MCM2 | 0.73 | MI:0004(affinity chromatography technology) |
| PRKDC | HIF1A | 0.73 | MI:0004(affinity chromatography technology) |
| PRKDC | XRCC1 | 0.73 | MI:0004(affinity chromatography technology) |
| PRKDC | AIRE | 0.72 | MI:0006(anti bait coimmunoprecipitation)\|MI:0004(affinity chromatography technology) |
| PRKDC | TOP2A | 0.72 | MI:0006(anti bait coimmunoprecipitation)\|MI:0007(anti tag coimmunoprecipitation) |
| PRKDC | PRDX1 | 0.72 | MI:0004(affinity chromatography technology)\|MI:0401(biochemical) |
| PRKDC | CTPS2 | 0.72 | MI:0004(affinity chromatography technology)\|MI:0401(biochemical) |
| PRKDC | GTF2I | 0.72 | MI:0004(affinity chromatography technology)\|MI:0401(biochemical) |
| PRKDC | NRXN1 | 0.72 | MI:0030(cross-linking study)\|bioid |
| PRKDC | GTPBP4 | 0.72 | MI:0030(cross-linking study)\|bioid |
| PRKDC | RNF8 | 0.72 | MI:0030(cross-linking study)\|bioid |
| PRKDC | TBC1D28 | 0.72 | MI:0030(cross-linking study)\|bioid |
| PRKDC | ZNF840P | 0.72 | MI:0030(cross-linking study)\|bioid |
| PRKDC | ANXA2 | 0.72 | MI:0030(cross-linking study)\|bioid |
| PRKDC | KIAA1217 | 0.72 | MI:0030(cross-linking study)\|bioid |
| PRKDC | LAMC3 | 0.72 | MI:0030(cross-linking study)\|bioid |
| PRKDC | CPN1 | 0.72 | MI:0030(cross-linking study)\|bioid |
| PRKDC | SYNE2 | 0.72 | MI:0030(cross-linking study)\|bioid |
| PRKDC | FAM161A | 0.72 | MI:0030(cross-linking study)\|bioid |
| PRKDC | SYNJ2 | 0.72 | MI:0030(cross-linking study)\|bioid |
| PRKDC | PWWP2B | 0.72 | MI:0030(cross-linking study)\|bioid |
| PRKG1 | PTK2 | 0.82 | MI:0004(affinity chromatography technology)\|MI:0007(anti tag coimmunoprecipitation) |
| PRMT1 | SPEG | 0.73 | MI:0018(two hybrid) |
| PRMT5 | FAM47E | 0.78 | MI:0006(anti bait coimmunoprecipitation)\|MI:0096(pull down)\|MI:0007(anti tag coimmunoprecipitation)\|MI:0018(two hybrid)\|MI:0004(affinity chromatography technology) |
| PRPF19 | PRPF6 | 0.73 | MI:0401(biochemical) |
| PRPF4 | PRPF6 | 0.98 | Affinity Capture-MS\|MI:0007(anti tag coimmunoprecipitation)\|MI:0004(affinity chromatography technology)\|MI:0401(biochemical) |
| PRPF4B | PRPF6 | 0.72 | MI:0676(tandem affinity purification)\|MI:0004(affinity chromatography technology) |
| PRPF6 | PRPF31 | 0.99 | MI:0018(two hybrid)\|MI:0007(anti tag coimmunoprecipitation)\|MI:0004(affinity chromatography technology)\|MI:0096(pull down)\|MI:0401(biochemical) |
| PRPF6 | SNRNP200 | 0.99 | MI:0004(affinity chromatography technology)\|MI:0018(two hybrid)\|MI:0676(tandem affinity purification)\|MI:0096(pull down)\|MI:0401(biochemical) |
| PRPF6 | PRPF8 | 0.99 | MI:0004(affinity chromatography technology)\|MI:0018(two hybrid)\|MI:0676(tandem affinity purification)\|MI:0096(pull down)\|bioid\|MI:0401(biochemical) |
| PRPF6 | EFTUD2 | 0.89 | MI:0018(two hybrid)\|MI:0004(affinity chromatography technology)\|MI:0676(tandem affinity purification)\|MI:0096(pull down)\|MI:0401(biochemical) |
| PRPF6 | AR | 0.88 | MI:0493(in vivo)\|Affinity Capture-Western\|Reconstituted Complex\|MI:0004(affinity chromatography technology)\|MI:0096(pull down) |
| PRPF6 | PRPF3 | 0.88 | MI:0004(affinity chromatography technology)\|MI:0096(pull down)\|MI:0007(anti tag coimmunoprecipitation)\|MI:0401(biochemical) |
| PRPF6 | SNRPD3 | 0.88 | MI:0004(affinity chromatography technology)\|MI:0401(biochemical) |
| PRPF6 | RPS24 | 0.82 | bioid\|MI:0401(biochemical) |
| PRPF6 | SNRPD1 | 0.82 | MI:0401(biochemical) |
| PRPF6 | SNRPD2 | 0.79 | MI:0401(biochemical) |
| PRPF6 | IK | 0.76 | MI:0018(two hybrid)\|MI:0007(anti tag coimmunoprecipitation)\|MI:0004(affinity chromatography technology) |
| PRPF6 | ARAF | 0.75 | MI:0492(in vitro)\|MI:0018(two hybrid) |
| PRPF6 | ESR1 | 0.75 | MI:0493(in vivo)\|MI:0004(affinity chromatography technology) |
| PRPF6 | SMU1 | 0.72 | MI:0004(affinity chromatography technology)\|MI:0676(tandem affinity purification) |
| PRPF6 | RIOK1 | 0.72 | MI:0004(affinity chromatography technology)\|MI:0676(tandem affinity purification) |
| PRPF6 | SF3B3 | 0.7 | MI:0401(biochemical) |
| PRPF6 | SNRPA1 | 0.7 | MI:0401(biochemical) |
| PRPF6 | SF3A3 | 0.7 | MI:0401(biochemical) |
| PTAFR | PTK2 | 0.72 | MI:0004(affinity chromatography technology)\|MI:0006(anti bait coimmunoprecipitation) |
| PTEN | PTK2 | 0.87 | Affinity Capture-Western\|MI:0004(affinity chromatography technology)\|MI:0096(pull down)\|MI:0415(enzymatic study)\|MI:0428(imaging technique) |
| PTK2 | GRB2 | 0.97 | MI:0492(in vitro)\|MI:0493(in vivo)\|Affinity Capture-Western\|Reconstituted Complex\|MI:0004(affinity chromatography technology)\|MI:0018(two hybrid)\|MI:0096(pull down)\|MI:0006(anti bait coimmunoprecipitation) |
| PTK2 | PXN | 0.97 | MI:0492(in vitro)\|MI:0493(in vivo)\|Affinity Capture-Western\|Reconstituted Complex\|MI:0096(pull down)\|MI:0007(anti tag coimmunoprecipitation)\|MI:0004(affinity chromatography technology)\|MI:0663(confocal microscopy)\|MI:0006(anti bait coimmunoprecipitation)\|MI:0047(far western blotting)\|MI:0416(fluorescence microscopy)\|bioid |
| PTK2 | BCAR1 | 0.97 | MI:0492(in vitro)\|MI:0493(in vivo)\|MI:0018(two hybrid)\|Affinity Capture-Western\|Reconstituted Complex\|MI:0049(filter binding)\|MI:0004(affinity chromatography technology)\|MI:0019(coimmunoprecipitation)\|MI:0096(pull down) |
| PTK2 | FYN | 0.97 | MI:0492(in vitro)\|Affinity Capture-Western\|Co-crystal Structure\|MI:0004(affinity chromatography technology)\|MI:0114(x-ray crystallography)\|MI:0096(pull down)\|MI:0399(two hybrid fragment pooling approach) |
| PTK2 | PTK2 | 0.96 | MI:0492(in vitro)\|MI:0493(in vivo)\|Co-crystal Structure\|MI:0114(x-ray crystallography)\|MI:0096(pull down)\|MI:0415(enzymatic study) |
| PTK2 | DCC | 0.96 | MI:0492(in vitro)\|MI:0493(in vivo)\|Reconstituted Complex\|Affinity Capture-Western\|MI:0018(two hybrid)\|MI:0004(affinity chromatography technology)\|MI:0006(anti bait coimmunoprecipitation)\|MI:0096(pull down) |
| PTK2 | PLCG1 | 0.92 | MI:0492(in vitro)\|MI:0493(in vivo)\|Affinity Capture-Western\|MI:0004(affinity chromatography technology)\|MI:0428(imaging technique) |
| PTK2 | SHC1 | 0.92 | MI:0492(in vitro)\|MI:0493(in vivo)\|Affinity Capture-Western\|MI:0004(affinity chromatography technology) |
| PTK2 | ITGB3 | 0.9 | MI:0492(in vitro)\|MI:0493(in vivo)\|Reconstituted Complex\|Affinity Capture-Western\|MI:0004(affinity chromatography technology)\|MI:0096(pull down) |
| PTK2 | STAT1 | 0.9 | MI:0492(in vitro)\|MI:0493(in vivo)\|Affinity Capture-Western\|Reconstituted Complex\|MI:0004(affinity chromatography technology)\|MI:0096(pull down)\|MI:0428(imaging technique)\|MI:0006(anti bait coimmunoprecipitation) |
| PTK2 | TP53 | 0.9 | MI:0492(in vitro)\|MI:0493(in vivo)\|Affinity Capture-Western\|Reconstituted Complex\|MI:0006(anti bait coimmunoprecipitation)\|MI:0663(confocal microscopy)\|MI:0096(pull down)\|MI:0004(affinity chromatography technology)\|MI:0428(imaging technique)\|MI:0401(biochemical) |
| PTK2 | EGFR | 0.9 | MI:0492(in vitro)\|MI:0493(in vivo)\|Affinity Capture-Western\|MI:0006(anti bait coimmunoprecipitation)\|MI:0004(affinity chromatography technology)\|MI:0018(two hybrid)\|MI:0112(ubiquitin reconstruction)\|MI:0090(protein complementation assay) |
| PTK2 | LYN | 0.89 | MI:0493(in vivo)\|Affinity Capture-Western\|MI:0019(coimmunoprecipitation)\|MI:0004(affinity chromatography technology)\|MI:0018(two hybrid) |
| PTK2 | TLN1 | 0.89 | MI:0492(in vitro)\|MI:0493(in vivo)\|Affinity Capture-Western\|Reconstituted Complex\|MI:0004(affinity chromatography technology)\|MI:0096(pull down) |
| PTK2 | PIK3R1 | 0.88 | MI:0493(in vivo)\|Affinity Capture-Western\|MI:0047(far western blotting)\|MI:0004(affinity chromatography technology) |
| PTK2 | PTPN11 | 0.88 | MI:0492(in vitro)\|MI:0493(in vivo)\|Affinity Capture-Western\|MI:0004(affinity chromatography technology)\|MI:0006(anti bait coimmunoprecipitation) |
| PTK2 | BMX | 0.88 | MI:0492(in vitro)\|MI:0493(in vivo)\|Affinity Capture-Western\|Reconstituted Complex\|MI:0004(affinity chromatography technology)\|MI:0096(pull down) |
| PTK2 | YES1 | 0.88 | Affinity Capture-Western\|MI:0004(affinity chromatography technology)\|MI:0018(two hybrid)\|MI:0428(imaging technique) |
| PTK2 | SOCS3 | 0.88 | MI:0004(affinity chromatography technology)\|MI:0018(two hybrid)\|MI:0096(pull down)\|MI:0047(far western blotting) |
| PTK2 | PIK3R3 | 0.87 | MI:0006(anti bait coimmunoprecipitation)\|MI:0004(affinity chromatography technology)\|MI:0018(two hybrid) |
| PTK2 | EZR | 0.86 | MI:0492(in vitro)\|MI:0493(in vivo)\|Reconstituted Complex\|MI:0096(pull down) |
| PTK2 | ERBB2 | 0.85 | MI:0493(in vivo)\|MI:0019(coimmunoprecipitation)\|MI:0428(imaging technique)\|MI:0004(affinity chromatography technology) |
| PTK2 | SYK | 0.84 | MI:0493(in vivo)\|Affinity Capture-Western\|MI:0004(affinity chromatography technology) |
| PTK2 | CSK | 0.83 | MI:0492(in vitro)\|MI:0493(in vivo)\|Affinity Capture-Western\|MI:0004(affinity chromatography technology) |
| PTK2 | NEO1 | 0.83 | MI:0492(in vitro)\|Affinity Capture-Western\|MI:0018(two hybrid)\|MI:0004(affinity chromatography technology) |
| PTK2 | JAK2 | 0.83 | MI:0493(in vivo)\|Affinity Capture-Western\|MI:0004(affinity chromatography technology) |
| PTK2 | STAT3 | 0.83 | Affinity Capture-Western\|MI:0004(affinity chromatography technology)\|MI:0428(imaging technique) |
| PTK2 | SRPK1 | 0.83 | MI:0424(protein kinase assay)\|MI:0415(enzymatic study)\|MI:0096(pull down) |
| PTK2 | PIK3CA | 0.82 | MI:0006(anti bait coimmunoprecipitation)\|MI:0004(affinity chromatography technology) |
| PTK2 | SWAP70 | 0.82 | MI:0004(affinity chromatography technology)\|bioid |
| PTK2 | PTPN23 | 0.79 | MI:0434(phosphatase assay)\|MI:0051(fluorescence technology)\|MI:0424(protein kinase assay)\|MI:0096(pull down)\|MI:0006(anti bait coimmunoprecipitation) |
| PTK2 | ITGAV | 0.78 | MI:0492(in vitro)\|MI:0004(affinity chromatography technology)\|MI:0428(imaging technique) |
| PTK2 | ITGB1 | 0.78 | MI:0492(in vitro)\|MI:0004(affinity chromatography technology)\|MI:0428(imaging technique) |
| PTK2 | EPB41L5 | 0.78 | MI:0096(pull down)\|bioid |
| PTK2 | EPHA2 | 0.77 | MI:0493(in vivo)\|Affinity Capture-Western\|MI:0004(affinity chromatography technology)\|MI:0416(fluorescence microscopy)\|MI:0006(anti bait coimmunoprecipitation) |
| PTK2 | IRS1 | 0.76 | MI:0018(two hybrid)\|Affinity Capture-Western\|MI:0004(affinity chromatography technology) |
| PTK2 | SELE | 0.75 | MI:0492(in vitro)\|Affinity Capture-Western\|MI:0004(affinity chromatography technology)\|MI:0096(pull down) |
| PTK2 | MDM2 | 0.75 | Reconstituted Complex\|MI:0096(pull down) |
| PTK2 | APC | 0.75 | MI:0428(imaging technique)\|bioid |
| PTK2 | FGR | 0.73 | MI:0493(in vivo)\|Affinity Capture-Western\|MI:0004(affinity chromatography technology) |
| PTK2 | DEF6 | 0.73 | MI:0018(two hybrid) |
| PTK2 | TRIM72 | 0.73 | MI:0004(affinity chromatography technology) |
| PTK2 | DOCK1 | 0.72 | Affinity Capture-Western\|MI:0004(affinity chromatography technology) |
| PTK2 | PCSK1 | 0.72 | Affinity Capture-Western\|MI:0004(affinity chromatography technology) |
| PTK2 | TSC1 | 0.72 | Affinity Capture-Western\|MI:0004(affinity chromatography technology) |
| PTK2 | ITGA4 | 0.72 | Affinity Capture-Western\|MI:0004(affinity chromatography technology) |
| PTK2 | AMPH | 0.72 | Affinity Capture-Western\|MI:0004(affinity chromatography technology) |
| PTK2 | SH2D2A | 0.72 | MI:0004(affinity chromatography technology)\|MI:0018(two hybrid) |
| PTK6 | MET | 0.75 | MI:0053(fluorescence polarization spectroscopy)\|MI:0096(pull down) |
| PTPN1 | PTK2 | 0.75 | MI:0434(phosphatase assay) |
| PTPN11 | MET | 0.88 | MI:0493(in vivo)\|MI:0006(anti bait coimmunoprecipitation)\|MI:0053(fluorescence polarization spectroscopy)\|MI:0096(pull down) |
| PTPN12 | PTK2 | 0.78 | MI:0492(in vitro)\|MI:0493(in vivo)\|bioid |
| PTPN12 | PRKDC | 0.72 | MI:0007(anti tag coimmunoprecipitation)\|MI:0004(affinity chromatography technology) |
| PTPN23 | HGS | 0.73 | MI:0004(affinity chromatography technology) |
| PTPN6 | PTK2 | 0.72 | MI:0004(affinity chromatography technology)\|MI:0018(two hybrid) |
| PTPRB | MET | 0.7 | MI:0492(in vitro)\|MI:0434(phosphatase assay) |
| PTPRJ | MET | 0.89 | MI:0492(in vitro)\|MI:0493(in vivo)\|MI:0434(phosphatase assay)\|MI:0004(affinity chromatography technology)\|MI:0019(coimmunoprecipitation)\|MI:0096(pull down) |
| PTPRO | MET | 0.86 | MI:0434(phosphatase assay)\|MI:0004(affinity chromatography technology)\|MI:0096(pull down) |
| PTRH2 | PTK2 | 0.72 | MI:0006(anti bait coimmunoprecipitation)\|MI:0004(affinity chromatography technology) |
| RAB4B | PRPF6 | 0.72 | MI:0004(affinity chromatography technology)\|MI:0007(anti tag coimmunoprecipitation) |
| RAB5A | PRKDC | 0.72 | MI:0007(anti tag coimmunoprecipitation)\|MI:0004(affinity chromatography technology) |
| RAB5A | PTK2 | 0.72 | MI:0007(anti tag coimmunoprecipitation)\|MI:0004(affinity chromatography technology) |
| RAC1 | KALRN | 0.87 | Reconstituted Complex\|MI:0096(pull down)\|MI:0004(affinity chromatography technology) |
| RAD21 | PRKDC | 0.82 | MI:0004(affinity chromatography technology)\|MI:0401(biochemical) |
| RAF1 | MET | 0.75 | MI:0055(fluorescent resonance energy transfer) |
| RANBP10 | MET | 0.79 | MI:0492(in vitro)\|MI:0493(in vivo)\|Affinity Capture-Western\|Reconstituted Complex\|MI:0004(affinity chromatography technology)\|MI:0096(pull down) |
| RANBP9 | MET | 0.79 | MI:0492(in vitro)\|MI:0493(in vivo)\|Reconstituted Complex\|Affinity Capture-Western\|MI:0018(two hybrid)\|MI:0019(coimmunoprecipitation)\|MI:0096(pull down)\|MI:0004(affinity chromatography technology) |
| RASSF1 | MET | 0.75 | MI:0055(fluorescent resonance energy transfer) |
| RB1CC1 | PTK2 | 0.79 | MI:0492(in vitro)\|MI:0493(in vivo)\|MI:0018(two hybrid)\|Reconstituted Complex\|Affinity Capture-Western\|MI:0004(affinity chromatography technology)\|MI:0096(pull down) |
| RBM42 | PRPF6 | 0.72 | MI:0004(affinity chromatography technology)\|MI:0007(anti tag coimmunoprecipitation) |
| RECQL5 | PRKDC | 0.72 | Affinity Capture-MS\|MI:0004(affinity chromatography technology) |
| RELA | PRKDC | 0.86 | MI:0676(tandem affinity purification)\|MI:0007(anti tag coimmunoprecipitation)\|MI:0004(affinity chromatography technology) |
| RELB | PRKDC | 0.72 | MI:0676(tandem affinity purification)\|MI:0007(anti tag coimmunoprecipitation) |
| RFC2 | PRKDC | 0.72 | MI:0004(affinity chromatography technology)\|MI:0401(biochemical) |
| RHOA | PRKDC | 0.72 | MI:0007(anti tag coimmunoprecipitation)\|MI:0004(affinity chromatography technology) |
| RIPK3 | PRKDC | 0.72 | MI:0676(tandem affinity purification)\|MI:0007(anti tag coimmunoprecipitation) |
| RIPK4 | PRKDC | 0.72 | MI:0004(affinity chromatography technology)\|MI:0007(anti tag coimmunoprecipitation) |
| RNF144A | PRKDC | 0.81 | MI:0415(enzymatic study)\|MI:0004(affinity chromatography technology)\|MI:0096(pull down) |
| RNF144B | PRKDC | 0.7 | MI:0415(enzymatic study)\|MI:0004(affinity chromatography technology)\|MI:0096(pull down) |
| RNF4 | PRKDC | 0.73 | MI:0004(affinity chromatography technology) |
| RNPS1 | PRPF6 | 0.88 | Affinity Capture-MS\|MI:0006(anti bait coimmunoprecipitation)\|MI:0004(affinity chromatography technology)\|MI:0007(anti tag coimmunoprecipitation) |
| RNU11 | PRPF6 | 0.72 | Affinity Capture-MS\|MI:0004(affinity chromatography technology) |
| RNU12-2P | PRPF6 | 0.72 | Affinity Capture-MS\|MI:0004(affinity chromatography technology) |
| RPS14 | CERK | 0.72 | MI:0030(cross-linking study)\|bioid |
| RTCB | PRKDC | 0.72 | MI:0004(affinity chromatography technology)\|MI:0401(biochemical) |
| RTRAF | PRKDC | 0.72 | MI:0004(affinity chromatography technology)\|MI:0401(biochemical) |
| RUVBL1 | PRKDC | 0.78 | MI:0096(pull down)\|MI:0004(affinity chromatography technology) |
| SART1 | PRPF6 | 0.89 | MI:0004(affinity chromatography technology)\|MI:0018(two hybrid)\|MI:0096(pull down)\|MI:0006(anti bait coimmunoprecipitation)\|MI:0401(biochemical) |
| SCGB1D1 | MET | 0.82 | MI:0004(affinity chromatography technology)\|MI:0007(anti tag coimmunoprecipitation) |
| SERPINB2 | PTK2 | 0.82 | MI:0004(affinity chromatography technology)\|MI:0007(anti tag coimmunoprecipitation) |
| SF3A3 | HGS | 0.72 | MI:0004(affinity chromatography technology)\|MI:0401(biochemical) |
| SH2B2 | MET | 0.75 | MI:0053(fluorescence polarization spectroscopy)\|MI:0096(pull down) |
| SH2B3 | MET | 0.75 | MI:0053(fluorescence polarization spectroscopy)\|MI:0096(pull down) |
| SH2D1A | MET | 0.75 | MI:0053(fluorescence polarization spectroscopy)\|MI:0096(pull down) |
| SH2D1A | TNK2 | 0.73 | MI:0018(two hybrid) |
| SH2D1B | MET | 0.75 | MI:0053(fluorescence polarization spectroscopy)\|MI:0096(pull down) |
| SH2D2A | MET | 0.75 | MI:0053(fluorescence polarization spectroscopy)\|MI:0096(pull down) |
| SH3KBP1 | MET | 0.72 | Affinity Capture-Western\|MI:0004(affinity chromatography technology) |
| SHB | MET | 0.75 | MI:0053(fluorescence polarization spectroscopy)\|MI:0096(pull down) |
| SHC1 | MET | 0.89 | MI:0493(in vivo)\|Affinity Capture-Western\|MI:0004(affinity chromatography technology)\|MI:0053(fluorescence polarization spectroscopy)\|MI:0096(pull down) |
| SHC1 | PRKDC | 0.7 | MI:0004(affinity chromatography technology) |
| SHC2 | MET | 0.75 | MI:0053(fluorescence polarization spectroscopy)\|MI:0096(pull down) |
| SHC4 | MET | 0.75 | MI:0053(fluorescence polarization spectroscopy)\|MI:0096(pull down) |
| SIK1 | SIK3 | 0.82 | MI:0004(affinity chromatography technology)\|MI:0007(anti tag coimmunoprecipitation) |
| SIK3 | YWHAH | 0.83 | MI:0004(affinity chromatography technology)\|MI:0007(anti tag coimmunoprecipitation) |
| SIK3 | YWHAE | 0.82 | MI:0676(tandem affinity purification)\|MI:0004(affinity chromatography technology) |
| SIK3 | TRIP6 | 0.73 | MI:0018(two hybrid) |
| SIK3 | CRTC2 | 0.71 | MI:0492(in vitro)\|MI:0493(in vivo)\|MI:0424(protein kinase assay) |
| SIKE1 | STK24 | 0.89 | Affinity Capture-MS\|MI:0007(anti tag coimmunoprecipitation)\|MI:0004(affinity chromatography technology)\|MI:0676(tandem affinity purification)\|MI:0096(pull down) |
| SIRT6 | PRKDC | 0.75 | MI:0004(affinity chromatography technology)\|MI:0428(imaging technique) |
| SLA2 | MET | 0.75 | MI:0053(fluorescence polarization spectroscopy)\|MI:0096(pull down) |
| SLK | HGS | 0.72 | MI:0004(affinity chromatography technology)\|MI:0401(biochemical) |
| SMAD2 | SIK3 | 0.72 | MI:0676(tandem affinity purification)\|MI:0004(affinity chromatography technology) |
| SMAD3 | SIK3 | 0.72 | MI:0676(tandem affinity purification)\|MI:0004(affinity chromatography technology) |
| SMAD4 | HGS | 0.73 | MI:0004(affinity chromatography technology) |
| SMAD5 | HGS | 0.72 | MI:0018(two hybrid)\|MI:0004(affinity chromatography technology) |
| SMARCA2 | PRPF6 | 0.77 | MI:0493(in vivo)\|MI:0006(anti bait coimmunoprecipitation)\|MI:0019(coimmunoprecipitation)\|MI:0004(affinity chromatography technology) |
| SNAP25 | HGS | 0.88 | MI:0018(two hybrid)\|MI:0004(affinity chromatography technology) |
| SNCAIP | KALRN | 0.77 | MI:0007(anti tag coimmunoprecipitation)\|MI:0663(confocal microscopy)\|MI:0018(two hybrid)\|MI:0004(affinity chromatography technology) |
| SNRNP40 | PRPF6 | 0.89 | Affinity Capture-MS\|MI:0006(anti bait coimmunoprecipitation)\|MI:0004(affinity chromatography technology)\|MI:0007(anti tag coimmunoprecipitation)\|MI:0096(pull down) |
| SNRNP70 | PRPF6 | 0.82 | MI:0004(affinity chromatography technology)\|MI:0018(two hybrid) |
| SNRPA | PRPF6 | 0.82 | MI:0018(two hybrid)\|MI:0401(biochemical) |
| SNRPA | PRKDC | 0.72 | MI:0004(affinity chromatography technology)\|MI:0401(biochemical) |
| SNRPB | PRPF6 | 0.82 | MI:0006(anti bait coimmunoprecipitation)\|MI:0004(affinity chromatography technology) |
| SNRPE | PRPF6 | 0.91 | MI:0004(affinity chromatography technology)\|MI:0007(anti tag coimmunoprecipitation) |
| SNRPF | PRPF6 | 0.94 | MI:0004(affinity chromatography technology)\|MI:0007(anti tag coimmunoprecipitation)\|MI:0018(two hybrid) |
| SNRPG | PRPF6 | 0.79 | MI:0004(affinity chromatography technology) |
| SNRPN | PRPF6 | 0.82 | MI:0004(affinity chromatography technology)\|MI:0007(anti tag coimmunoprecipitation) |
| SNW1 | PRPF6 | 0.72 | MI:0006(anti bait coimmunoprecipitation)\|MI:0004(affinity chromatography technology) |
| SNW1 | PRKDC | 0.72 | MI:0006(anti bait coimmunoprecipitation)\|MI:0004(affinity chromatography technology) |
| SNX9 | TNK2 | 0.88 | MI:0492(in vitro)\|MI:0493(in vivo)\|MI:0081(peptide array)\|MI:0007(anti tag coimmunoprecipitation)\|MI:0004(affinity chromatography technology) |
| SOCS1 | MET | 0.87 | MI:0053(fluorescence polarization spectroscopy)\|MI:0004(affinity chromatography technology)\|MI:0096(pull down) |
| SOCS1 | PRKDC | 0.72 | MI:0007(anti tag coimmunoprecipitation)\|MI:0004(affinity chromatography technology) |
| SOCS3 | MET | 0.75 | MI:0053(fluorescence polarization spectroscopy)\|MI:0096(pull down) |
| SOCS6 | MET | 0.75 | MI:0053(fluorescence polarization spectroscopy)\|MI:0096(pull down) |
| SP1 | PRKCZ | 0.75 | MI:0415(enzymatic study)\|MI:0004(affinity chromatography technology) |
| SQSTM1 | PRKCZ | 0.97 | MI:0492(in vitro)\|MI:0493(in vivo)\|MI:0018(two hybrid)\|Affinity Capture-Western\|MI:0004(affinity chromatography technology)\|MI:0096(pull down)\|MI:0007(anti tag coimmunoprecipitation)\|MI:0428(imaging technique)\|bioid |
| SQSTM1 | HGS | 0.73 | bioid |
| SRC | PTK2 | 0.97 | MI:0492(in vitro)\|MI:0493(in vivo)\|Affinity Capture-Western\|Reconstituted Complex\|MI:0018(two hybrid)\|MI:0006(anti bait coimmunoprecipitation)\|MI:0019(coimmunoprecipitation)\|MI:0007(anti tag coimmunoprecipitation)\|MI:0004(affinity chromatography technology)\|MI:0096(pull down)\|MI:0428(imaging technique)\|MI:0415(enzymatic study) |
| SRC | MET | 0.9 | MI:0493(in vivo)\|Affinity Capture-Western\|MI:0004(affinity chromatography technology)\|MI:0006(anti bait coimmunoprecipitation)\|MI:0053(fluorescence polarization spectroscopy)\|MI:0096(pull down) |
| SRC | PRKCZ | 0.86 | MI:0493(in vivo)\|Affinity Capture-Western\|MI:0004(affinity chromatography technology)\|MI:0096(pull down) |
| SRC | TNK2 | 0.79 | MI:0096(pull down)\|MI:0004(affinity chromatography technology) |
| SREBF1 | CDK8 | 0.78 | MI:0096(pull down)\|MI:0004(affinity chromatography technology) |
| SRRM2 | PRPF6 | 0.82 | MI:0018(two hybrid)\|MI:0401(biochemical) |
| STAT5A | PDK2 | 0.82 | MI:0004(affinity chromatography technology)\|MI:0007(anti tag coimmunoprecipitation) |
| STK24 | STRN | 0.9 | Affinity Capture-MS\|Affinity Capture-Western\|MI:0007(anti tag coimmunoprecipitation)\|MI:0004(affinity chromatography technology)\|MI:0006(anti bait coimmunoprecipitation)\|MI:0676(tandem affinity purification)\|MI:0096(pull down)\|bioid |
| STK24 | STRN3 | 0.9 | Affinity Capture-MS\|MI:0007(anti tag coimmunoprecipitation)\|MI:0004(affinity chromatography technology)\|MI:0006(anti bait coimmunoprecipitation)\|MI:0676(tandem affinity purification)\|MI:0096(pull down)\|bioid |
| STK24 | PPP2R1A | 0.9 | Affinity Capture-MS\|MI:0007(anti tag coimmunoprecipitation)\|MI:0004(affinity chromatography technology)\|MI:0006(anti bait coimmunoprecipitation)\|MI:0676(tandem affinity purification)\|MI:0096(pull down) |
| STK24 | STRN4 | 0.9 | Affinity Capture-MS\|MI:0007(anti tag coimmunoprecipitation)\|MI:0004(affinity chromatography technology)\|MI:0006(anti bait coimmunoprecipitation)\|MI:0676(tandem affinity purification)\|MI:0096(pull down) |
| STK24 | STK25 | 0.9 | Affinity Capture-MS\|MI:0007(anti tag coimmunoprecipitation)\|MI:0004(affinity chromatography technology)\|MI:0006(anti bait coimmunoprecipitation)\|MI:0676(tandem affinity purification)\|MI:0096(pull down) |
| STK24 | SLMAP | 0.9 | Affinity Capture-MS\|MI:0007(anti tag coimmunoprecipitation)\|MI:0004(affinity chromatography technology)\|MI:0006(anti bait coimmunoprecipitation)\|MI:0676(tandem affinity purification)\|MI:0096(pull down) |
| STK24 | PPP2CB | 0.89 | Affinity Capture-MS\|MI:0004(affinity chromatography technology)\|MI:0006(anti bait coimmunoprecipitation)\|MI:0676(tandem affinity purification)\|MI:0096(pull down) |
| STK24 | PPP2CA | 0.89 | Affinity Capture-MS\|MI:0007(anti tag coimmunoprecipitation)\|MI:0004(affinity chromatography technology)\|MI:0676(tandem affinity purification)\|MI:0096(pull down) |
| STK24 | PPP2R1B | 0.89 | Affinity Capture-MS\|MI:0007(anti tag coimmunoprecipitation)\|MI:0004(affinity chromatography technology)\|MI:0676(tandem affinity purification)\|MI:0096(pull down) |
| STK24 | STRIP2 | 0.89 | Affinity Capture-MS\|MI:0007(anti tag coimmunoprecipitation)\|MI:0004(affinity chromatography technology)\|MI:0676(tandem affinity purification)\|MI:0096(pull down) |
| STK24 | CTTNBP2 | 0.89 | Affinity Capture-MS\|MI:0007(anti tag coimmunoprecipitation)\|MI:0004(affinity chromatography technology)\|MI:0676(tandem affinity purification)\|MI:0096(pull down) |
| STK24 | FGFR1OP2 | 0.89 | Affinity Capture-MS\|MI:0007(anti tag coimmunoprecipitation)\|MI:0004(affinity chromatography technology)\|MI:0676(tandem affinity purification)\|MI:0096(pull down) |
| STK24 | MOB4 | 0.89 | MI:0007(anti tag coimmunoprecipitation)\|MI:0006(anti bait coimmunoprecipitation)\|MI:0004(affinity chromatography technology)\|MI:0676(tandem affinity purification)\|MI:0096(pull down) |
| STK24 | STRIP1 | 0.88 | Affinity Capture-MS\|MI:0007(anti tag coimmunoprecipitation)\|MI:0004(affinity chromatography technology)\|MI:0096(pull down) |
| STK24 | STK26 | 0.88 | MI:0007(anti tag coimmunoprecipitation)\|MI:0676(tandem affinity purification)\|MI:0004(affinity chromatography technology)\|MI:0096(pull down) |
| STK24 | PROSER2 | 0.87 | MI:0676(tandem affinity purification)\|MI:0004(affinity chromatography technology)\|MI:0007(anti tag coimmunoprecipitation) |
| STK24 | HNRNPH3 | 0.86 | Affinity Capture-MS\|MI:0004(affinity chromatography technology)\|MI:0006(anti bait coimmunoprecipitation) |
| STK24 | CCT8 | 0.86 | Affinity Capture-MS\|MI:0007(anti tag coimmunoprecipitation)\|MI:0004(affinity chromatography technology) |
| STK24 | DYNLL1 | 0.84 | MI:0676(tandem affinity purification)\|MI:0004(affinity chromatography technology)\|MI:0096(pull down) |
| STK24 | EWSR1 | 0.82 | MI:0006(anti bait coimmunoprecipitation)\|MI:0004(affinity chromatography technology) |
| STK24 | TUBB | 0.82 | MI:0004(affinity chromatography technology)\|MI:0401(biochemical) |
| STK24 | PSMA5 | 0.78 | MI:0004(affinity chromatography technology)\|MI:0096(pull down) |
| STK24 | PSMD7 | 0.78 | MI:0004(affinity chromatography technology)\|MI:0096(pull down) |
| STK24 | PSMD13 | 0.78 | MI:0004(affinity chromatography technology)\|MI:0096(pull down) |
| STK24 | PSMD6 | 0.78 | MI:0004(affinity chromatography technology)\|MI:0096(pull down) |
| STK24 | MAP4K4 | 0.78 | MI:0096(pull down)\|MI:0004(affinity chromatography technology) |
| STK24 | APOD | 0.76 | Affinity Capture-MS\|MI:0004(affinity chromatography technology)\|MI:0006(anti bait coimmunoprecipitation) |
| STK24 | DDX5 | 0.76 | Affinity Capture-MS\|MI:0004(affinity chromatography technology)\|MI:0006(anti bait coimmunoprecipitation) |
| STK24 | G3BP2 | 0.76 | Affinity Capture-MS\|MI:0004(affinity chromatography technology)\|MI:0006(anti bait coimmunoprecipitation) |
| STK24 | PDCD6IP | 0.76 | Affinity Capture-MS\|MI:0004(affinity chromatography technology)\|MI:0006(anti bait coimmunoprecipitation) |
| STK24 | JPH3 | 0.76 | Affinity Capture-MS\|MI:0004(affinity chromatography technology)\|MI:0006(anti bait coimmunoprecipitation) |
| STK24 | SYNCRIP | 0.76 | Affinity Capture-MS\|MI:0004(affinity chromatography technology)\|MI:0006(anti bait coimmunoprecipitation) |
| STK24 | HNRNPA3 | 0.76 | Affinity Capture-MS\|MI:0004(affinity chromatography technology)\|MI:0006(anti bait coimmunoprecipitation) |
| STK24 | DSP | 0.76 | Affinity Capture-MS\|MI:0004(affinity chromatography technology)\|MI:0006(anti bait coimmunoprecipitation) |
| STK24 | CCT2 | 0.76 | Affinity Capture-MS\|MI:0007(anti tag coimmunoprecipitation)\|MI:0004(affinity chromatography technology) |
| STK24 | CCT3 | 0.76 | Affinity Capture-MS\|MI:0007(anti tag coimmunoprecipitation)\|MI:0004(affinity chromatography technology) |
| STK24 | TCP1 | 0.76 | Affinity Capture-MS\|MI:0007(anti tag coimmunoprecipitation)\|MI:0004(affinity chromatography technology) |
| STK24 | CCT5 | 0.76 | Affinity Capture-MS\|MI:0007(anti tag coimmunoprecipitation)\|MI:0004(affinity chromatography technology) |
| STK24 | CCT6A | 0.76 | Affinity Capture-MS\|MI:0007(anti tag coimmunoprecipitation)\|MI:0004(affinity chromatography technology) |
| STK24 | CCT7 | 0.76 | Affinity Capture-MS\|MI:0007(anti tag coimmunoprecipitation)\|MI:0004(affinity chromatography technology) |
| STK24 | CCT4 | 0.76 | Affinity Capture-MS\|MI:0007(anti tag coimmunoprecipitation)\|MI:0004(affinity chromatography technology) |
| STK24 | ZBTB24 | 0.72 | Affinity Capture-MS\|MI:0004(affinity chromatography technology) |
| STK24 | FABP5 | 0.72 | Affinity Capture-MS\|MI:0004(affinity chromatography technology) |
| STK24 | ALDH7A1 | 0.72 | MI:0004(affinity chromatography technology)\|MI:0401(biochemical) |
| STK24 | ASNS | 0.72 | MI:0004(affinity chromatography technology)\|MI:0401(biochemical) |
| STK24 | EIF5 | 0.72 | MI:0004(affinity chromatography technology)\|MI:0401(biochemical) |
| STK24 | ISOC1 | 0.72 | MI:0004(affinity chromatography technology)\|MI:0401(biochemical) |
| STK24 | SCPEP1 | 0.72 | MI:0004(affinity chromatography technology)\|MI:0401(biochemical) |
| STK24 | PPP1CA | 0.72 | MI:0004(affinity chromatography technology)\|MI:0401(biochemical) |
| STK24 | MAT2B | 0.72 | MI:0004(affinity chromatography technology)\|MI:0401(biochemical) |
| STK24 | MSN | 0.72 | MI:0004(affinity chromatography technology)\|MI:0401(biochemical) |
| STK24 | XPO1 | 0.72 | MI:0004(affinity chromatography technology)\|MI:0401(biochemical) |
| STK24 | PDIA4 | 0.72 | MI:0004(affinity chromatography technology)\|MI:0401(biochemical) |
| STK24 | PSMC4 | 0.7 | MI:0004(affinity chromatography technology) |
| STK24 | PSMD11 | 0.7 | MI:0004(affinity chromatography technology) |
| STK24 | CAB39 | 0.7 | MI:0018(two hybrid) |
| STK35 | HSP90AA1 | 0.85 | MI:0004(affinity chromatography technology)\|MI:0007(anti tag coimmunoprecipitation)\|MI:0096(pull down) |
| STK35 | HSP90AB1 | 0.85 | MI:0004(affinity chromatography technology)\|MI:0096(pull down)\|MI:0007(anti tag coimmunoprecipitation) |
| STK35 | C1orf174 | 0.85 | MI:0004(affinity chromatography technology)\|MI:0096(pull down)\|MI:0007(anti tag coimmunoprecipitation) |
| STK35 | CDC37 | 0.85 | MI:0004(affinity chromatography technology)\|MI:0096(pull down)\|MI:0007(anti tag coimmunoprecipitation) |
| STK35 | CTDSPL2 | 0.85 | MI:0004(affinity chromatography technology)\|MI:0096(pull down)\|MI:0007(anti tag coimmunoprecipitation) |
| STK35 | HSP90AB4P | 0.82 | MI:0004(affinity chromatography technology)\|MI:0007(anti tag coimmunoprecipitation) |
| STK35 | FKBP5 | 0.82 | MI:0004(affinity chromatography technology)\|MI:0007(anti tag coimmunoprecipitation) |
| STK35 | HSP90AA5P | 0.82 | MI:0004(affinity chromatography technology)\|MI:0007(anti tag coimmunoprecipitation) |
| STK35 | HSP90AA4P | 0.82 | MI:0004(affinity chromatography technology)\|MI:0007(anti tag coimmunoprecipitation) |
| STK35 | HSP90AB3P | 0.82 | MI:0004(affinity chromatography technology)\|MI:0007(anti tag coimmunoprecipitation) |
| STK35 | UBP1 | 0.82 | MI:0004(affinity chromatography technology)\|MI:0007(anti tag coimmunoprecipitation) |
| STK35 | TFCP2 | 0.82 | MI:0004(affinity chromatography technology)\|MI:0007(anti tag coimmunoprecipitation) |
| STK35 | PRR14L | 0.82 | MI:0004(affinity chromatography technology)\|MI:0007(anti tag coimmunoprecipitation) |
| STK35 | CDC37L1 | 0.82 | MI:0004(affinity chromatography technology)\|MI:0007(anti tag coimmunoprecipitation) |
| STK35 | PDLIM1 | 0.78 | MI:0492(in vitro)\|MI:0018(two hybrid)\|Reconstituted Complex\|MI:0096(pull down) |
| STUB1 | PRKCZ | 0.71 | MI:0415(enzymatic study)\|MI:0004(affinity chromatography technology)\|MI:0096(pull down)\|MI:0428(imaging technique) |
| SUMO2 | PRKDC | 0.79 | MI:0004(affinity chromatography technology)\|MI:0096(pull down) |
| SUMO2 | PRPF6 | 0.79 | MI:0004(affinity chromatography technology)\|MI:0096(pull down) |
| SYT12 | PDK2 | 0.82 | MI:0004(affinity chromatography technology)\|MI:0007(anti tag coimmunoprecipitation) |
| TADA2A | CDK8 | 0.88 | MI:0004(affinity chromatography technology)\|bioid |
| TADA2A | HGS | 0.74 | MI:0018(two hybrid) |
| TBCB | STK24 | 0.72 | MI:0004(affinity chromatography technology)\|MI:0401(biochemical) |
| TCEA1 | CDK8 | 0.94 | Reconstituted Complex\|MI:0096(pull down)\|MI:0401(biochemical) |
| TEC | MET | 0.75 | MI:0053(fluorescence polarization spectroscopy)\|MI:0096(pull down) |
| TELO2 | PRKDC | 0.87 | MI:0004(affinity chromatography technology)\|MI:0006(anti bait coimmunoprecipitation)\|MI:0676(tandem affinity purification) |
| TEX101 | HGS | 0.72 | MI:0006(anti bait coimmunoprecipitation)\|MI:0004(affinity chromatography technology) |
| TGFB1I1 | PTK2 | 0.97 | MI:0492(in vitro)\|MI:0493(in vivo)\|Reconstituted Complex\|Affinity Capture-Western\|MI:0006(anti bait coimmunoprecipitation)\|MI:0004(affinity chromatography technology)\|MI:0018(two hybrid)\|MI:0096(pull down) |
| THRA | CDK8 | 0.72 | Affinity Capture-Western\|MI:0004(affinity chromatography technology) |
| TIAM2 | PRKDC | 0.72 | MI:0030(cross-linking study)\|bioid |
| TINF2 | PRKDC | 0.72 | MI:0004(affinity chromatography technology)\|MI:0007(anti tag coimmunoprecipitation) |
| TMPRSS2 | MET | 0.72 | MI:0004(affinity chromatography technology)\|bioid |
| TMPRSS4 | MET | 0.72 | MI:0004(affinity chromatography technology)\|bioid |
| TNFRSF1A | PRKDC | 0.72 | MI:0676(tandem affinity purification)\|MI:0007(anti tag coimmunoprecipitation) |
| TNFRSF1B | PRKDC | 0.72 | MI:0676(tandem affinity purification)\|MI:0007(anti tag coimmunoprecipitation) |
| TNFRSF9 | CERK | 0.83 | MI:0004(affinity chromatography technology)\|MI:0007(anti tag coimmunoprecipitation) |
| TNK2 | GRB2 | 0.9 | MI:0493(in vivo)\|Affinity Capture-Western\|MI:0007(anti tag coimmunoprecipitation)\|MI:0081(peptide array)\|MI:0004(affinity chromatography technology)\|MI:0018(two hybrid)\|MI:0096(pull down) |
| TNK2 | CDC42 | 0.9 | MI:0492(in vitro)\|MI:0493(in vivo)\|Affinity Capture-Western\|MI:0077(nuclear magnetic resonance)\|MI:0004(affinity chromatography technology)\|MI:0018(two hybrid)\|MI:0096(pull down)\|MI:0114(x-ray crystallography)\|MI:0428(imaging technique)\|MI:0055(fluorescent resonance energy transfer) |
| TNK2 | CLTC | 0.9 | MI:0492(in vitro)\|MI:0493(in vivo)\|Reconstituted Complex\|MI:0007(anti tag coimmunoprecipitation)\|MI:0096(pull down)\|MI:0004(affinity chromatography technology) |
| TNK2 | FYN | 0.9 | MI:0492(in vitro)\|MI:0493(in vivo)\|Affinity Capture-Western\|Reconstituted Complex\|MI:0004(affinity chromatography technology)\|MI:0096(pull down) |
| TNK2 | NCK1 | 0.89 | MI:0493(in vivo)\|MI:0081(peptide array)\|MI:0096(pull down)\|MI:0004(affinity chromatography technology)\|MI:0007(anti tag coimmunoprecipitation) |
| TNK2 | EGFR | 0.85 | MI:0493(in vivo)\|MI:0006(anti bait coimmunoprecipitation)\|MI:0004(affinity chromatography technology)\|MI:0428(imaging technique) |
| TNK2 | AR | 0.84 | MI:0004(affinity chromatography technology)\|MI:0096(pull down)\|MI:0007(anti tag coimmunoprecipitation) |
| TNK2 | HSP90AB1 | 0.83 | MI:0004(affinity chromatography technology)\|MI:0007(anti tag coimmunoprecipitation) |
| TNK2 | HSP90AA1 | 0.83 | MI:0004(affinity chromatography technology)\|MI:0007(anti tag coimmunoprecipitation) |
| TNK2 | CDC37 | 0.83 | MI:0004(affinity chromatography technology)\|MI:0007(anti tag coimmunoprecipitation) |
| TNK2 | HSP90AB3P | 0.83 | MI:0004(affinity chromatography technology)\|MI:0007(anti tag coimmunoprecipitation) |
| TNK2 | SIAH1 | 0.82 | MI:0018(two hybrid)\|MI:0004(affinity chromatography technology) |
| TNK2 | SLC25A5 | 0.82 | MI:0004(affinity chromatography technology)\|MI:0007(anti tag coimmunoprecipitation) |
| TNK2 | ISG15 | 0.82 | MI:0004(affinity chromatography technology)\|MI:0007(anti tag coimmunoprecipitation) |
| TNK2 | NEDD4 | 0.8 | MI:0415(enzymatic study)\|MI:0004(affinity chromatography technology)\|MI:0096(pull down) |
| TNK2 | NEDD4L | 0.8 | MI:0004(affinity chromatography technology)\|MI:0096(pull down)\|MI:0428(imaging technique) |
| TNK2 | AKT1 | 0.78 | MI:0004(affinity chromatography technology)\|MI:0096(pull down) |
| TNK2 | BCAR1 | 0.75 | MI:0096(pull down)\|MI:0006(anti bait coimmunoprecipitation)\|MI:0004(affinity chromatography technology) |
| TNK2 | PDGFRB | 0.75 | MI:0004(affinity chromatography technology)\|MI:0415(enzymatic study) |
| TNK2 | MCF2 | 0.74 | MI:0492(in vitro)\|MI:0493(in vivo)\|Affinity Capture-Western\|MI:0004(affinity chromatography technology) |
| TNK2 | CCT2 | 0.72 | MI:0007(anti tag coimmunoprecipitation)\|MI:0004(affinity chromatography technology) |
| TNK2 | HSPD1 | 0.72 | MI:0007(anti tag coimmunoprecipitation)\|MI:0004(affinity chromatography technology) |
| TNK2 | CCT8 | 0.72 | MI:0007(anti tag coimmunoprecipitation)\|MI:0004(affinity chromatography technology) |
| TNK2 | CCT4 | 0.72 | MI:0007(anti tag coimmunoprecipitation)\|MI:0004(affinity chromatography technology) |
| TNK2 | CCT6A | 0.72 | MI:0007(anti tag coimmunoprecipitation)\|MI:0004(affinity chromatography technology) |
| TNK2 | TCP1 | 0.72 | MI:0007(anti tag coimmunoprecipitation)\|MI:0004(affinity chromatography technology) |
| TNK2 | MRPL55 | 0.72 | MI:0007(anti tag coimmunoprecipitation)\|MI:0004(affinity chromatography technology) |
| TNK2 | CCT5 | 0.72 | MI:0007(anti tag coimmunoprecipitation)\|MI:0004(affinity chromatography technology) |
| TNK2 | TUFM | 0.72 | MI:0007(anti tag coimmunoprecipitation)\|MI:0004(affinity chromatography technology) |
| TNK2 | CCT3 | 0.72 | MI:0007(anti tag coimmunoprecipitation)\|MI:0004(affinity chromatography technology) |
| TNS3 | PTK2 | 0.85 | MI:0047(far western blotting)\|MI:0416(fluorescence microscopy)\|MI:0096(pull down)\|MI:0004(affinity chromatography technology) |
| TNS4 | MET | 0.75 | MI:0053(fluorescence polarization spectroscopy)\|MI:0096(pull down) |
| TOP1 | PRKDC | 0.85 | MI:0006(anti bait coimmunoprecipitation)\|MI:0096(pull down)\|MI:0004(affinity chromatography technology)\|MI:0415(enzymatic study) |
| TP53 | MET | 0.75 | MI:0055(fluorescent resonance energy transfer) |
| TPCN2 | CERK | 0.82 | MI:0004(affinity chromatography technology)\|MI:0007(anti tag coimmunoprecipitation) |
| TPTE | PRKDC | 0.75 | MI:0004(affinity chromatography technology)\|MI:0096(pull down)\|bioid |
| TRADD | PRKDC | 0.72 | MI:0676(tandem affinity purification)\|MI:0007(anti tag coimmunoprecipitation) |
| TRAF3IP3 | STK24 | 0.78 | Affinity Capture-MS\|Affinity Capture-Western\|MI:0007(anti tag coimmunoprecipitation)\|MI:0004(affinity chromatography technology) |
| TRAF6 | PRKCZ | 0.83 | MI:0493(in vivo)\|Affinity Capture-Western\|MI:0004(affinity chromatography technology) |
| TRAK1 | HGS | 0.7 | MI:0004(affinity chromatography technology)\|MI:0096(pull down)\|MI:0428(imaging technique) |
| TRIM41 | PRKCZ | 0.83 | MI:0004(affinity chromatography technology)\|MI:0007(anti tag coimmunoprecipitation) |
| TRIP6 | PTK2 | 0.84 | MI:0493(in vivo)\|Affinity Capture-Western\|MI:0004(affinity chromatography technology) |
| TRRAP | CDK8 | 0.75 | MI:0006(anti bait coimmunoprecipitation)\|MI:0226(ion exchange chromatography)\|MI:0401(biochemical) |
| TSC2 | PTK2 | 0.78 | Affinity Capture-Western\|Reconstituted Complex\|MI:0004(affinity chromatography technology)\|MI:0096(pull down) |
| TSSC4 | PRPF6 | 0.83 | MI:0004(affinity chromatography technology)\|MI:0007(anti tag coimmunoprecipitation) |
| TTC4 | HGS | 0.72 | MI:0004(affinity chromatography technology)\|MI:0401(biochemical) |
| TTI1 | PRKDC | 0.85 | MI:0007(anti tag coimmunoprecipitation)\|MI:0096(pull down)\|MI:0004(affinity chromatography technology) |
| TTI2 | PRKDC | 0.72 | MI:0676(tandem affinity purification)\|MI:0004(affinity chromatography technology) |
| TXK | MET | 0.75 | MI:0053(fluorescence polarization spectroscopy)\|MI:0096(pull down) |
| TXNL4A | PRPF6 | 0.94 | MI:0004(affinity chromatography technology)\|MI:0018(two hybrid)\|MI:0007(anti tag coimmunoprecipitation) |
| TXNL4B | PRPF6 | 0.79 | MI:0492(in vitro)\|MI:0493(in vivo)\|MI:0018(two hybrid)\|Reconstituted Complex\|Affinity Capture-Western\|MI:0004(affinity chromatography technology)\|MI:0096(pull down) |
| UBQLN2 | HGS | 0.82 | MI:0018(two hybrid)\|MI:0004(affinity chromatography technology) |
| UBR5 | PRKDC | 0.73 | MI:0004(affinity chromatography technology) |
| USF1 | PRKDC | 0.78 | MI:0006(anti bait coimmunoprecipitation)\|MI:0007(anti tag coimmunoprecipitation)\|MI:0004(affinity chromatography technology)\|MI:0424(protein kinase assay) |
| USP39 | PRPF6 | 0.76 | Affinity Capture-MS\|MI:0007(anti tag coimmunoprecipitation)\|MI:0004(affinity chromatography technology) |
| VAPB | SPEG | 0.73 | MI:0004(affinity chromatography technology) |
| VAV3 | MET | 0.75 | MI:0053(fluorescence polarization spectroscopy)\|MI:0096(pull down) |
| VCAM1 | PRKDC | 0.83 | MI:0030(cross-linking study)\|MI:0004(affinity chromatography technology) |
| VCL | STK24 | 0.72 | MI:0004(affinity chromatography technology)\|MI:0401(biochemical) |
| VHL | PRKCZ | 0.85 | MI:0007(anti tag coimmunoprecipitation)\|MI:0022(colocalization by immunostaining)\|MI:0428(imaging technique)\|MI:0004(affinity chromatography technology) |
| VPS16 | PTK2 | 0.72 | MI:0004(affinity chromatography technology)\|MI:0007(anti tag coimmunoprecipitation) |
| VPS37A | HGS | 0.77 | MI:0492(in vitro)\|MI:0493(in vivo)\|MI:0018(two hybrid) |
| VPS37C | HGS | 0.75 | MI:0492(in vitro)\|MI:0018(two hybrid) |
| VWCE | CERK | 0.82 | MI:0004(affinity chromatography technology)\|MI:0007(anti tag coimmunoprecipitation) |
| WASHC1 | HGS | 0.75 | MI:0428(imaging technique)\|MI:0018(two hybrid) |
| WDR76 | PRKDC | 0.82 | MI:0004(affinity chromatography technology)\|MI:0401(biochemical) |
| WDR77 | FAM47E | 0.72 | MI:0007(anti tag coimmunoprecipitation)\|MI:0004(affinity chromatography technology) |
| WRN | PRKDC | 0.88 | MI:0492(in vitro)\|MI:0493(in vivo)\|Protein-peptide\|Affinity Capture-Western\|Biochemical Activity\|MI:0415(enzymatic study)\|MI:0004(affinity chromatography technology) |
| WWC1 | PRKCZ | 0.89 | MI:0415(enzymatic study)\|MI:0018(two hybrid)\|MI:0096(pull down)\|MI:0428(imaging technique)\|MI:0004(affinity chromatography technology)\|MI:0007(anti tag coimmunoprecipitation) |
| WWOX | TNK2 | 0.8 | MI:0493(in vivo)\|MI:0096(pull down)\|MI:0004(affinity chromatography technology) |
| XRCC4 | PRKDC | 0.85 | MI:0492(in vitro)\|MI:0004(affinity chromatography technology)\|MI:0415(enzymatic study)\|MI:0401(biochemical) |
| XRCC5 | PRKDC | 0.9 | MI:0424(protein kinase assay)\|MI:0006(anti bait coimmunoprecipitation)\|MI:0004(affinity chromatography technology)\|MI:0096(pull down)\|MI:0428(imaging technique)\|MI:0415(enzymatic study)\|MI:0114(x-ray crystallography)\|MI:0007(anti tag coimmunoprecipitation)\|MI:0401(biochemical) |
| XRCC6 | PRKDC | 0.9 | MI:0424(protein kinase assay)\|MI:0006(anti bait coimmunoprecipitation)\|MI:0004(affinity chromatography technology)\|MI:0415(enzymatic study)\|MI:0007(anti tag coimmunoprecipitation)\|MI:0401(biochemical) |
| YWHAB | PFKFB4 | 0.82 | MI:0007(anti tag coimmunoprecipitation)\|MI:0004(affinity chromatography technology) |
| YWHAB | HGS | 0.72 | MI:0007(anti tag coimmunoprecipitation)\|MI:0004(affinity chromatography technology) |
| YWHAG | PFKFB4 | 0.82 | MI:0007(anti tag coimmunoprecipitation)\|MI:0004(affinity chromatography technology) |
| YWHAH | PFKFB4 | 0.82 | MI:0007(anti tag coimmunoprecipitation)\|MI:0004(affinity chromatography technology) |
| YWHAQ | PFKFB4 | 0.82 | MI:0007(anti tag coimmunoprecipitation)\|MI:0004(affinity chromatography technology) |
| YWHAQ | PRKDC | 0.78 | MI:0004(affinity chromatography technology)\|MI:0096(pull down) |
| YWHAQ | SPEG | 0.73 | MI:0004(affinity chromatography technology) |
| YWHAZ | PRKCZ | 0.89 | Reconstituted Complex\|Biochemical Activity\|MI:0415(enzymatic study)\|MI:0096(pull down)\|MI:0004(affinity chromatography technology) |
| YWHAZ | SIK3 | 0.87 | MI:0492(in vitro)\|MI:0493(in vivo)\|MI:0676(tandem affinity purification)\|MI:0096(pull down)\|MI:0004(affinity chromatography technology) |
| YWHAZ | PRKDC | 0.84 | MI:0059(gst pull down)\|MI:0676(tandem affinity purification)\|MI:0096(pull down) |
| YWHAZ | PFKFB4 | 0.82 | MI:0007(anti tag coimmunoprecipitation)\|MI:0004(affinity chromatography technology) |
| YY1 | PRKDC | 0.71 | MI:0029(cosedimentation through density gradient)\|MI:0096(pull down)\|MI:0401(biochemical) |
| ZNHIT2 | PRPF6 | 0.72 | MI:0004(affinity chromatography technology)\|bioid |

**Table 2: Hub genes identified within the network based on closeness, degree, and betweenness.**

| Degree | | | Betweenness | | | Closeness | | |
| --- | --- | --- | --- | --- | --- | --- | --- | --- |
| Rank | Name | Score | Rank | Name | Score | Rank | Name | Score |
| 1 | PRKDC | 178 | 1 | HGS | 310550.1933 | 1 | HGS | 422.65 |
| 2 | HGS | 155 | 2 | PRKDC | 292997.7275 | 2 | PRKDC | 416.9833333 |
| 3 | PRKCZ | 130 | 3 | PRKCZ | 200262.5646 | 3 | EGFR | 389.1666667 |
| 4 | MET | 115 | 4 | MET | 168485.7995 | 4 | MET | 386.8166667 |
| 5 | PTK2 | 96 | 5 | CDK8 | 134628.0174 | 5 | PRKCZ | 375.4166667 |
| 6 | CDK8 | 83 | 6 | PRPF6 | 122275.9568 | 6 | HSP90AA1 | 349.8166667 |
| 7 | PRPF6 | 77 | 7 | PTK2 | 117572.5329 | 7 | HSPA8 | 349.75 |
| 8 | STK24 | 72 | 8 | STK24 | 107076.0468 | 8 | PTK2 | 339.0833333 |
| 9 | TNK2 | 44 | 9 | EGFR | 73085.35932 | 9 | PRPF6 | 334.6333333 |
| 10 | CERK | 41 | 10 | CERK | 61211.16466 | 10 | CDK8 | 329.15 |

**Table 3: Mutational analysis of the hub genes using the UniProt Database.**

| **Gene** | **Variant ID(s)** | **Position(s)** | **Change** | **Description** | **Disease association** |
| --- | --- | --- | --- | --- | --- |
| PRKDC | 876660b7-347a-407b-93e5-4fe9fdd890d3 | 40 | [Q>A](https://www.ebi.ac.uk/ProtVar/query?search=P78527%20Q40A) | Variant assessed as Somatic; HIGH impact. (NCI-TCGA) | Adenomas and Adenocarcinomas |
|  | 0c2f4c89-0504-455b-ab29-baee60621fbe | 77 | [E>*](https://www.ebi.ac.uk/ProtVar/query?search=P78527%20E77*) | Variant assessed as Somatic; HIGH impact. (NCI-TCGA) | Adenomas and Adenocarcinomas |
|  | cb5852aa-1f4e-439e-b815-981d2dd68384 |  |  |  |  |
|  | 2894830a-50f7-49c3-bacf-d32474637417 | 84 | [E>*](https://www.ebi.ac.uk/ProtVar/query?search=P78527%20E84*) | Variant assessed as Somatic; HIGH impact. (NCI-TCGA) | Adenomas and Adenocarcinomas |
|  | 79d2bad3-5c00-42c3-91b9-e30c05ac6c1d |  |  |  |  |
|  | COSV50621725 |  |  |  |  |
|  | COSV100031496 | 106 | [E>*](https://www.ebi.ac.uk/ProtVar/query?search=P78527%20E106*) | Variant assessed as Somatic; HIGH impact. (NCI-TCGA) | Cystic, Mucinous and Serous Neoplasms |
|  | c96ca0b4-6190-41e9-bdd8-2f17ca9d1474 |  |  |  |  |
|  | 195ca5a2-9ca6-4ed5-bb1c-a363a6e93845 | 159 | [E>*](https://www.ebi.ac.uk/ProtVar/query?search=P78527%20E159*) | Variant assessed as Somatic; HIGH impact. (NCI-TCGA) | Adenomas and Adenocarcinomas |
|  | COSV58043763 |  |  |  |  |
|  | 0702253c-6de7-4986-9df4-faba66f9af0d | 166 | [I>Y](https://www.ebi.ac.uk/ProtVar/query?search=P78527%20I166Y) | Variant assessed as Somatic; HIGH impact. (NCI-TCGA) | Adenomas and Adenocarcinomas; Cystic, Mucinous and Serous Neoplasms; Squamous Cell Neoplasms; Thymic Epithelial Neoplasms |
|  | 0b10b9f6-c4da-4b4d-b87c-171c3c12193d |  |  |  |  |
|  | 18daee2a-d3e3-41a5-82bf-bfc83945a9e2 |  |  |  |  |
|  | 21030d58-debe-42aa-b940-e26994487097 |  |  |  |  |
|  | 2dc55512-d57b-4062-9068-b47848e5fd01 |  |  |  |  |
|  | 3d2748d2-d444-466c-b1f2-8b7b0d612e0f |  |  |  |  |
|  | 577f5df7-8d80-4f63-8f48-2369b19b6342 |  |  |  |  |
|  | 5c5f2f4e-d25e-491c-8805-c902d526673e |  |  |  |  |
|  | 5e1dbe36-c1dc-4d42-8618-80b4f480bf03 |  |  |  |  |
|  | 843b4e74-0574-4bfc-8d30-49036631d37d |  |  |  |  |
|  | 92fd8c1c-7e24-404b-9c0b-51b080145f2e |  |  |  |  |
|  | a34c7bfa-9a11-4974-812f-dc682482d39b |  |  |  |  |
|  | bf81db23-a848-4b47-a3bf-16a3d77fc720 |  |  |  |  |
|  | ece05d95-2c23-4d65-81ba-9dd708437b7b |  |  |  |  |
|  | ef1ae5ff-c60c-48ea-a0dc-954d92699899 |  |  |  |  |
|  | rs537061158 |  |  |  |  |
|  | 30668a78-56f1-46bc-b24b-b77619091078 | 229 | [S>*](https://www.ebi.ac.uk/ProtVar/query?search=P78527%20S229*) | Variant assessed as Somatic; HIGH impact. (NCI-TCGA) | Transitional Cell Papillomas and Carcinomas; Squamous Cell Neoplasms |
|  | COSV100038465 |  |  |  |  |
|  | 2aa0e531-67a9-4aee-8098-4d8ebbd2a1ec |  |  |  |  |
|  | 41002e4d-708d-4dd3-9f2d-315199068b3a | 245 | [S>*](https://www.ebi.ac.uk/ProtVar/query?search=P78527%20S245*) | Variant assessed as Somatic; HIGH impact. (NCI-TCGA) | Adenomas and Adenocarcinomas |
|  | 63362632-a822-42ea-bba3-c24d044bac39 |  |  |  |  |
|  | f03292ba-467a-4cad-a0cc-6d342ee0af38 | 311 | [A>S](https://www.ebi.ac.uk/ProtVar/query?search=P78527%20A311S) | Variant assessed as Somatic; HIGH impact. (NCI-TCGA) | Adenomas and Adenocarcinomas |
|  | f7ad6efa-36c4-43f7-bb2e-a5ef297c8e8a | 336 | [N>I](https://www.ebi.ac.uk/ProtVar/query?search=P78527%20N336I) | Variant assessed as Somatic; HIGH impact. (NCI-TCGA) | Adenomas and Adenocarcinomas |
|  | 69974ebc-ce66-40f7-a08d-3be34d71b408 | 336 | [N>K](https://www.ebi.ac.uk/ProtVar/query?search=P78527%20N336K) | Variant assessed as Somatic; HIGH impact. (NCI-TCGA) | Cystic, Mucinous and Serous Neoplasms |
|  | 3a57f209-4e45-4ded-ae72-c86b5224e082 | 340 | [Y>*](https://www.ebi.ac.uk/ProtVar/query?search=P78527%20Y340*) | Variant assessed as Somatic; HIGH impact. (NCI-TCGA) | Adenomas and Adenocarcinomas |
|  | ac54e017-3242-4fe7-bade-301b0bdf9fbc |  |  |  |  |
|  | 18012079-216a-41fc-87f8-dff8ea43c840 | 344 | [Q>*](https://www.ebi.ac.uk/ProtVar/query?search=P78527%20Q344*) | Variant assessed as Somatic; HIGH impact. (NCI-TCGA) | Adenomas and Adenocarcinomas |
|  | COSV58052250 |  |  |  |  |
|  | 3b5d6636-da6f-4510-9ca9-3043096a6f84 | 387 | [E>*](https://www.ebi.ac.uk/ProtVar/query?search=P78527%20E387*) | Variant assessed as Somatic; HIGH impact. (NCI-TCGA) | Gliomas |
|  | 85c238cb-6a1f-41d1-bd86-aff9c72e9fd7 | 390 | [Q>*](https://www.ebi.ac.uk/ProtVar/query?search=P78527%20Q390*) | Variant assessed as Somatic; HIGH impact. (NCI-TCGA) | Adenomas and Adenocarcinomas |
|  | COSV100040681 |  |  |  |  |
|  | 26b51394-bd5c-40e7-b1b2-28cc46d57284 | 548 | [E>*](https://www.ebi.ac.uk/ProtVar/query?search=P78527%20E548*) | Variant assessed as Somatic; HIGH impact. (NCI-TCGA) | Adenomas and Adenocarcinomas |
|  | COSV58063306 |  |  |  |  |
|  | 354c131d-9f78-45a9-87fc-5055b2f87907 | 580 | [D>I](https://www.ebi.ac.uk/ProtVar/query?search=P78527%20D580I) | Variant assessed as Somatic; HIGH impact. (NCI-TCGA) | Adenomas and Adenocarcinomas |
|  | 61845078-b637-4257-a4a0-4aa782bba1f2 | 640 | [E>*](https://www.ebi.ac.uk/ProtVar/query?search=P78527%20E640*) | Variant assessed as Somatic; HIGH impact. (NCI-TCGA) | Adenomas and Adenocarcinomas |
|  | COSV58046527 |  |  |  |  |
|  | 859de131-2bfc-4acf-9a8a-eda4cbb54764 | 643 | [E>*](https://www.ebi.ac.uk/ProtVar/query?search=P78527%20E643*) | Variant assessed as Somatic; HIGH impact. (NCI-TCGA) | Squamous Cell Neoplasms |
|  | COSV100039427 |  |  |  |  |
|  | 52e1e5b7-73ba-4e61-bfcc-14529bd8de33 | 648 | [S>*](https://www.ebi.ac.uk/ProtVar/query?search=P78527%20S648*) | Variant assessed as Somatic; HIGH impact. (NCI-TCGA) | Squamous Cell Neoplasms |
|  | 36f43958-6794-4a65-9f95-f1a4bea48533 | 699 | [E>*](https://www.ebi.ac.uk/ProtVar/query?search=P78527%20E699*) | Variant assessed as Somatic; HIGH impact. (NCI-TCGA) | Adenomas and Adenocarcinomas |
|  | 13186974-be25-4bab-8b3f-d70eca574a09 | 713 | [E>G](https://www.ebi.ac.uk/ProtVar/query?search=P78527%20E713G) | Variant assessed as Somatic; HIGH impact. (NCI-TCGA) | Squamous Cell Neoplasms |
|  | COSV58051055 | 742 | [E>*](https://www.ebi.ac.uk/ProtVar/query?search=P78527%20E742*) | Variant assessed as Somatic; HIGH impact. (NCI-TCGA) | Adenomas and Adenocarcinomas |
|  | b77415e6-52ca-47ca-aa01-5ddb92f94e0f |  |  |  |  |
|  | COSV58055162 | 803 | [S>*](https://www.ebi.ac.uk/ProtVar/query?search=P78527%20S803*) | Variant assessed as Somatic; HIGH impact. (NCI-TCGA) | Ductal and Lobular Neoplasms |
|  | a32a11fa-3d87-43af-8086-4015a0acd8b8 |  |  |  |  |
|  | 972f35c2-a12e-4f0f-8603-d62c736d76b5 | 878 | [E>*](https://www.ebi.ac.uk/ProtVar/query?search=P78527%20E878*) | Variant assessed as Somatic; HIGH impact. (NCI-TCGA) | Adenomas and Adenocarcinomas |
|  | COSV100041008 |  |  |  |  |
|  | cc865108-ace4-4af8-87bc-ad7b3f7b0d2a |  |  |  |  |
|  | f3eb5735-f240-4024-ba48-9036aa5049b3 |  |  |  |  |
|  | rs1319344777 |  |  |  |  |
|  | 694fe2ed-837b-49e8-b59d-5d54af5d7a00 | 883 | [Y>L](https://www.ebi.ac.uk/ProtVar/query?search=P78527%20Y883L) | Variant assessed as Somatic; HIGH impact. (NCI-TCGA) | Adenomas and Adenocarcinomas |
|  | 540c73f3-03f2-4ffb-8a45-28e0498ad156 | 913 | [R>*](https://www.ebi.ac.uk/ProtVar/query?search=P78527%20R913*) | Variant assessed as Somatic; HIGH impact. (NCI-TCGA) | Adenomas and Adenocarcinomas |
|  | 7cd5cedb-0793-4d3a-97eb-f289e233e2e0 |  |  |  |  |
|  | COSV58059167 |  |  |  |  |
|  | 49733dbf-4450-4790-a8fc-5245a6650379 | 932 | [E>*](https://www.ebi.ac.uk/ProtVar/query?search=P78527%20E932*) | Variant assessed as Somatic; HIGH impact. (NCI-TCGA) | Adenomas and Adenocarcinomas |
|  | e4feadd3-9aec-406f-88bc-34ecd3f39470 | 952 | [G>*](https://www.ebi.ac.uk/ProtVar/query?search=P78527%20G952*) | Variant assessed as Somatic; HIGH impact. (NCI-TCGA) | Cystic, Mucinous and Serous Neoplasms |
|  | feaac4c4-14f5-4e77-8970-daddc41ef297 | 954 | [G>*](https://www.ebi.ac.uk/ProtVar/query?search=P78527%20G954*) | Variant assessed as Somatic; HIGH impact. (NCI-TCGA) | Adenomas and Adenocarcinomas |
|  | 89ce7f62-233d-4f7d-bff0-303297594b74 | 973 | [A>G](https://www.ebi.ac.uk/ProtVar/query?search=P78527%20A973G) | Variant assessed as Somatic; HIGH impact. (NCI-TCGA) | Plasma Cell Tumors |
|  | 1a44288a-a3b0-42d9-ae77-09ba460000d5 | 994 | [W>*](https://www.ebi.ac.uk/ProtVar/query?search=P78527%20W994*) | Variant assessed as Somatic; HIGH impact. (NCI-TCGA) | Gliomas |
|  | COSV100040777 | 1049 | [Q>*](https://www.ebi.ac.uk/ProtVar/query?search=P78527%20Q1049*) | Variant assessed as Somatic; HIGH impact. (NCI-TCGA) | Adenomas and Adenocarcinomas |
|  | ad5b8a88-07c2-4e35-a1e4-6e5a50c96f51 |  |  |  |  |
|  | e4fc705d-7208-42fd-ad86-f8c0cc5f25e8 | 1060 | [F>S](https://www.ebi.ac.uk/ProtVar/query?search=P78527%20F1060S) | Variant assessed as Somatic; HIGH impact. (NCI-TCGA) | Adenomas and Adenocarcinomas |
|  | 83a9cce5-05c5-4b3f-afa4-d0be9e2f274b | 1074 | [K>*](https://www.ebi.ac.uk/ProtVar/query?search=P78527%20K1074*) | Variant assessed as Somatic; HIGH impact. (NCI-TCGA) | Adenomas and Adenocarcinomas |
|  | COSV58061693 |  |  |  |  |
|  | 99e27a1e-bf8c-43e3-ba91-53b90e92c9a2 | 1088 | [E>*](https://www.ebi.ac.uk/ProtVar/query?search=P78527%20E1088*) | Variant assessed as Somatic; HIGH impact. (NCI-TCGA) | Neoplasms, NOS |
|  | COSV58054825 |  |  |  |  |
|  | rs1432931768 |  |  |  |  |
|  | COSV100041679 | 1125 | [Q>*](https://www.ebi.ac.uk/ProtVar/query?search=P78527%20Q1125*) | Variant assessed as Somatic; HIGH impact. (NCI-TCGA) | Adenomas and Adenocarcinomas |
|  | ba5968c3-892b-48eb-90c7-6d82bf315984 |  |  |  |  |
|  | 59b3d371-c9ff-465b-bf35-9539a3fbfd9e | 1162 | [S>*](https://www.ebi.ac.uk/ProtVar/query?search=P78527%20S1162*) | Variant assessed as Somatic; HIGH impact. (NCI-TCGA) | Transitional Cell Papillomas and Carcinomas |
|  | COSV100038138 |  |  |  |  |
|  | 909eccb9-b23b-411a-b9a5-ab5867bde009 | 1164 | [C>F](https://www.ebi.ac.uk/ProtVar/query?search=P78527%20C1164F) | Variant assessed as Somatic; HIGH impact. (NCI-TCGA) | Gliomas |
|  | 56542cb6-98d6-4d54-8f73-3dfb6dda50eb | 1277 | [G>*](https://www.ebi.ac.uk/ProtVar/query?search=P78527%20G1277*) | Variant assessed as Somatic; HIGH impact. (NCI-TCGA) | Squamous Cell Neoplasms |
|  | COSV105900671 |  |  |  |  |
|  | f0ef6914-26c0-47bc-87fa-8e2614a2dbef | 1339 | [V>S](https://www.ebi.ac.uk/ProtVar/query?search=P78527%20V1339S) | Variant assessed as Somatic; HIGH impact. (NCI-TCGA) | Squamous Cell Neoplasms |
|  | 580decc4-f025-42b6-8b3e-6d58dc40f1c3 | 1365 | [N>*](https://www.ebi.ac.uk/ProtVar/query?search=P78527%20N1365*) | Variant assessed as Somatic; HIGH impact. (NCI-TCGA) | Nerve Sheath Tumors |
|  | eab462c2-3aa9-477c-9e85-a12963d4c2a0 |  |  |  |  |
|  | 6ace8aae-fea5-477e-9e9b-74979e647cf8 | 1423 | [I>*](https://www.ebi.ac.uk/ProtVar/query?search=P78527%20I1423*) | Variant assessed as Somatic; HIGH impact. (NCI-TCGA) | Cystic, Mucinous and Serous Neoplasms |
|  | fc97ede5-89d6-4778-90aa-ddc00530a54d | 1482 | [E>*](https://www.ebi.ac.uk/ProtVar/query?search=P78527%20E1482*) | Variant assessed as Somatic; HIGH impact. (NCI-TCGA) | Adenomas and Adenocarcinomas |
|  | 5f0e2f26-1ba2-4b11-834c-be790ded0cd0 | 1544 | [G>A](https://www.ebi.ac.uk/ProtVar/query?search=P78527%20G1544A) | Variant assessed as Somatic; HIGH impact. (NCI-TCGA) | Squamous Cell Neoplasms |
|  | COSV58052371 |  |  |  |  |
|  | 32f5e290-97c3-4945-9eb5-2448aaebd9c0 | 1564 | [S>*](https://www.ebi.ac.uk/ProtVar/query?search=P78527%20S1564*) | Variant assessed as Somatic; HIGH impact. (NCI-TCGA) | Adenomas and Adenocarcinomas |
|  | COSV100043171 |  |  |  |  |
|  | 110a19b8-50d4-4a30-b64b-1cd6b76debeb | 1592 | [M>N](https://www.ebi.ac.uk/ProtVar/query?search=P78527%20M1592N) | Variant assessed as Somatic; HIGH impact. (NCI-TCGA) | Adenomas and Adenocarcinomas |
|  | cac32d98-82bd-4dc5-a0cb-1ff597d14fcd | 1608 | [R>*](https://www.ebi.ac.uk/ProtVar/query?search=P78527%20R1608*) | Variant assessed as Somatic; HIGH impact. (NCI-TCGA) | Adenomas and Adenocarcinomas |
|  | bfc4d5d4-1150-4ee7-8dd3-f187e2a042bd | 1653 | [L>F](https://www.ebi.ac.uk/ProtVar/query?search=P78527%20L1653F) | Variant assessed as Somatic; HIGH impact. (NCI-TCGA) | Adenomas and Adenocarcinomas |
|  | 8de23acc-2a2d-4de2-9abb-76196080a75d | 1657 | [S>*](https://www.ebi.ac.uk/ProtVar/query?search=P78527%20S1657*) | Variant assessed as Somatic; HIGH impact. (NCI-TCGA) | Cystic, Mucinous and Serous Neoplasms |
|  | 9d311e6f-0820-4290-b461-8491edbf3bb0 |  |  |  |  |
|  | COSV58065584 |  |  |  |  |
|  | 152d0c5f-2c1f-45ae-bdf4-4453e3472e9f | 1691 | [Q>*](https://www.ebi.ac.uk/ProtVar/query?search=P78527%20Q1691*) | Variant assessed as Somatic; HIGH impact. (NCI-TCGA) | Adenomas and Adenocarcinomas |
|  | 90996c3f-34f4-47c4-9d0e-c36dc2f1b01c |  |  |  |  |
|  | baf4c906-6b79-4220-86cf-23c416fd6c74 | 1704 | [G>D](https://www.ebi.ac.uk/ProtVar/query?search=P78527%20G1704D) | Variant assessed as Somatic; HIGH impact. (NCI-TCGA) | Adenomas and Adenocarcinomas |
|  | 327b735b-f8d1-4142-b8bc-2ea446df4f51 | 1760 | [E>*](https://www.ebi.ac.uk/ProtVar/query?search=P78527%20E1760*) | Variant assessed as Somatic; HIGH impact. (NCI-TCGA) | Ductal and Lobular Neoplasms |
|  | COSV100040578 |  |  |  |  |
|  | 26ed5122-5cef-45f3-83b9-5af1b68a879b | 1777 | [L>*](https://www.ebi.ac.uk/ProtVar/query?search=P78527%20L1777*) | Variant assessed as Somatic; HIGH impact. (NCI-TCGA) | Gliomas |
|  | COSV100042249 |  |  |  |  |
|  | 7adecc8d-f2d6-44f7-944a-2f0db11efa01 | 1779 | [Q>*](https://www.ebi.ac.uk/ProtVar/query?search=P78527%20Q1779*) | Variant assessed as Somatic; HIGH impact. (NCI-TCGA) | Squamous Cell Neoplasms |
|  | COSV100040195 |  |  |  |  |
|  | COSV58059841 |  |  |  |  |
|  | COSV100042979 | 1799 | [E>*](https://www.ebi.ac.uk/ProtVar/query?search=P78527%20E1799*) | Variant assessed as Somatic; HIGH impact. (NCI-TCGA) | Ductal and Lobular Neoplasms |
|  | ae76e7e9-ea0a-4917-b1dc-781bf16a4d17 |  |  |  |  |
|  | 4152e8ad-21fa-4d37-9ddf-1e7dc7cd0745 | 1925 | [E>*](https://www.ebi.ac.uk/ProtVar/query?search=P78527%20E1925*) | Variant assessed as Somatic; HIGH impact. (NCI-TCGA) | Adenomas and Adenocarcinomas |
|  | 9f0da6b3-341b-43d6-8220-c328eccb99ee |  |  |  |  |
|  | COSV100041911 |  |  |  |  |
|  | e955024b-d48c-4b1b-9202-f91d85e605be |  |  |  |  |
|  | 08c9df3a-295a-4fa7-965b-feda8d8b187a | 1935 | [E>*](https://www.ebi.ac.uk/ProtVar/query?search=P78527%20E1935*) | Variant assessed as Somatic; HIGH impact. (NCI-TCGA) | Ductal and Lobular Neoplasms |
|  | 0ddb96f5-835f-4653-be32-7272a248e00a | 2042 | [Q>*](https://www.ebi.ac.uk/ProtVar/query?search=P78527%20Q2042*) | Variant assessed as Somatic; HIGH impact. (NCI-TCGA) | Gliomas |
|  | COSV58047529 |  |  |  |  |
|  | COSV58067452 |  |  |  |  |
|  | a9e4beea-4851-43dd-bbba-58139b6bc034 |  |  |  |  |
|  | 67b65aeb-d2f0-45c3-b5bb-9fa556a956ed | 2046 | [S>*](https://www.ebi.ac.uk/ProtVar/query?search=P78527%20S2046*) | Variant assessed as Somatic; HIGH impact. (NCI-TCGA) | Nevi and Melanomas |
|  | 86ee8184-720c-4ee5-b96a-3e99ffef5fe8 |  |  |  |  |
|  | COSV58045316 |  |  |  |  |
|  | 0575be03-6c30-490b-b98c-57a2dbf5a5b6 | 2053 | [S>*](https://www.ebi.ac.uk/ProtVar/query?search=P78527%20S2053*) | Variant assessed as Somatic; HIGH impact. (NCI-TCGA) | Adenomas and Adenocarcinomas |
|  | COSV58042234 |  |  |  |  |
|  | COSV58051191 |  |  |  |  |
|  | ac54e017-3242-4fe7-bade-301b0bdf9fbc |  |  |  |  |
|  | 6c3240d2-fc45-4034-92ad-450df0cd5deb | 2183 | [H>L](https://www.ebi.ac.uk/ProtVar/query?search=P78527%20H2183L) | Variant assessed as Somatic; HIGH impact. (NCI-TCGA) | Adenomas and Adenocarcinomas |
|  | 14484730-71c5-410f-9c92-724628a32399 | 2195 | [S>*](https://www.ebi.ac.uk/ProtVar/query?search=P78527%20S2195*) | Variant assessed as Somatic; HIGH impact. (NCI-TCGA) | Acute Lymphoblastic Leukemia |
|  | 1320ce12-4bc7-4741-9f49-3ea1f70b26c8 | 2214 | [R>*](https://www.ebi.ac.uk/ProtVar/query?search=P78527%20R2214*) | Variant assessed as Somatic; HIGH impact. (NCI-TCGA) | Adenomas and Adenocarcinomas |
|  | COSV58042368 |  |  |  |  |
|  | dd05be4b-7741-4f74-82b0-9958cd57114c |  |  |  |  |
|  | rs1589745056 |  |  |  |  |
|  | 4f345879-668b-4d8d-82e0-6835fdbdbdc5 | 2221 | [K>N](https://www.ebi.ac.uk/ProtVar/query?search=P78527%20K2221N) | Variant assessed as Somatic; HIGH impact. (NCI-TCGA) | Cystic, Mucinous and Serous Neoplasms |
|  | 05b65962-0707-45e3-a47a-94df5fbba96f | 2244 | [C>*](https://www.ebi.ac.uk/ProtVar/query?search=P78527%20C2244*) | Variant assessed as Somatic; HIGH impact. (NCI-TCGA) | Cystic, Mucinous and Serous Neoplasms |
|  | 4fa96995-67f7-4f09-8fda-2cb6db742cd1 | 2328 | [R>*](https://www.ebi.ac.uk/ProtVar/query?search=P78527%20R2328*) | Variant assessed as Somatic; HIGH impact. (NCI-TCGA) | Adenomas and Adenocarcinomas |
|  | c32ee117-12f7-49af-af88-4a98e9a4d5ea | 2397 | [C>W](https://www.ebi.ac.uk/ProtVar/query?search=P78527%20C2397W) | Variant assessed as Somatic; HIGH impact. (NCI-TCGA) | Adenomas and Adenocarcinomas |
|  | 42d54135-7cf5-4cc0-9bea-85461f77757b | 2407 | [G>*](https://www.ebi.ac.uk/ProtVar/query?search=P78527%20G2407*) | Variant assessed as Somatic; HIGH impact. (NCI-TCGA) | Squamous Cell Neoplasms |
|  | COSV100039161 |  |  |  |  |
|  | 1e02444f-2be2-481e-ae3f-550c24e9c532 | 2496 | [Q>*](https://www.ebi.ac.uk/ProtVar/query?search=P78527%20Q2496*) | Variant assessed as Somatic; HIGH impact. (NCI-TCGA) | Adenomas and Adenocarcinomas |
|  | rs1356241356 |  |  |  |  |
|  | b2f8736d-6dc5-46bf-bdee-67ce8f85fb79 | 2510 | [L>F](https://www.ebi.ac.uk/ProtVar/query?search=P78527%20L2510F) | Variant assessed as Somatic; HIGH impact. (NCI-TCGA) | Adenomas and Adenocarcinomas |
|  | 4af582cb-aa0a-4593-8694-155264d16a7e | 2555 | [L>*](https://www.ebi.ac.uk/ProtVar/query?search=P78527%20L2555*) | Variant assessed as Somatic; HIGH impact. (NCI-TCGA) | Adenomas and Adenocarcinomas |
|  | rs34870758 |  |  |  |  |
|  | COSV58045742 | 2598 | [R>*](https://www.ebi.ac.uk/ProtVar/query?search=P78527%20R2598*) | Variant assessed as Somatic; HIGH impact. (NCI-TCGA) | Adenomas and Adenocarcinomas |
|  | fd17c199-224e-4d3e-b16a-d04873c8b645 |  |  |  |  |
|  | 0ee5c272-1717-45a5-a80d-add0f3dbd271 | 2617 | [Q>*](https://www.ebi.ac.uk/ProtVar/query?search=P78527%20Q2617*) | Variant assessed as Somatic; HIGH impact. (NCI-TCGA) | Nevi and Melanomas |
|  | 54e326c0-752f-45f4-a455-9e68a8787599 |  |  |  |  |
|  | COSV105900484 |  |  |  |  |
|  |  |  |  |  |  |
|  | 784b5686-a687-4bd6-a795-6fedf777a123 | 2727 | [R>*](https://www.ebi.ac.uk/ProtVar/query?search=P78527%20R2727*) | Variant assessed as Somatic; HIGH impact. (NCI-TCGA) | Adenomas and Adenocarcinomas |
|  | COSV58044373 |  |  |  |  |
|  | rs1212909068 |  |  |  |  |
|  | COSV58049590 | 2753 | [R>*](https://www.ebi.ac.uk/ProtVar/query?search=P78527%20R2753*) | Variant assessed as Somatic; HIGH impact. (NCI-TCGA) | Adenomas and Adenocarcinomas |
|  | b882d1ba-b482-413a-85d5-a621d8a858e1 |  |  |  |  |
|  | 938540d7-6af7-42e9-811b-713a216f81af | 2763 | [M>*](https://www.ebi.ac.uk/ProtVar/query?search=P78527%20M2763*) | Variant assessed as Somatic; HIGH impact. (NCI-TCGA) | Adenomas and Adenocarcinomas |
|  | ab095981-8398-497e-ad2b-c824f4d1998b | 2830 | [N>T](https://www.ebi.ac.uk/ProtVar/query?search=P78527%20N2830T) | Variant assessed as Somatic; HIGH impact. (NCI-TCGA) | Adenomas and Adenocarcinomas |
|  | COSV58058534 | 2864 | [Q>*](https://www.ebi.ac.uk/ProtVar/query?search=P78527%20Q2864*) | Variant assessed as Somatic; HIGH impact. (NCI-TCGA) | Plasma Cell Tumors; Soft Tissue Tumors and Sarcomas, NOS |
|  | e0b11c00-2254-446d-954a-7dba8e4d18d0 |  |  |  |  |
|  | 75865e14-7f1f-4def-be8c-3d63b436de11 |  |  |  |  |
|  | 331a5878-7939-48ea-b58e-a5c71465aa30 | 2880 | [C>F](https://www.ebi.ac.uk/ProtVar/query?search=P78527%20C2880F) | Variant assessed as Somatic; MODERATE impact. (NCI-TCGA) | Adenomas and Adenocarcinomas |
|  | 2894830a-50f7-49c3-bacf-d32474637417 | 2935 | [E>*](https://www.ebi.ac.uk/ProtVar/query?search=P78527%20E2935*) | Variant assessed as Somatic; HIGH impact. (NCI-TCGA) | Adenomas and Adenocarcinomas |
|  | 3b196026-e571-4e6d-9d1c-31b1d4472d4d | 2971 | [Q>*](https://www.ebi.ac.uk/ProtVar/query?search=P78527%20Q2971*) | Variant assessed as Somatic; HIGH impact. (NCI-TCGA) | Squamous Cell Neoplasms |
|  | COSV100043245 |  |  |  |  |
|  | d97df25f-82e2-4079-97f6-d4562e9312b4 | 2994 | [W>*](https://www.ebi.ac.uk/ProtVar/query?search=P78527%20W2994*) | Variant assessed as Somatic; HIGH impact. (NCI-TCGA) | Adenomas and Adenocarcinomas |
|  | 49608101-19d9-45dd-8e46-52bd9f8edfd0 | 3025 | [P>Q](https://www.ebi.ac.uk/ProtVar/query?search=P78527%20P3025Q) | Variant assessed as Somatic; HIGH impact. (NCI-TCGA) | Adenomas and Adenocarcinomas |
|  | COSV58046109 |  |  |  |  |
|  | f8a459d5-f359-4cf4-b340-843474ad121a | 3074 | [Q>*](https://www.ebi.ac.uk/ProtVar/query?search=P78527%20Q3074*) | Variant assessed as Somatic; HIGH impact. (NCI-TCGA) | Lipomatous Neoplasms |
|  | 03b83c30-25c4-45ac-96e8-ab1d9257fd62 | 3093 | [Q>*](https://www.ebi.ac.uk/ProtVar/query?search=P78527%20Q3093*) | Variant assessed as Somatic; HIGH impact. (NCI-TCGA) | Squamous Cell Neoplasms |
|  | COSV100037711 |  |  |  |  |
|  | 82448c5a-0ca8-47e3-9e10-1ab45bf16df4 | 3137 | [E>*](https://www.ebi.ac.uk/ProtVar/query?search=P78527%20E3137*) | Variant assessed as Somatic; HIGH impact. (NCI-TCGA) | Adenomas and Adenocarcinomas |
|  | COSV100040773 |  |  |  |  |
|  | COSV100041884 |  |  |  |  |
|  | COSV100041291 | 3139 | [Q>*](https://www.ebi.ac.uk/ProtVar/query?search=P78527%20Q3139*) | Variant assessed as Somatic; HIGH impact. (NCI-TCGA) | Transitional Cell Papillomas and Carcinomas |
|  | ef9327c5-a088-476a-acd1-563179d77f9d |  |  |  |  |
|  | COSV58064018 | 3257 | [K>*](https://www.ebi.ac.uk/ProtVar/query?search=P78527%20K3257*) | Variant assessed as Somatic; HIGH impact. (NCI-TCGA) | Adenomas and Adenocarcinomas |
|  | b5496a95-9e9a-483c-a1c6-16b19bf86912 |  |  |  |  |
|  | 532d5274-fc66-486e-88f2-2ec6b147bb6c | 3265 | [E>*](https://www.ebi.ac.uk/ProtVar/query?search=P78527%20E3265*) | Variant assessed as Somatic; HIGH impact. (NCI-TCGA) | Gliomas |
|  | 33d7e211-edf6-4526-baca-552aa1a6caf2 | 3428 | [E>*](https://www.ebi.ac.uk/ProtVar/query?search=P78527%20E3428*) | Variant assessed as Somatic; HIGH impact. (NCI-TCGA) | Adenomas and Adenocarcinomas |
|  | COSV100038547 |  |  |  |  |
|  | COSV58064282 |  |  |  |  |
|  | cce0f20b-ae33-43f3-9b5c-583d02611eea |  |  |  |  |
|  | 2c94952a-c531-4566-a4da-080eb262a7c7 | 3432 | [S>*](https://www.ebi.ac.uk/ProtVar/query?search=P78527%20S3432*) | Variant assessed as Somatic; HIGH impact. (NCI-TCGA) | Squamous Cell Neoplasms |
|  | COSV58043224 |  |  |  |  |
|  | COSV58044908 | 3520 | [E>*](https://www.ebi.ac.uk/ProtVar/query?search=P78527%20E3520*) | Variant assessed as Somatic; HIGH impact. (NCI-TCGA) | Adenomas and Adenocarcinomas |
|  | c2a56983-ac98-4450-a7bc-e578f3bb7cb6 |  |  |  |  |
|  | ee8d806d-92cd-4c46-a664-f6e2027e7fce | 3600 | [P>L](https://www.ebi.ac.uk/ProtVar/query?search=P78527%20P3600L) | Variant assessed as Somatic; HIGH impact. (NCI-TCGA) | Myomatous Neoplasms |
|  | 2e658a26-28e6-4c88-a62b-1ff58b6d13eb | 3605 | [N>K](https://www.ebi.ac.uk/ProtVar/query?search=P78527%20N3605K) | Variant assessed as Somatic; HIGH impact. (NCI-TCGA) | Adenomas and Adenocarcinomas; Complex Epithelial Neoplasms |
|  | 2fba36f4-dd7c-4f4e-86a7-1ce7274cfe4e |  |  |  |  |
|  | 3f94f402-ee00-4c7c-bd7e-999b34cf06ce |  |  |  |  |
|  | 87cd3274-e074-4a35-b335-e5358dfa69bf |  |  |  |  |
|  | d300d561-9fc9-46da-bf33-c8987df2e2f0 |  |  |  |  |
|  | rs1284489077 |  |  |  |  |
|  | 9fdba8ea-d0e0-4650-b9a1-c2c6adc77d34 | 3639 | [E>*](https://www.ebi.ac.uk/ProtVar/query?search=P78527%20E3639*) | Variant assessed as Somatic; HIGH impact. (NCI-TCGA) | Adenomas and Adenocarcinomas |
|  | COSV58055592 |  |  |  |  |
|  | a7da89a3-b53b-4efd-8fb8-5c7692c4d989 |  |  |  |  |
|  | dd05be4b-7741-4f74-82b0-9958cd57114c |  |  |  |  |
|  |  | 3678 | [G>W](https://www.ebi.ac.uk/ProtVar/query?search=P78527%20G3678W) | Variant assessed as Somatic; HIGH impact. (NCI-TCGA) | Cystic, Mucinous and Serous Neoplasms |
|  | e774b1d6-a4c5-4f02-a1d9-ee9b96cc4fd2 |  |  |  |  |
|  | 74908a76-87ab-4bef-8fda-fe02b2da4457 | 3700 | [E>*](https://www.ebi.ac.uk/ProtVar/query?search=P78527%20E3700*) | Variant assessed as Somatic; HIGH impact. (NCI-TCGA) | Squamous Cell Neoplasms |
|  | COSV100038497 |  |  |  |  |
|  | 86117d2a-b07d-4d12-aebd-736c877b165f | 3733 | [R>*](https://www.ebi.ac.uk/ProtVar/query?search=P78527%20R3733*) | Variant assessed as Somatic; HIGH impact. (NCI-TCGA) | Cystic, Mucinous and Serous Neoplasms |
|  | COSV58065037 |  |  |  |  |
|  | rs1387336554 |  |  |  |  |
|  | 29c49baa-538d-4eb0-842f-6480ff8c9917 | 3781 | [C>*](https://www.ebi.ac.uk/ProtVar/query?search=P78527%20C3781*) | Variant assessed as Somatic; HIGH impact. (NCI-TCGA) | Ductal and Lobular Neoplasms |
|  |  |  |  |  |  |
|  | 79d2bad3-5c00-42c3-91b9-e30c05ac6c1d | 3802 | [L>*](https://www.ebi.ac.uk/ProtVar/query?search=P78527%20L3802*) | Variant assessed as Somatic; HIGH impact. (NCI-TCGA) | Adenomas and Adenocarcinomas |
|  | COSV58046495 |  |  |  |  |
|  | COSV58060361 | 3805 | [W>*](https://www.ebi.ac.uk/ProtVar/query?search=P78527%20W3805*) | Variant assessed as Somatic; HIGH impact. (NCI-TCGA) | Adenomas and Adenocarcinomas |
|  | e4bdd6b8-485c-4e5d-a2d4-fe070949be1c |  |  |  |  |
|  |  | 3817 | [L>*](https://www.ebi.ac.uk/ProtVar/query?search=P78527%20L3817*) | Variant assessed as Somatic; HIGH impact. (NCI-TCGA) | Adenomas and Adenocarcinomas |
|  | aafd923e-2651-4260-a5f1-b2d2c3629243 |  |  |  |  |
|  | 9a373792-babb-4744-a23c-eb4ee170e6de | 3846 | [M>C](https://www.ebi.ac.uk/ProtVar/query?search=P78527%20M3846C) | Variant assessed as Somatic; HIGH impact. (NCI-TCGA) | Adenomas and Adenocarcinomas |
|  | CGA novel | 3890 | [M>V](https://www.ebi.ac.uk/ProtVar/query?search=P78527%20M3890V) | Variant assessed as Somatic; HIGH impact. (NCI-TCGA) | Acute Lymphoblastic Leukemia |
|  | c4675b13-78f8-431c-8b9e-3c807d233421 |  |  |  |  |
|  | 4d831b11-374a-4ca2-b630-1b7a76027154 | 3895 | [E>*](https://www.ebi.ac.uk/ProtVar/query?search=P78527%20E3895*) | Variant assessed as Somatic; HIGH impact. (NCI-TCGA) | Adenomas and Adenocarcinomas |
|  | COSV100039937 |  |  |  |  |
|  | 087667e4-fccf-4367-adc1-8d04fe3cfde3 | 3904 | [F>L](https://www.ebi.ac.uk/ProtVar/query?search=P78527%20F3904L) | Variant assessed as Somatic; HIGH impact. (NCI-TCGA) | Squamous Cell Neoplasms |
|  | COSV58062662 |  |  |  |  |
|  | 110a19b8-50d4-4a30-b64b-1cd6b76debeb | 4024 | [G>R](https://www.ebi.ac.uk/ProtVar/query?search=P78527%20G4024R) | Variant assessed as Somatic; HIGH impact. (NCI-TCGA) | Adenomas and Adenocarcinomas; Cystic, Mucinous and Serous Neoplasms |
|  | 952132bc-8705-4e08-ad8f-c4f9642b1db0 |  |  |  |  |
|  | a1abe46c-34e2-4b88-8629-e30880f30643 |  |  |  |  |
|  | 9fdba8ea-d0e0-4650-b9a1-c2c6adc77d34 | 4030 | [E>*](https://www.ebi.ac.uk/ProtVar/query?search=P78527%20E4030*) | Variant assessed as Somatic; HIGH impact. (NCI-TCGA) | Adenomas and Adenocarcinomas |
|  | COSV58048222 |  |  |  |  |
|  | dd05be4b-7741-4f74-82b0-9958cd57114c |  |  |  |  |
|  | 1bde362c-602e-489e-bae3-8213d78344fc | 4037 | [N>K](https://www.ebi.ac.uk/ProtVar/query?search=P78527%20N4037K) | Variant assessed as Somatic; HIGH impact. (NCI-TCGA) | Adenomas and Adenocarcinomas |
|  | acce4569-0ec5-4988-9b95-1a93d56a5878 | 4041 | [R>D](https://www.ebi.ac.uk/ProtVar/query?search=P78527%20R4041D) | Variant assessed as Somatic; HIGH impact. (NCI-TCGA) | Squamous Cell Neoplasms |
|  | 9c6959c3-037c-41cc-bb2d-3757fae11675 | 4049 | [R>*](https://www.ebi.ac.uk/ProtVar/query?search=P78527%20R4049*) | Variant assessed as Somatic; HIGH impact. (NCI-TCGA) | Adenomas and Adenocarcinomas |
|  | COSV58051481 |  |  |  |  |
| PRKCZ | rs1431492301 | 96 | [F>S](https://www.ebi.ac.uk/ProtVar/query?search=Q05513%20F96S) | Variant assessed as Somatic; HIGH impact. (NCI-TCGA) | Cystic, Mucinous and Serous Neoplasms |
|  | af3558bc-428a-416d-8c0d-94a0e15b2abe |  |  |  |  |
|  | 613d08ba-b2a6-43d3-b1d8-39fcd37b3ae2 | 245 | [Q>*](https://www.ebi.ac.uk/ProtVar/query?search=Q05513%20Q245*) | Variant assessed as Somatic; HIGH impact. (NCI-TCGA) | Transitional Cell Papillomas and Carcinomas |
|  | COSV101057743 |  |  |  |  |
|  | 4d831b11-374a-4ca2-b630-1b7a76027154 | 463 | [E>*](https://www.ebi.ac.uk/ProtVar/query?search=Q05513%20E463*) | Variant assessed as Somatic; HIGH impact. (NCI-TCGA) | Adenomas and Adenocarcinomas |
|  | COSV101057663 |  |  |  |  |
|  | b79e7ab5-dcc8-4f9a-902a-2e2027e51ea9 | 562 | D>R | Variant assessed as Somatic; HIGH impact. (NCI-TCGA) | Adenomas and Adenocarcinomas |
| MET | 3a57f209-4e45-4ded-ae72-c86b5224e082 | 1 | [M>?](https://www.ebi.ac.uk/ProtVar/query?search=P08581%20M1?) | Variant assessed as Somatic; HIGH impact. (NCI-TCGA) | Adenomas and Adenocarcinomas |
|  | COSV59257638 | 108 | [S>*](https://www.ebi.ac.uk/ProtVar/query?search=P08581%20S108*) | Variant assessed as Somatic; HIGH impact. (NCI-TCGA) | Neoplasms, NOS |
|  | af33833c-22be-48a5-9288-6912fb36cdca |  |  |  |  |
|  | rs2116585856 |  |  |  |  |
|  | 9d340e26-4c72-46c5-9ecf-46b37cbd31c7 | 272 | [Q>*](https://www.ebi.ac.uk/ProtVar/query?search=P08581%20Q272*) | Variant assessed as Somatic; HIGH impact. (NCI-TCGA) | Squamous Cell Neoplasms |
|  | COSV100577245 |  |  |  |  |
|  | rs2116598580 |  |  |  |  |
|  | 99ea0f12-b3e5-4652-935a-4e0a0ec2a413 | 343 | [F>S](https://www.ebi.ac.uk/ProtVar/query?search=P08581%20F343S) | Variant assessed as Somatic; HIGH impact. (NCI-TCGA) | Adenomas and Adenocarcinomas |
|  | COSV100577213 | 348 | [Q>*](https://www.ebi.ac.uk/ProtVar/query?search=P08581%20Q348*) | Variant assessed as Somatic; HIGH impact. (NCI-TCGA) | Adenomas and Adenocarcinomas |
|  | ede913b4-1265-447d-9521-54b989b7da96 |  |  |  |  |
|  | rs1328110342 |  |  |  |  |
|  | 220825e7-c7df-4329-b980-9c9e3e51a575 | 416 | [Y>*](https://www.ebi.ac.uk/ProtVar/query?search=P08581%20Y416*) | Variant assessed as Somatic; HIGH impact. (NCI-TCGA) | Adenomas and Adenocarcinomas |
|  | rs745726656 |  |  |  |  |
|  | COSV59267516 | 717 | [S>*](https://www.ebi.ac.uk/ProtVar/query?search=P08581%20S717*) | Variant assessed as Somatic; HIGH impact. (NCI-TCGA) | Adenomas and Adenocarcinomas |
|  | rs2116930845 |  |  |  |  |
|  | COSV59257565 |  |  |  |  |
|  | c2a56983-ac98-4450-a7bc-e578f3bb7cb6 |  |  |  |  |
|  | 6d63e694-0052-48ea-9c78-41a9a9716b68 | 796 | [S>*](https://www.ebi.ac.uk/ProtVar/query?search=P08581%20S796*) | Variant assessed as Somatic; HIGH impact. (NCI-TCGA) | Adenomas and Adenocarcinomas |
|  | COSV59260726 |  |  |  |  |
|  | COSV59268873 |  |  |  |  |
|  | rs2116953986 |  |  |  |  |
|  | 9ee3ebc8-5688-4f32-ab39-78a39835fd1b | 911 | [W>*](https://www.ebi.ac.uk/ProtVar/query?search=P08581%20W911*) | Variant assessed as Somatic; HIGH impact. (NCI-TCGA) | Nevi and Melanomas |
|  | COSV59266196 |  |  |  |  |
|  | rs2116991269 |  |  |  |  |
|  | 61845078-b637-4257-a4a0-4aa782bba1f2 | 933 | [G>*](https://www.ebi.ac.uk/ProtVar/query?search=P08581%20G933*) | Variant assessed as Somatic; HIGH impact. (NCI-TCGA) | Adenomas and Adenocarcinomas |
|  | rs45604032 |  |  |  |  |
|  | 8c13e5b2-9ddc-445c-a60a-3323fcd90433 | 958 | [R>E](https://www.ebi.ac.uk/ProtVar/query?search=P08581%20R958E) | Variant assessed as Somatic; HIGH impact. (NCI-TCGA) | Adenomas and Adenocarcinomas |
|  | COSV59257569 | 994 | [E>*](https://www.ebi.ac.uk/ProtVar/query?search=P08581%20E994*) | Variant assessed as Somatic; HIGH impact. (NCI-TCGA) | Adenomas and Adenocarcinomas |
|  | COSV59270205 |  |  |  |  |
|  | c2a56983-ac98-4450-a7bc-e578f3bb7cb6 |  |  |  |  |
|  | rs761162957 | 1003 | [Y>*](https://www.ebi.ac.uk/ProtVar/query?search=P08581%20Y1003*) | Variant assessed as Somatic; HIGH impact. (NCI-TCGA) | Adenomas and Adenocarcinomas |
|  | 59e1b937-0c64-4550-9cd6-5c2af40e85bc |  |  |  |  |
|  | COSV59263576 |  |  |  |  |
|  | 05e3c3ce-13ab-4a21-9489-af8e2a71ec74 | 1112 | [L>*](https://www.ebi.ac.uk/ProtVar/query?search=P08581%20L1112*) | Variant assessed as Somatic; HIGH impact. (NCI-TCGA) | Adenomas and Adenocarcinomas |
|  | 23877617-55aa-4550-9cf0-7d6508d0180a | 1180 | [D>I](https://www.ebi.ac.uk/ProtVar/query?search=P08581%20D1180I) | Variant assessed as Somatic; HIGH impact. (NCI-TCGA) | Adenomas and Adenocarcinomas |
|  | COSV59258859 |  |  |  |  |
|  | 4271791f-ab84-4e76-a82e-a862c28d9652 | 1233 | [E>*](https://www.ebi.ac.uk/ProtVar/query?search=P08581%20E1233*) | Variant assessed as Somatic; HIGH impact. (NCI-TCGA) | Adenomas and Adenocarcinomas |
|  | 5209c916-4975-4b04-ae57-457045098b4e | 1319 | [C>*](https://www.ebi.ac.uk/ProtVar/query?search=P08581%20C1319*) | Variant assessed as Somatic; HIGH impact. (NCI-TCGA) | Cystic, Mucinous and Serous Neoplasms |
|  | COSV100577203 |  |  |  |  |
|  | rs2117110694 |  |  |  |  |
|  | eabdcc18-0c7c-4cea-b4f6-b08498314f02 | 1347 | [E>G](https://www.ebi.ac.uk/ProtVar/query?search=P08581%20E1347G) | Variant assessed as Somatic; HIGH impact. (NCI-TCGA) | Adenomas and Adenocarcinomas |
| CDK8 | e09ffbae-09da-41e6-80df-46bad7454638 | 27 | [V>S](https://www.ebi.ac.uk/ProtVar/query?search=P49336%20V27S) | Variant assessed as Somatic; HIGH impact. (NCI-TCGA) | Adenomas and Adenocarcinomas |
|  | 86117d2a-b07d-4d12-aebd-736c877b165f | 52 | [K>N](https://www.ebi.ac.uk/ProtVar/query?search=P49336%20K52N) | Variant assessed as Somatic; HIGH impact. (NCI-TCGA) | Cystic, Mucinous and Serous Neoplasms |
|  | 8c2214bf-daa9-4ea5-bf48-8c0cb0eba599 | 94 | [W>*](https://www.ebi.ac.uk/ProtVar/query?search=P49336%20W94*) | Variant assessed as Somatic; HIGH impact. (NCI-TCGA) | Ductal and Lobular Neoplasms |
|  | COSV67421058 |  |  |  |  |
|  | bc776ac0-2a4e-4ad1-9e1c-89af31bfc89f | 122 | [Q>*](https://www.ebi.ac.uk/ProtVar/query?search=P49336%20Q122*) | Variant assessed as Somatic; HIGH impact. (NCI-TCGA) | Plasma Cell Tumors |
|  | COSV101037124 | 178 | [R>*](https://www.ebi.ac.uk/ProtVar/query?search=P49336%20R178*) | Variant assessed as Somatic; HIGH impact. (NCI-TCGA) | Adenomas and Adenocarcinomas |
|  | COSV67420054 |  |  |  |  |
|  | a3284616-431e-4a2e-8bc0-21cfbd68f2db |  |  |  |  |
|  | rs181880846 |  |  |  |  |
|  | 676c18a9-1cf4-4708-b416-089744f4e5c5 | 300 | [H>Q](https://www.ebi.ac.uk/ProtVar/query?search=P49336%20H300Q) | Variant assessed as Somatic; HIGH impact. (NCI-TCGA) | Plasma Cell Tumors; Squamous Cell Neoplasms |
|  | 9c008d43-d30d-44d4-a188-fe2704243249 |  |  |  |  |
|  | 509d59ea-b22c-42bc-8937-147fa7557947 | 323 | [R>*](https://www.ebi.ac.uk/ProtVar/query?search=P49336%20R323*) | Variant assessed as Somatic; HIGH impact. (NCI-TCGA) | Adenomas and Adenocarcinomas |
|  | COSV67420037 |  |  |  |  |
|  | rs1876204313 |  |  |  |  |
|  | 3fd2fb26-c25c-4d3f-b644-2c503223176b | 346 | [F>C](https://www.ebi.ac.uk/ProtVar/query?search=P49336%20F346C) | Variant assessed as Somatic; HIGH impact. (NCI-TCGA) | Plasma Cell Tumors |
|  | 1a873714-42c9-4cc4-88a7-6016f4cfa0e2 | 356 | [R>*](https://www.ebi.ac.uk/ProtVar/query?search=P49336%20R356*) | Variant assessed as Somatic; HIGH impact. (NCI-TCGA) | Adenomas and Adenocarcinomas; Gliomas |
|  | COSV67423243 |  |  |  |  |
|  | a64f1650-2d4d-4084-bc03-a4cc012e233e |  |  |  |  |
|  | rs764161177 |  |  |  |  |
|  | COSV67423013 | 384 | [G>*](https://www.ebi.ac.uk/ProtVar/query?search=P49336%20G384*) | Variant assessed as Somatic; HIGH impact. (NCI-TCGA) | Adenomas and Adenocarcinomas |
|  | c28969c9-7e3a-4f87-aee0-83eadf2b8edd |  |  |  |  |
|  | COSV67423335 | 411 | [T>L](https://www.ebi.ac.uk/ProtVar/query?search=P49336%20T411L) | Variant assessed as Somatic; HIGH impact. (NCI-TCGA) | Squamous Cell Neoplasms |
|  | ed93a591-1c08-47cd-be6d-dac2b7519c9c |  |  |  |  |
|  | 834241cb-85d1-4663-877d-4dbf075d51e0 | 415 | [G>N](https://www.ebi.ac.uk/ProtVar/query?search=P49336%20G415N) | Variant assessed as Somatic; HIGH impact. (NCI-TCGA) | Adenomas and Adenocarcinomas |
|  | 3a4088fc-aac2-4505-83f5-c6b57a908d63 | 423 | [Q>*](https://www.ebi.ac.uk/ProtVar/query?search=P49336%20Q423*) | Variant assessed as Somatic; HIGH impact. (NCI-TCGA) | Nevi and Melanomas |
|  | COSV67422919 |  |  |  |  |
| PRPF6 | 3278c4c9-37a5-4cb9-8156-f08be2a69468 | 52 | [G>R](https://www.ebi.ac.uk/ProtVar/query?search=O94906%20G52R) | Variant assessed as Somatic; HIGH impact. (NCI-TCGA) | Adenomas and Adenocarcinomas |
|  | 5a1b5769-e296-4ee7-9ad7-5890f2bef6c3 | 99 | [E>*](https://www.ebi.ac.uk/ProtVar/query?search=O94906%20E99*) | Variant assessed as Somatic; HIGH impact. (NCI-TCGA) | Adenomas and Adenocarcinomas |
|  | 37be7ae3-5d7c-48a3-b2ef-ca3dedbf6cfc | 118 | [E>*](https://www.ebi.ac.uk/ProtVar/query?search=O94906%20E118*) | Variant assessed as Somatic; HIGH impact. (NCI-TCGA) | Adenomas and Adenocarcinomas |
|  | COSV99411572 |  |  |  |  |
|  | 264b54f9-5d9c-4fa0-909b-57f0b9050cae | 577 | [W>A](https://www.ebi.ac.uk/ProtVar/query?search=O94906%20W577A) | Variant assessed as Somatic; HIGH impact. (NCI-TCGA) | Adenomas and Adenocarcinomas |
|  | 156c09f1-1969-49e4-b8a7-4c21306fcf46 | 782 | [E>S](https://www.ebi.ac.uk/ProtVar/query?search=O94906%20E782S) | Variant assessed as Somatic; HIGH impact. (NCI-TCGA) | Adenomas and Adenocarcinomas |
| PTK2 | COSV61782940 | 49 | [Y>*](https://www.ebi.ac.uk/ProtVar/query?search=E7ESA6%20Y49*) | Variant assessed as Somatic; HIGH impact. (NCI-TCGA) | Adenomas and Adenocarcinomas |
|  | c2a56983-ac98-4450-a7bc-e578f3bb7cb6 |  |  |  |  |
|  | rs886465125 |  |  |  |  |
|  | 30f2bb86-7503-4987-ad8c-55b3fbeb3133 | 193 | [Q>*](https://www.ebi.ac.uk/ProtVar/query?search=E7ESA6%20Q193*) | Variant assessed as Somatic; HIGH impact. (NCI-TCGA) | Squamous Cell Neoplasms |
|  | COSV100571307 |  |  |  |  |
|  | COSV100570727 | 282 | [E>*](https://www.ebi.ac.uk/ProtVar/query?search=E7ESA6%20E282*) | Variant assessed as Somatic; HIGH impact. (NCI-TCGA) | Adenomas and Adenocarcinomas |
|  | d5e4b53f-1417-423f-b3c5-d65b25daf8f6 |  |  |  |  |
|  | 022a9534-695d-4513-9707-bfd6187e3598 | 470 | [R>*](https://www.ebi.ac.uk/ProtVar/query?search=E7ESA6%20R470*) | Variant assessed as Somatic; HIGH impact. (NCI-TCGA) | Adenomas and Adenocarcinomas |
|  | COSV61788072 |  |  |  |  |
|  | f6bbd768-5a05-483a-96e5-d6726dd3ce2c |  |  |  |  |
|  | rs1247173286 |  |  |  |  |
|  | COSV61786171 | 613 | [R>*](https://www.ebi.ac.uk/ProtVar/query?search=E7ESA6%20R613*) | Variant assessed as Somatic; HIGH impact. (NCI-TCGA) | Adenomas and Adenocarcinomas |
|  | dcc7e515-acb7-45ad-a94b-cf4169a2a703 |  |  |  |  |
|  | rs139298046 |  |  |  |  |
|  | 01e5472d-3c49-459b-8fb0-ad548266f272 | 641 | [R>*](https://www.ebi.ac.uk/ProtVar/query?search=E7ESA6%20R641*) | Variant assessed as Somatic; HIGH impact. (NCI-TCGA) | Adenomas and Adenocarcinomas |
|  | COSV61783815 |  |  |  |  |
|  | rs1336614447 |  |  |  |  |
|  | 0ef46d04-54a6-40ad-9959-ae95b586662a | 678 | [R>*](https://www.ebi.ac.uk/ProtVar/query?search=E7ESA6%20R678*) | Variant assessed as Somatic; HIGH impact. (NCI-TCGA) | Adenomas and Adenocarcinomas |
|  | COSV61784304 |  |  |  |  |
|  | COSV61788929 |  |  |  |  |
|  | ceeabfad-6e08-4bac-b9e5-14ce3fc4795f |  |  |  |  |
|  | 1945d47b-6ebe-4f41-9a9e-b11388daefc1 | 719 | [Q>*](https://www.ebi.ac.uk/ProtVar/query?search=E7ESA6%20Q719*) | Variant assessed as Somatic; HIGH impact. (NCI-TCGA) | Ductal and Lobular Neoplasms; Nevi and Melanomas |
|  | 9d241a6a-879e-498e-b6b0-7a5c45df7d4a |  |  |  |  |
|  | COSV61790053 |  |  |  |  |
|  | 3b20883e-ea60-4c63-8f61-acd6ed5a8125 | 808 | [Q>*](https://www.ebi.ac.uk/ProtVar/query?search=E7ESA6%20Q808*) | Variant assessed as Somatic; HIGH impact. (NCI-TCGA) | Adenomas and Adenocarcinomas |
|  | 73ba80a3-a225-4bbb-a2cc-ddf898f41ec6 |  |  |  |  |
|  | COSV61787476 |  |  |  |  |
|  | d3fe6c68-415b-4e08-9981-e05d05bf8e49 |  |  |  |  |
|  | e83b9578-ff82-4210-9468-64c792876884 |  |  |  |  |
|  | 92a1fff1-352b-46dd-a2fa-6ad7830b4b5d | 885 | [R>*](https://www.ebi.ac.uk/ProtVar/query?search=E7ESA6%20R885*) | Variant assessed as Somatic; HIGH impact. (NCI-TCGA) | Adenomas and Adenocarcinomas |
|  | COSV61784985 |  |  |  |  |
|  | a724dcab-7210-40ee-bfeb-29bc67ddc48c | 1025 | [R>*](https://www.ebi.ac.uk/ProtVar/query?search=E7ESA6%20R1025*) | Variant assessed as Somatic; HIGH impact. (NCI-TCGA) | Adenomas and Adenocarcinomas |
|  | a860d4f1-611d-4ffe-b822-f58682ab33c9 |  |  |  |  |
|  | rs878926321 |  |  |  |  |
| HGS | 5832d20f-e28d-43a4-ac73-dd426534381f | 86 | [K>R](https://www.ebi.ac.uk/ProtVar/query?search=O14964%20K86R) | Variant assessed as Somatic; HIGH impact. (NCI-TCGA) | Adenomas and Adenocarcinomas |
|  | c7c6c71e-2f81-4a73-ab37-b0c0c5f10135 | 295 | [M>C](https://www.ebi.ac.uk/ProtVar/query?search=O14964%20M295C) | Variant assessed as Somatic; HIGH impact. (NCI-TCGA) | Adenomas and Adenocarcinomas |
|  | c92eaa61-332c-4e6a-953b-ac3128cd04d4 | 304 | [A>P](https://www.ebi.ac.uk/ProtVar/query?search=O14964%20A304P) | Variant assessed as Somatic; HIGH impact. (NCI-TCGA) | Adenomas and Adenocarcinomas |
|  | 3a57f209-4e45-4ded-ae72-c86b5224e082 | 406 | [E>*](https://www.ebi.ac.uk/ProtVar/query?search=O14964%20E406*) | Variant assessed as Somatic; HIGH impact. (NCI-TCGA) | Adenomas and Adenocarcinomas |
|  | f893b03e-01e7-46f2-b6cb-c1aa063a6aed | 444 | [Q>S](https://www.ebi.ac.uk/ProtVar/query?search=O14964%20Q444S) | Variant assessed as Somatic; HIGH impact. (NCI-TCGA) | Adenomas and Adenocarcinomas |
|  | 36d3ceab-6440-4980-93a9-b30283a0568d | 483 | [A>R](https://www.ebi.ac.uk/ProtVar/query?search=O14964%20A483R) | Variant assessed as Somatic; HIGH impact. (NCI-TCGA) | Adenomas and Adenocarcinomas |
|  | 580780ec-2f2d-4bac-bc08-3874bfc4e047 |  |  |  |  |
|  | 5fcf98b0-1d6e-43a7-923e-5a4f8f567550 |  |  |  |  |
|  | 7fcdd604-b740-4661-9b74-c8a5529c5cf1 |  |  |  |  |
|  | COSV100230656 |  |  |  |  |
|  | c6c917b9-9936-4560-b932-cb3be3d4234c |  |  |  |  |
|  | rs1555699517 |  |  |  |  |
|  | COSV61270297 | 494 | [K>*](https://www.ebi.ac.uk/ProtVar/query?search=O14964%20K494*) | Variant assessed as Somatic; HIGH impact. (NCI-TCGA) | Adenomas and Adenocarcinomas |
|  | a063c22a-eb2c-4002-92c6-4deb253cf56d |  |  |  |  |
|  | 45aa6be2-0344-440e-bad9-71e31fc37e92 | 554 | [V>S](https://www.ebi.ac.uk/ProtVar/query?search=O14964%20V554S) | Variant assessed as Somatic; HIGH impact. (NCI-TCGA) | Transitional Cell Papillomas and Carcinomas |
|  | d37514ff-9795-4b3c-8920-d413ddaaf1e2 | 639 | [A>R](https://www.ebi.ac.uk/ProtVar/query?search=O14964%20A639R) | Variant assessed as Somatic; HIGH impact. (NCI-TCGA) | Adenomas and Adenocarcinomas |
|  | 1ca9529c-efd3-473f-9a23-15bb8a1946db | 649 | [Q>*](https://www.ebi.ac.uk/ProtVar/query?search=O14964%20Q649*) | Variant assessed as Somatic; HIGH impact. (NCI-TCGA) | Squamous Cell Neoplasms |
|  | 18eb3e5b-a608-4cdf-a8e7-afc181c456e6 | 750 | [Q>S](https://www.ebi.ac.uk/ProtVar/query?search=O14964%20Q750S) | Variant assessed as Somatic; HIGH impact. (NCI-TCGA) | Adenomas and Adenocarcinomas |
|  | rs748765403 |  |  |  |  |
|  | COSV100230496 | 755 | [V>C](https://www.ebi.ac.uk/ProtVar/query?search=O14964%20V755C) | Variant assessed as Somatic; HIGH impact. (NCI-TCGA) | Adenomas and Adenocarcinomas |
|  | acb88107-fc9f-435d-a355-815e070b5dd7 |  |  |  |  |
